# Characterization and optimization of 5´ untranslated region containing poly-adenine tracts in *Kluyveromyces marxianus* using machine-learning model

**DOI:** 10.1186/s12934-023-02271-3

**Published:** 2024-01-03

**Authors:** Junyuan Zeng, Kunfeng Song, Jingqi Wang, Haimei Wen, Jungang Zhou, Ting Ni, Hong Lu, Yao Yu

**Affiliations:** 1 State Key Laboratory of Genetic Engineering, School of Life Sciences, Fudan University, Shanghai, China; 2 Shanghai Engineering Research Center of Industrial Microorganisms, Shanghai, 200438 China

**Keywords:** 5´ UTR, Poly(A), *Kluyveromyces marixanus*, Machine-learning model, Heterologous protein expression

## Abstract

**Background:**

The 5´ untranslated region (5´ UTR) plays a key role in regulating translation efficiency and mRNA stability, making it a favored target in genetic engineering and synthetic biology. A common feature found in the 5´ UTR is the poly-adenine (poly(A)) tract. However, the effect of 5´ UTR poly(A) on protein production remains controversial. Machine-learning models are powerful tools for explaining the complex contributions of features, but models incorporating features of 5´ UTR poly(A) are currently lacking. Thus, our goal is to construct such a model, using natural 5´ UTRs from *Kluyveromyces marxianus*, a promising cell factory for producing heterologous proteins.

**Results:**

We constructed a mini-library consisting of 207 5´ UTRs harboring poly(A) and 34 5´ UTRs without poly(A) from *K. marxianus*. The effects of each 5´ UTR on the production of a GFP reporter were evaluated individually in vivo, and the resulting protein abundance spanned an approximately 450-fold range throughout. The data were used to train a multi-layer perceptron neural network (MLP-NN) model that incorporated the length and position of poly(A) as features. The model exhibited good performance in predicting protein abundance (average R^2^ = 0.7290). The model suggests that the length of poly(A) is negatively correlated with protein production, whereas poly(A) located between 10 and 30 nt upstream of the start codon (AUG) exhibits a weak positive effect on protein abundance. Using the model as guidance, the deletion or reduction of poly(A) upstream of 30 nt preceding AUG tended to improve the production of GFP and a feruloyl esterase. Deletions of poly(A) showed inconsistent effects on mRNA levels, suggesting that poly(A) represses protein production either with or without reducing mRNA levels.

**Conclusion:**

The effects of poly(A) on protein production depend on its length and position. Integrating poly(A) features into machine-learning models improves simulation accuracy. Deleting or reducing poly(A) upstream of 30 nt preceding AUG tends to enhance protein production. This optimization strategy can be applied to enhance the yield of *K. marxianus* and other microbial cell factories.

**Supplementary Information:**

The online version contains supplementary material available at 10.1186/s12934-023-02271-3.

## Introduction

The 5´ untranslated region (5´ UTR) is the segment of an mRNA that spans from the 5´ end to the position of the start codon (AUG). The 5´ UTR plays key roles in post-transcriptional regulation without altering the protein sequence, making it a favored target in genetic engineering and synthetic biology [1, 2]. The effects of the 5´ UTR on protein production are mediated by the multiple cis-regulatory elements it carries, including the 5´-cap structure [3], the translation initiation context [4, 5], upstream AUGs and upstream ORFs [6–8], internal ribosome entry sites (IRES) [9, 10], nucleotide preferences at positions immediately upstream of AUG [11, 12], secondary structures [13, 14], and G-quadruplexes [15]. These elements primarily regulate translation efficiency and mRNA stability.

Poly-adenine (poly(A)) tract is a common feature in the 5´ UTR. Approximately 28% of genes in *Saccharomyces cerevisiae*, 48% of genes in *Arabidopsis thaliana*, 39% of genes in *Drosophila melanogaster*, 9% of genes in mice, and 11% of genes in humans contain at least one poly(A) longer than 5 nt in the 5´ UTR [16]. Unlike the poly(A) tail added at the 3´ end of mRNA, the sequence encoding 5´ UTR poly(A) is embedded in the primary gene sequence and transcribed as part of the mature mRNA. The frequency of continuous poly(A)_n_ occurring is (1/4)^n^. Therefore, the occurrence of 5´ UTR poly(A) is not due to random base combinations but is linked to the function of the 5´ UTR.

The effect of 5´ UTR poly(A) on gene expression remains controversial in different circumstances. 5´ UTR poly(A) forms IRES for cap-independent translation of invasive growth genes in *S. cerevisiae* [17], genes related to pattern-triggered immunity in *Arabidopsis* plants [18], and *GFP* and *Luc* reporter genes in vitro [19, 20]. In a study utilizing a synthetic mRNA library containing over one million 5´ UTR variants, poly(A) was found to enable cap-independent translation in mammalian cells but destabilize mRNAs in the absence of translation in vitro [21]. The repression of translation by 5´ UTR poly(A) was also observed in the auto-regulation of PABP1 (Poly(A) binding protein 1) in mammalian cells [22], and *S. cerevisiae* [23]. The conflicting roles of 5´ UTR poly(A) might be related to its length. A bioinformatic analysis suggests that poly(A) shorter than 12 nt is correlated with improved translation efficiency, while poly(A) longer than 12 nt results in translation repression [24].

Constructing a machine learning model is a promising approach to explaining the complicated roles of 5´ UTR poly(A) in protein production. Several models of the 5´ UTR were constructed using different machine learning strategies, such as partial least squares (PLS) regression [25, 26], random forest [14, 27], support vector machine (SVM) [27], convolutional neural network (CNN) [6, 7, 28, 29], and transformer [30]. These models were successfully applied to predict the protein production driven by the 5´ UTR and to reveal the contribution of 5´ UTR elements to the protein production. To exclude background interference, the construction of a model requires building a randomly or deliberately designed 5´ UTR library that drives the expression of the same reporter gene. However, a similar analysis has not been performed using natural 5´ UTRs containing poly(A), which poses an obstacle to incorporating poly(A) as a novel feature into the machine learning model.

*Kluyveromyces marxianus* is an unconventional budding yeast species. Due to its long-standing safe association with human food, such as dairy products, grapes, and papaya, *K. marxianus* has been granted GRAS (Generally Regarded As Safe) and QPS (Qualified Presumption of Safety) status in the United States and Europe, respectively [31]. Besides its safety, *K. marxianus* possesses several features beneficial for industrial applications, including fast growth, thermotolerance, a broad spectrum of utilizable carbon sources, and a high capacity for secretion. Therefore, *K. marxianus* is a promising microbial cell factory for producing heterologous proteins, biofuels, and various chemicals [32, 33]. In *K. marxianus*, the deletion of a poly(A) tract inside the *INU1* 5´ UTR increased downstream protein production [34], while abolishing the poly(A) in the *LAC4* 5´ UTR reduced the leaky expression under glucose repression [35]. These results indicate that 5´ UTR poly(A) plays an important role in regulating protein production in *K. marxianus*.

In this study, we constructed a mini-library comprising 207 natural 5´ UTRs harboring poly(A) along with 34 5´ UTRs without poly(A). All the 5´ UTRs are from *K. marxianus*. The effect of each 5´ UTR on protein production was evaluated separately in vivo using a dual fluorescent reporter system. The obtained data were used to construct a multi-layer perceptron neural network (MLP-NN) model, in which poly(A) features were incorporated. The model demonstrated good performance in predicting protein abundance. The model suggests that the length of poly(A) is generally negatively correlated with protein production, while poly(A)s with a distance to AUG between 10 ~ 30 nt exhibit a weak correlation with improved protein production. Consistent with the model’s predictions, the deletions of poly(A)s upstream of 30 nt preceding AUG tended to enhance protein production, which was validated through the expression analysis of GFP and a feruloyl esterase (AnFaeA). These results suggest that incorporating poly(A) features into machine-learning models can enhance the accuracy of the prediction. The optimized strategy of the 5´ UTR proposed here could be applied to improve the yield of *K. marxianus* and other microbial cell factories.

## Materials and methods

### Strains and plasmids

*K. marxianus* strain, Fim-1ΔU [34], was used as a wild-type strain in this study. All plasmids used in this study are listed in Additional file 2: Table S1. All the primers used are listed in Additional file 2: Table S2. *HXT4* promoter (1351 bp) and 5´ UTR (149 bp) were inserted into an *Xho* I site immediately preceding the open reading frame (ORF) of *GFP* in LHZ676 [36], to obtain LHZ1137. The *HXT4* 5´ UTR of LHZ1137 was replaced by an *Xho* I site to obtain LHZ1138. Different natural 5´ UTRs were amplified using primers OZJY1F/R ~ OZJY240F/R and then inserted into the *Xho* I site of LHZ1138 by Gibson-assembly, resulting in the generation of LHZ1139 ~ LHZ1378. Mutations within 5´ UTRs were introduced by mutagenesis PCR using primers OZJY241F/R to OZJY302F/R, resulting in the generation of LHZ1379 ~ LHZ1440. The cassette of *P*_*INU1*_-*SS*_*INU1*_-*Est1E-His*_*6*_-*T*_*INU1*_ of pZP32 [34], was replaced by a cassette of *HXT4* promoter-*Sma* I- *SS*_*INU1−P10L*_- *AnFaeA*-*INU1* terminator. The *SS*_*INU1−P10L*_ coding an *INU1* signal peptide with a P10L mutation was amplified from pZP33 [34], and the ORF of *AnFaeA* was amplified from LHZ766 [37]. The resulting plasmid was named LHZ1441. The wild-type and mutant 5´ UTRs of *SSH4*, *INU1* and *KLMA_80280* were amplified from LHZ1182, LHZ1438, LHZ1164, LHZ1419, LHZ1158 and LHZ1439. Amplified 5´ UTRs were inserted into the *Sma* I site of LHZ1441 to obtain LHZ1442 ~ LHZ1447. The ORF of *Est1E* in pZP28 [34], was replaced by the ORF of *AnFaeA* amplified from LHZ733 [37], resulting in LHZ1448. Poly(A) inside *INU1* 5´ UTR in LHZ1448 was mutated by mutagenesis PCR using primers OZJY307F/R ~ OZJY320F/R, resulting in the generation of LHZ1449 ~ LHZ1462. The sequences of three backbone plasmids, LHZ1138, LHZ1441 and LHZ1448, are listed in Additional file 2: Table. S3.

### Nanopore RNAseq

Three parallel cultures were grown in YPD (2% w/v glucose, 2% w/v peptone; 1% w/v yeast extract) at 30 degrees for 16 h or 72 h. Cells were collected and total RNA was extracted by using ZR Fungal/Bacterial RNA MiniPrep (Zymo Research, R2014). RNA was subjected to Nanopore sequencing at Biomarker Technologies Inc.(Beijing, China). After low-quality reads (length < 500 bp, Q score < 7) were filtered out, 6.5 ~ 8.5 million clean reads were obtained for each sample. The reads were mapped to the FIM-1 reference genome [38], using minimap2, with more than 95% of the reads successfully mapped for each sample. Redundancy was conducted for each sample by filtering sequences with an identity below 0.9 and coverage below 0.85. Alignments showing differences only at the 5´ end were merged to obtain a gff file for each sample. By comparing these files with the FIM-1 reference gff annotation file using bedtools, additional sequences in transcriptions relative to the 5´ end of corresponding coding sequences (CDS) were extracted as the 5´ UTRs and matched to their corresponding genes. A gene may have multiple 5´ UTRs due to differences in transcription start sites and data processing. We identified the most frequent 5´ UTR(s) for each gene. In cases where multiple 5´ UTRs shared the same highest frequency, we selected the longest one. Transcripts per million (TPM) value was used as a measure of gene expression level [39], and was calculated for each gene in each sample. Sequences of 5´ UTRs are listed in Additional file 3 and TPM values of each gene are listed in Additional file 4.

### Proteomics analysis

Cells were collected as described in nanopore RNAseq. Cells were resuspended in 8 M guanidine HCl, 100 mM Tris HCl (pH 8.0) and then lysed by glass beads in a FastPrep-24 5G instrument (MP Biomedicals, USA). The supernatant was collected after centrifugation and subjected to the FASP digestion in Microcon PL-10 filters as described previously [40]. Nano LC-MS/MS analysis was performed using an EASY-nLC 1200 system (Thermo Fisher Scientific, USA) coupled to an Orbitrap Fusion Lumos mass spectrometer (Thermo Fisher Scientific, USA). A one-column system was adopted for all analyses. Samples were analyzed on a homemade C18 analytical column (75 μm i.d. × 25 cm, ReproSil-Pur 120 C18-AQ, 1.9 μm (Dr. Maisch GmbH, Germany)) [41]. The results were processed with the UniProt *K. marxianus* protein database (4926 entries, downloaded in 10/27/2020) and using the Mascot (version 2.7.0, Matrix Science) [42]. The mass tolerances were 10 ppm for precursor and fragment mass tolerance of 0.05 Da. Up to two missed cleavages were allowed. The carbamidomethylation on cysteine was set as a fixed modification, and acetylation on the protein N-terminal and oxidation on methionine as variable modifications. The significance threshold was *p* < 0.05, and the minimum number of significant unique sequence was 1. The exponentially modified protein abundance index (emPAI) was found to be approximately proportional to the logarithm of protein concentration [43], and was used for intra-sample comparison of protein abundance [44–48]. The emPAI values were calculated for all proteins and listed in Additional file 5.

### Calculation of relative entropy

The relative entropy was used to describe the enrichment and depletion of the base at a specific position [12, 25]. In a given sequence set, the relative entropy of base k at position i is denoted as ${E}_{set[i,k]}$, which is calculated as follows:

$${E}_{set[i,k]}={P}_{set[i,k]}\times {log}_{2}\left(\frac{{P}_{set[i,k]}}{{P}_{back[i,k]}}\right)$$

${P}_{set[i,k]}$ means the probability of the base $k$ at the position $i$ in the sequence $set$ of interest. ${P}_{back[i,k]}$ means the probability of the base $k$ at the position $i$ in the *background set*, which comprises all other sequences that are not included in the set of interest. In the graph, ${E}_{set[i,k]}$ is visualized as the height of the logo of base $k$ (A, C, G, or U), where over-representation and under-representation are indicated by positive and negative ${E}_{set[i,k]}$ values, respectively.

### Construction of mini-library and flow cytometry analysis

First, 5´ UTRs with a length of less than 200 nt were selected. Next, 5´ UTRs of genes that ranked in the top 20% based on their values of TPM, emPAI or emPAI/TPM, were selected. Then, 207 5´ UTR containing poly(A) (n ≥ 5) were randomly selected. As controls, 34 5´ UTRs without poly(A) were selected. The length of the longest continuous A tracts (n < 5) inside these 34 5´ UTRs was evenly distributed. A total of 241 5´ UTR were selected and cloned into LHZ1138 separately to construct the mini-library containing LHZ1137 and LHZ1139 ~ LHZ1378. Plasmids were transformed into Fim-1ΔU separately and transformants were selected on Synthetic dropout media without uracil (SC-Ura) plates [49]. Transformants were grown in 50 mL SC-Ura liquid medium for 72 h. Cells were washed and resuspended in 50 mM Tris-HCl (pH 7.0). A total of 100,000 cells were subjected to fluorescence-activated cell sorting (FACS) by gallios flow cytometer (Beckman Coulter, USA) and data were analyzed by Flowjo 2.0. The relative protein abundance of GFP was evaluated as the ratio of the average fluorescence intensity of GFP to that of mCherry. The experiment was performed with three biological replicates.

### Training and testing of models

For machine learning, a total of 15 features were extracted from each of 241 5´ UTR, in which 12 features were described previously [12]. Three additional features included the length of the 5´ UTR (5´ UTR length), the length of the longest poly(A) in 5´ UTR (poly(A) length), and the distance between the longest poly(A) tract and AUG (poly(A) position). The minimum free energy (MFE) of the entire 5´ UTR along with the first 50 nt in the ORF was calculated using RNAStructure [50]. Models were trained based on MLP-NN using a dataset comprising feature values of 241 5´ UTRs from the mini library, as well as the corresponding relative GFP abundance caused by these 5´ UTRs. The dataset was shown in Additional file 6. Among 241 5´ UTRs, 193 (80%) 5´ UTRs were selected to form the training set, and the other 20% were withheld for the test set. To optimize the hyperparameters of the MLP-NN model, the training set was then divided into 5 subsets for a 5-fold cross-validation. We investigated combinations of hyperparameters, including the number of dense layers and the number of units per layer. Upon reviewing the results, we opted for a setup consisting of 3 dense layers, each housing 300 units. The activation functions of layers were default (relu). The independent test set was used to evaluate the prediction accuracy of the model. The coefficient of determination (R^2^) was calculated to represent the prediction capability of the model. All model training and prediction processes were performed in Python 3.8 using TensorFlow 2.12.0. Python codes were available at https://github.com/CODdown/2023--MLP-NN.

### Sensitivity analysis of MLP-NN mode

The sensitivity analysis of the MLP-NN model was performed using Shapley Additive Explanation (SHAP), which illustrates the potential influence of features attributed to the model [51]. SHAP sensitivity analysis was performed in Python 3.8 using shap 0.41.0. Python codes were available at https://github.com/CODdown/2023--MLP-NN.

### Enzymatic assay of AnFaeA

LHZ1442 ~ LHZ1447 and LHZ1449 ~ LHZ1462 were transformed into Fim-1ΔU separately, and transformants were selected on SC-Ura plates. Transformants were grown for 72 h in SC-Ura + Glutamate medium, in which (NH4)_2_SO_4_ was replaced by 0.1% glutamate since (NH4)_2_SO_4_ inhibits the activity of AnFaeA. Supernatants were subjected to an enzymatic assay of AnFaeA as described previously [34]. The assay was performed with three biological replicates.

### Real-time PCR

Transformants were grown in SC-Ura liquid medium or SC-Ura + Glutamate liquid medium for 72 h. Cells were harvested and the total RNA was extracted as described in nanopore RNAseq. RNA was reverse transcribed using HiScript III All-in-one RT SuperMix Perfect for qPCR (Vazyme, R333-01), and cDNA was subjected to real-time PCR using ChamQ Universal SYBR qPCR Master Mix (Vazyme, Q711-02). The mRNA level of *SWC4* served as a control. The experiment was performed with three biological replicates. The real-time PCR data were analyzed following the 2^−ΔΔCt^ method. The relative mRNA level of the target gene was calculated using the following equation:

$${relative\, mRNA\, level}_{target}={2}^{-({Ct}_{target}-{Ct}_{SWC4})}$$

where the ${Ct}_{target}$ is the cycle threshold of the target gene (*GFP* or *AnFaeA*) and the ${Ct}_{SWC4}$ is the cycle threshold of a housekeeper gene *SWC4*.

## Results

### 5´ UTR poly(A)s are linked with mRNA levels and protein abundance in *K. marxianus*

**Fig. 1:**
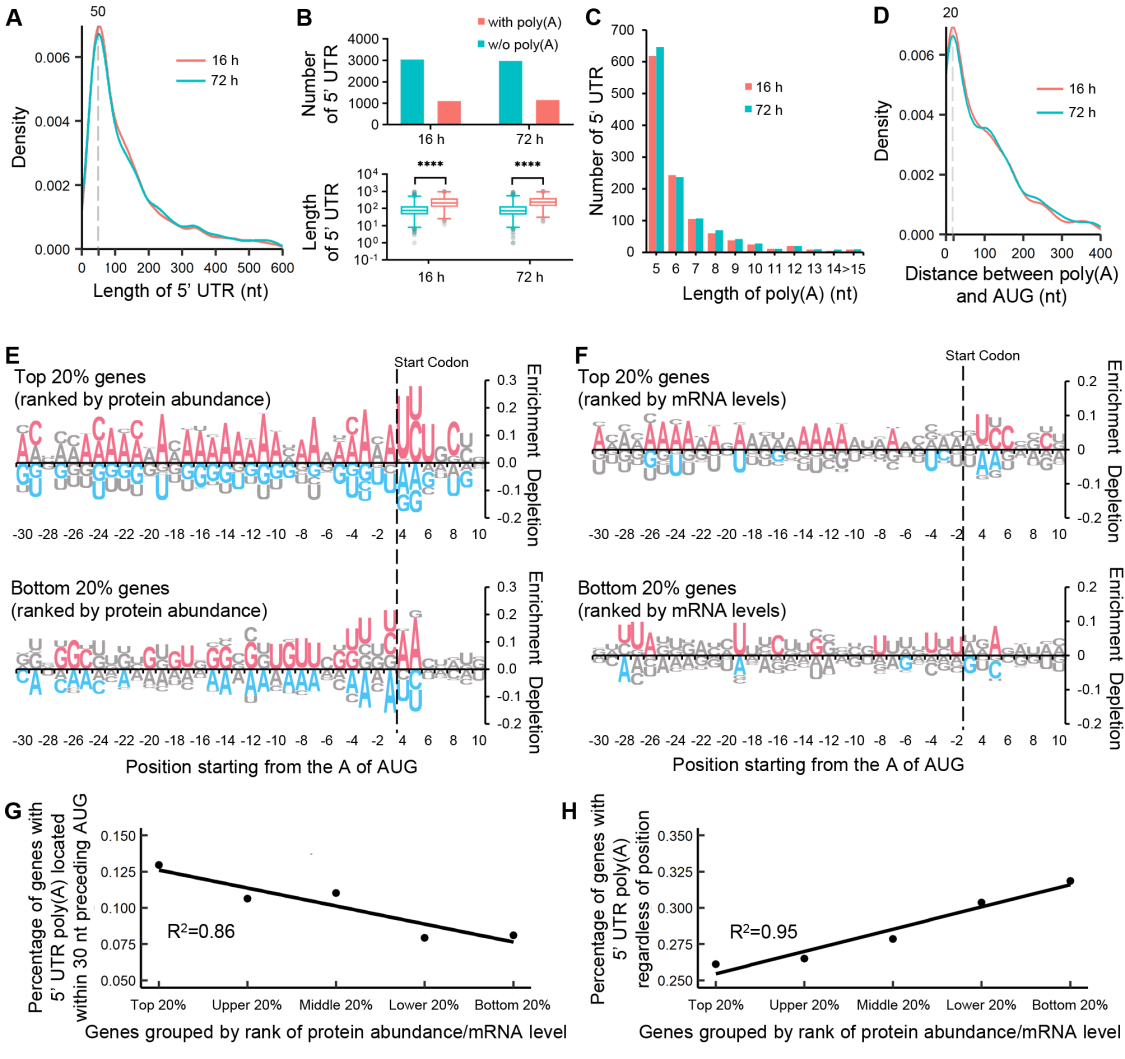
5´ UTR poly(A)s are linked with mRNA levels and protein abundance. (**A**) Distributions of 5´ UTR lengths in the *K. marxianus*. Cells were collected after 16 h and 72 h of growth, and subjected to the nanopore sequencing. A total of 4228 5´ UTRs from the 16 h sample and 4210 5´ UTRs from the 72 h sample were analyzed. A peak around 50 nt was indicated. (**B**) Number and length distributions of 5´ UTRs with or without poly(A) in 16 h and 72 h samples. The significance was assessed by a two-tails t-test. **** *p* < 0.0001. (**C**) Number of 5´ UTR containing poly(A) of various lengths. (**D**) Distribution of distance between 5´ UTR poly(A) and start codon (AUG). A peak around 20 nt was indicated. (**E**, **F**) Enrichment and depletion of four bases between 30 nt preceding and 7 nt after AUG (-30 ~ + 10) in different groups of genes. The genes were grouped based on the abundance of the encoded proteins (**E**) or the mRNA levels (**F**). The significance was assessed using a two-tailed Fisher’s exact test. Red or blue logos represented *p* < 0.05, while gray logos represented *p* > 0.05. (**G**, **H**) Correlation between the percentage of genes containing 5´ UTR poly(A) and the genes grouped by the ratio between protein abundance and mRNA level. Protein abundance and mRNA level were represented by emPAI and TPM values, respectively. The genes containing 5´ UTR poly(A) located within 30 nt preceding AUG were shown on the left (**G**), while those containing 5´ UTR poly(A) at any position were shown on the right (**H**)

To characterize the function of 5´ UTR poly(A) in *K. marxianus*, we collected cultures of *K. marxianus* after 16 h and 72 h for nanopore RNAseq and proteomics analysis. The time points of 16 h and 72 h mark crucial stages during fermentation. At 16 h, *K. marxianus* enters the late-exponential stage, and the culture is collected as seed culture for feed-batch fermentation [34]. After 72 h, *K. marxianus* enters the stationary phase. At this stage, fermentation is manually halted, and the culture is collected for expression analysis [34, 52]. Among the various 5´ UTRs of each gene, we selected the most frequently occurring 5´ UTR after redundancy for analysis. If there are multiple 5´ UTRs with the highest frequency, the longest one was chosen. As shown in Fig. 1A, the 5´ UTRs at both time points shared a similar length distribution, with a median length of 100 nt and an enriched peak at 50 nt. The median length of the 5´ UTR in *K. marxianus* was longer than that in *S. cerevisiae* (~ 50nt ) [53], suggesting a different mechanism to control 5´ UTR length among these two species. In this study, continuous adenine tracts equal to or longer than 5 nt within the 5´ UTR were designated as 5´ UTR poly(A)s. At both time points, around 25% of genes in *K. marxianus* harbored at least one 5´ UTR poly(A), a ratio comparable to that of *S. cerevisiae* [16]. The 5´ UTRs containing poly(A) were significantly longer than those without poly(A), suggesting that there is a higher occurrence of poly(A) in long 5´ UTRs (Fig. 1B). As the length of poly(A) increased, the number of 5´ UTRs containing such poly(A) decreased (Fig. 1C). The longest poly(A)s, composed of 23 As, were found in the 5´ UTRs of *NOP15* and *PFK27*. The median distance between the poly(A) and AUG was 76 nt and 86 nt for 16 h and 72 h, respectively, with peaks around 20 nt. In general, the 5´ UTRs and their poly(A) tracts shared similar characteristics at 16 h and 72 h, indicating a consistent regulation of the 5´ UTRs at both time points.

Given the frequent presence of poly(A) close to AUG, we calculated the enrichment and depletion of four bases between 30 nt preceding AUG (-30) and 10 nt after AUG (+ 10) in genes ranked by the abundance of encoded proteins. The emPAI exhibits a linear correlation with the logarithm of protein concentration [43]. For the 46 proteins in mouse whole cell lysate, the average deviation percentages of emPAI-based abundances from the actual values were within 63% [43]. Abundances of 40 proteins in *Escherichia coli* cytosol measured by the emPAI method correlated well with those determined through isotope dilution of a control lysate (R^2^ = 0.84) [54]. Additionally, emPAI-based protein concentration is automatically available for all proteins identified by MS without any additional experimental setup. Given these advantages, emPAI was employed in this study to indicate protein abundance. In the region around AUG, a Kozak sequence (A A/C A A/C A (AUG) U C/U C) was enriched in the top 20% of genes (Fig. 1E, upper panel). The Kozak sequence of *K. marxianus* closely resembled that of *S. cerevisiae*, indicating the reliability of our analysis [11, 55]. Regarding the upstream sequence, residue A was favored, but G and U were loathed within 30 nt preceding AUG in the top 20% of genes, ranked by the abundance of proteins encoded by genes (Fig. 1E, upper panel). The opposite trend was observed in the bottom 20% of genes (Fig. 1E, lower panel). Notably, the favored As in this region tended to cluster together to form poly(A) tracts around 20 nt preceding AUG, consistent with the peaks of poly(A) distribution shown in Fig. 1D. A similar pattern was observed in genes ranked by mRNA levels, as residue A was favored and tended to cluster around 20 nt before the AUG codon in the top 20% of genes (Fig. 1F, upper panel). In contrast, we did not observe any enrichment of poly(A) tracts between 100 ~ 30 nt preceding AUG in top 20% of genes ranked by either protein abundance or mRNA levels (Additional file 1: Fig S1).

To further identify the link between poly(A) tracts and protein abundance, we calculated the percentage of genes containing 5´ UTR poly(A) in groups of genes distinguished by a ratio between protein abundance and mRNA levels. This ratio served as a rough indicator of translation efficiency. Regarding the 5´ UTR poly(A) located within 30 nt preceding AUG, the percentage of genes containing this 5´ UTR poly(A) was positively correlated with the protein/mRNA ratio (Fig. 1G), suggesting that poly(A)s located close to AUG increase translation efficiency. However, the percentage of genes containing 5´ UTR poly(A) at any position was negatively correlated with the protein/mRNA ratio (Fig. 1H), suggesting that 5´ UTR poly(A)s generally inhibit translation. Consistent results were obtained when analyzing the relationship between poly(A) and protein/mRNA ratio in ungrouped genes. The average protein/mRNA ratio in genes with 5´ UTR poly(A) was significantly lower than that in genes without 5´ UTR poly(A) (Additional file 1: Fig S2A). The average protein/mRNA ratio in genes containing 5´ UTR poly(A) located within 30 nt preceding AUG was significantly higher than that in genes lacking this 5´ UTR poly(A) (Additional file 1: Fig S2B). The contradictory results between total poly(A) and poly(A) close to AUG suggest that the effect of poly(A) on protein production is position-dependent.

### Evaluate effects of 5´ UTR poly(A)s on protein production by a dual-reporter system

**Fig. 2:**
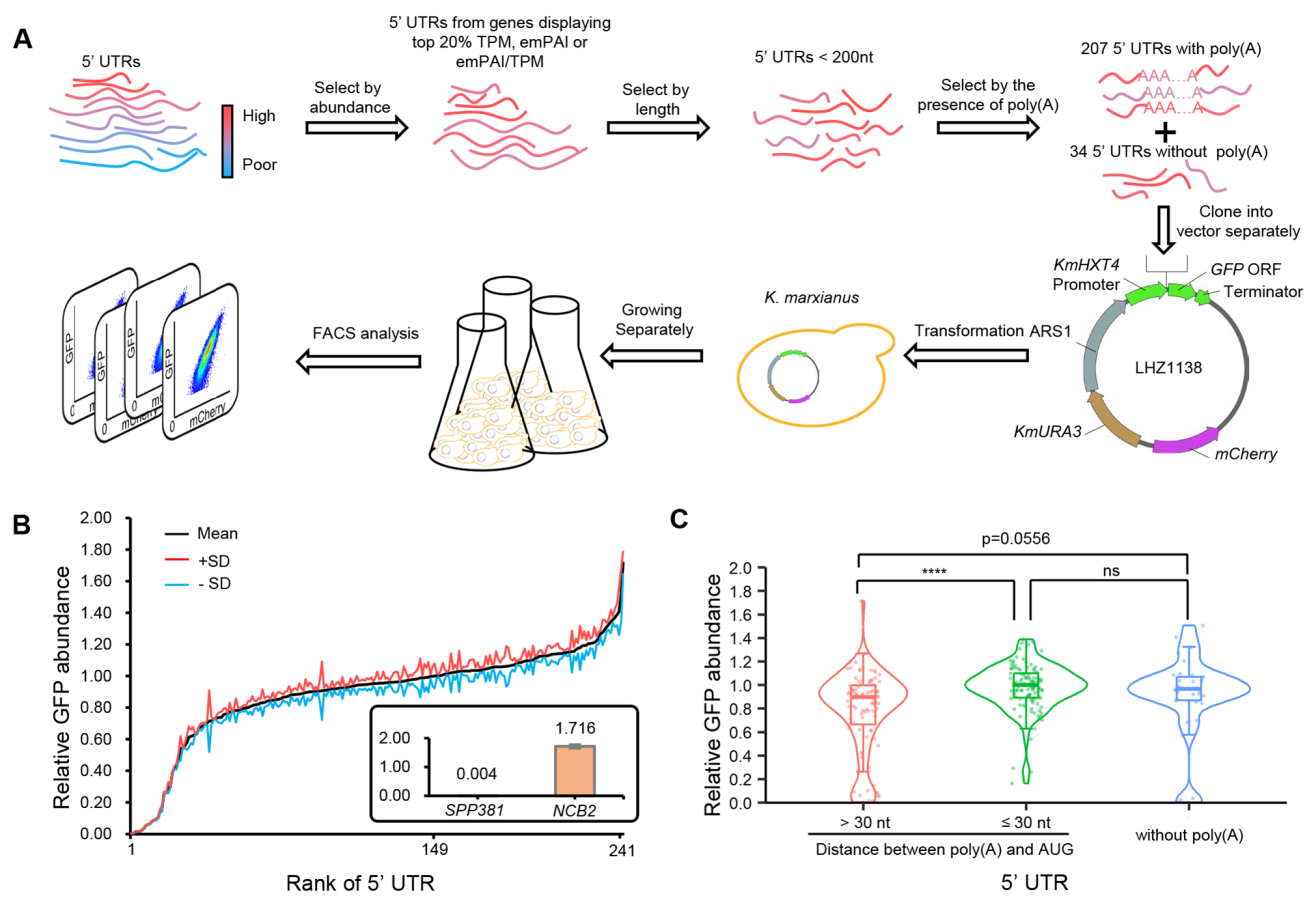
Evaluation of 5´ UTR poly(A)s through a dual-reporter system. (**A**) Flow chart of the dual-reporter system used to evaluate the effect of 5´ UTRs on protein production. Natural 5´ UTRs from abundant genes were randomly selected and cloned into separate vectors. The constructed plasmids were then transformed into *K. marxianus*, and the hosts were cultured separately for 72 h before FACS analysis. (**B**) Relative GFP abundance caused by 5´ UTRs. The relative GFP abundance was calculated as GFP/mCherry, where the relative GFP abundance caused by *HXT4* 5´ UTR was designated as 1. The mean relative GFP abundance of each 5´ UTR was ranked in ascending order, with the standard deviations (SD) shown (n = 3). The insert displayed 5´ UTRs that caused the lowest and highest relative GFP abundance. (**C**) Comparison of the relative GFP abundance caused by 5´ UTRs with poly(A) located at different positions. The significance was assessed using a two-tailed t-test. ****p < 0.0001; ns, *p* > 0.05

Evaluating the effects of 5´ UTR poly(A) on protein production based on transcriptomic and proteomic data was interfered with by gene context, including the promoter, ORF, and terminator. To reduce this interference, we constructed a dual-reporter system (Fig. 2A). We screened for natural 5´ UTRs with high abundance and a length of less than 200 nt. Among these 5´ UTRs, we selected a total of 207 5´ UTRs containing poly(A) as well as 34 5´ UTRs without poly(A) as controls. Each 5´ UTR was then cloned separately into LHZ1138 between a strong *HXT4* promoter and the ORF of *GFP*, with the vector containing a centromeric sequence (ARS1) to maintain a single copy in vivo and a cassette to express mCherry constitutively. The constructed plasmids were transformed into *K. marxianus*, cultured separately, and subjected to FACS. The abundance of GFP was normalized by the amount of mCherry.

A total of 241 5´ UTRs resulted in a broad range of relative GFP abundance (Fig. 2B). Compared to the natural 5´ UTR of *HXT4*, the lowest relative GFP abundance caused by the 5´ UTR of *SPP381* was 0.004 (Fig. 2B, insert). Despite mRNA and protein levels of *SPP381* being abundant, the low relative GFP abundance caused by its 5´ UTR indicates that the strong effect of gene context bypasses the effect of 5´ UTR. In contrast, the highest relative abundance caused by 5´ UTR of *NCB2* was 1.76 (Fig. 2B, insert). The relative GFP abundance caused by 5´ UTRs spanned an approximately 450-fold range, indicating the great potential of 5´ UTRs in regulating the production of proteins.

The mean relative abundance caused by 5´ UTRs that contain poly(A) within 30 nt preceding AUG was significantly higher than those contain poly(A) beyond 30 nt (*p* < 0.0001) (Fig. 2C). The result suggest the effect of 5´ UTR poly(A) on protein production varies depending on its position around − 30 nt, which was consistent with data in Fig. 1G. Meanwhile, the mean relative abundance of 5´ UTRs that contain poly(A) beyond 30 nt was slightly lower than that of 5´ UTRs without poly(A) (*p* = 0.0556), suggesting poly(A)s located distantly to AUG show negative effects on protein production. 5´ UTRs without poly(A) did not show a significant difference compared to 5´ UTRs with poly(A) smaller than 30 nt.

### Machine-learning model reveals the negative and position-dependent effect of 5´ UTR poly(A) on protein production

**Fig. 3:**
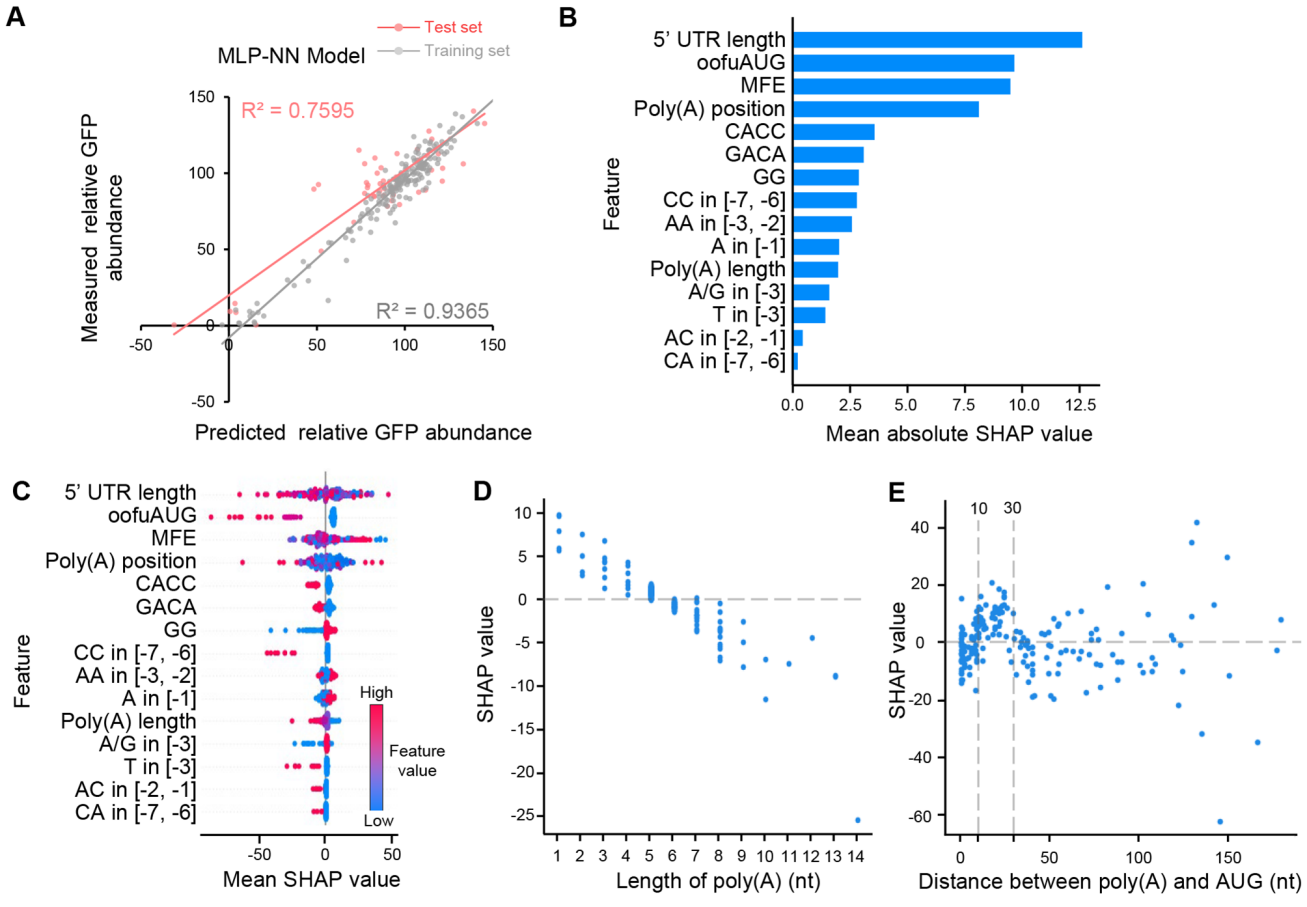
Construction and analysis of a machine-learning model that predicts the GFP abundance by features of 5´ UTR. (**A**) Validation of the MLP-NN model. The plot compared the measured versus the predicted relative GFP abundance, with R^2^ for the train and test sets included. (**B**) 5´ UTR features ranked by their mean absolute SHAP values. Mean SHAP values were obtained by performing sensitivity analysis on the MLP-NN model. Description of the features: 5´ UTR length, length of 5´ UTR; oofuAUG, number of out-of-frame upstream AUGs and upstream ORFs; MFE, minimum free energy; poly(A) position, the distance between the longest poly(A) tract and AUG; CACC, the presence of at least one CACC motif in the 5´ UTR; GACA, the presence of at least one GACA motif in the 5´ UTR; GG, the presence of at least one GG motif in the 5´ UTR; CC in [-7, -6], the presence of the motif CC at position [-7, -6] relative to the position of AUG; AA in [-3, -2], the presence of the motif AA at position [-3, -2]; A in [-1], the presence of the A at position − 1; poly(A) length, length of the longest poly(A) in 5´ UTR; A/G in [-3], the presence of the A or G at position − 3; T in [-3], the presence of the T at position − 3; AC in [-2, -1], the presence of the motif AC at position [-2, -1]; CA in [-7, -6], the presence of the motif CA at position [-7, -6]. (**C**) The relationship between the values of 5´ UTR features and SHAP values. Red and blue dots indicated high and low feature values, respectively. (**D**) A negative correlation between SHAP value and the length of poly(A). (**E**) The relationship between SHAP value and the position of poly(A). The majority of SHAP values were positive at distances between 10 and 30 nt, as indicated

The data obtained through the dual-reporter system was used to train a predictive model of 5´ UTR, which included the twelve features used in the training of previous models [12, 26]. These features included out-of-frame upstream AUG and upstream ORF (oofuAUG), MFE, and nucleotide preferences at the position immediately upstream of AUG. In addition, three new features were incorporated, including 5´ UTR length, poly(A) length, and poly(A) position. To include the data of 34 5´ UTRs without a poly(A) longer than 5 nt, the longest continuous As in these 5´ UTRs were used as poly(A)s to calculate relevant features. Therefore, a total of 241 5´ UTRs with 15 features each, along with relative GFP abundance caused by each 5´ UTR, constituted the dataset for model construction (Additional file 6). The dataset was randomly divided into two subsets. One subset, consisting of 15 feature values and the corresponding relative GFP abundance values of 193 5´ UTRs, was utilized as a training set to calibrate the model based on MLP-NN after optimizing hyper-parameters using a 5-fold cross-validation. Following the calibration, the remaining data of 48 5´ UTRs was employed as a test set to evaluate the predictive performance of the model. Model prediction reveals that a linear fit between the observed abundance and predicted abundance in the test set yielded an R^2^ of 0.7595 (Fig. 3A), indicating that the MLP-NN model can successfully predict the protein abundance caused by 5´ UTRs. We conducted four additional train-test splits to perform training and validation, resulting in the acquisition of 4 additional MLP-NN models. The average R^2^ for test set predictions of 5 models, including one original and four additional models, was 0.7290 (Additional file 1: Fig S3A). When new features (5´ UTR length, poly(A) length, and poly(A) position) were excluded from five distinct training-test splits, the average R^2^ decreased to 0.6403 (Additional file 1: Fig S3B), suggesting incorporation of these three features into the training process improves the model’s performance. In addition, we also built models based on SVM and random forest. However, both models resulted in lower R^2^ values for the test set (Additional file 1: Fig S4). These results suggest that, compared with the SVM and random forest models, the MLP-NN model is more effective at accurately capturing the association between 5´ UTR features and protein abundance. To assess our model’s competency in predicting protein production in other yeasts, we utilized a library comprising half a million 50-nt-long random 5´ UTRs previously tested in *S. cerevisiae* [7]. The impact of each 5´ UTR on *HIS3* production was assessed by measuring the enrichment of cells harboring the 5´ UTR after cultivation in selection media [7]. From this library, we selected 700 5´ UTRs containing poly(A) and 115 5´ UTRs lacking poly(A). The ratio of 700:115 was comparable to that in our 5´ UTR library. Employing the MLP-NN model, we predicted the enrichment of each 5´ UTR, yielding an R^2^ of 0.503 between predicted and measured enrichments (Additional File 1: Fig S5). The relatively low R^2^ could be attributed to differences between *S. cerevisiae* and *K. marxianus*. Additionally, our model might have underperformed in predicting enrichment, which served as an indirect indicator of protein abundance [7].

Based on the MLP-NN model, we conducted a sensitivity analysis to evaluate the impacts of features on protein abundance. SHAP (SHapley Additive exPlanations) values were calculated for each feature, and negative and positive SHAP values indicate negative and positive impacts on protein abundance, respectively, while the absolute value reflects the magnitude of the impact. Based on the rank of absolute SHAP values, 5´ UTR length was found to be the most influential feature on protein abundance (Fig. 3B). The feature of oofuAUG ranked as the second most influential feature (Fig. 3B), which is expected given that out-of-frame upstream AUGs and upstream ORFs have been shown to significantly impact translation efficiency [6–8]. Another important mRNA feature, MFE, ranked as the third most influential feature (Fig. 3B). The poly(A) position ranked fourth, indicating that the distance between poly(A) and AUG has an important impact on protein abundance (Fig. 3B). The impact of poly(A) length was relatively small, ranking 11th out of the total 15 features (Fig. 3B).

To visualize the relationship between feature values and SHAP values, red and blue dots were used to mark high and low feature values, respectively (Fig. 3C). High values of 5´ UTR length were found to be strongly correlated with negative SHAP values (Fig. 3C). The result indicates that the length of the 5´ UTR generally has a negative impact on protein abundance, possibly because longer 5´ UTRs have a greater tendency to form secondary structures that hinder ribosomal scanning [13]. In contrast, less negative MFE indicates a less stable secondary structure [56], leading to positive SHAP values. As expected, increased values of oofuAUG were strongly correlated with negative SHAP values, indicating the strong negative effects of out-of-frame upstream AUGs and upstream ORFs on translation [6–8]. Overall, most correlations between feature values and SHAP values were consistent with previous studies [12], indicating that the regulatory mechanisms of 5´ UTR features are conserved in *K. marxianus*. The relationships between poly(A) features and SHAP values were examined in detail. The length of poly(A) was found to be negatively related to the SHAP value (Fig. 3D). In most cases, poly(A) longer than 5 nt was associated with a negative SHAP value. The result indicates that poly(A) generally acts as a negative regulator of protein production, with longer poly(A) resulting in a greater negative effect on protein abundance. In Fig. 3E, SHAP values quantified the impact of poly(A) at each position on the model’s output, which might uncover differences obscured by the comparison of mean GFP production shown in Fig. 2C. There was no linear correlation between the poly(A) positions and SHAP values, but poly(A)s with a distance to AUG between 10 ~ 30 nt showed a correlation with positive SHAP values (Fig. 3E). A similar relationship between SHAP values and poly(A) features was detected in the sensitivity analysis using four models trained with additional training-test splits, reflecting the reliability of both the model construction and sensitivity analysis (Additional file 1: Fig S6). The results suggest that poly(A)s in this region may improve protein production. It was consistent with the opposite behaviors between the total 5´ UTR poly(A)s and 5´ UTR poly(A)s located close to AUG (Fig. 1G, H), indicating that the effect of poly(A) on protein production is dependent on its position.

### Optimization of 5´ UTR poly(A) to improve protein production with guidance of the machine-learning model

**Fig. 4:**
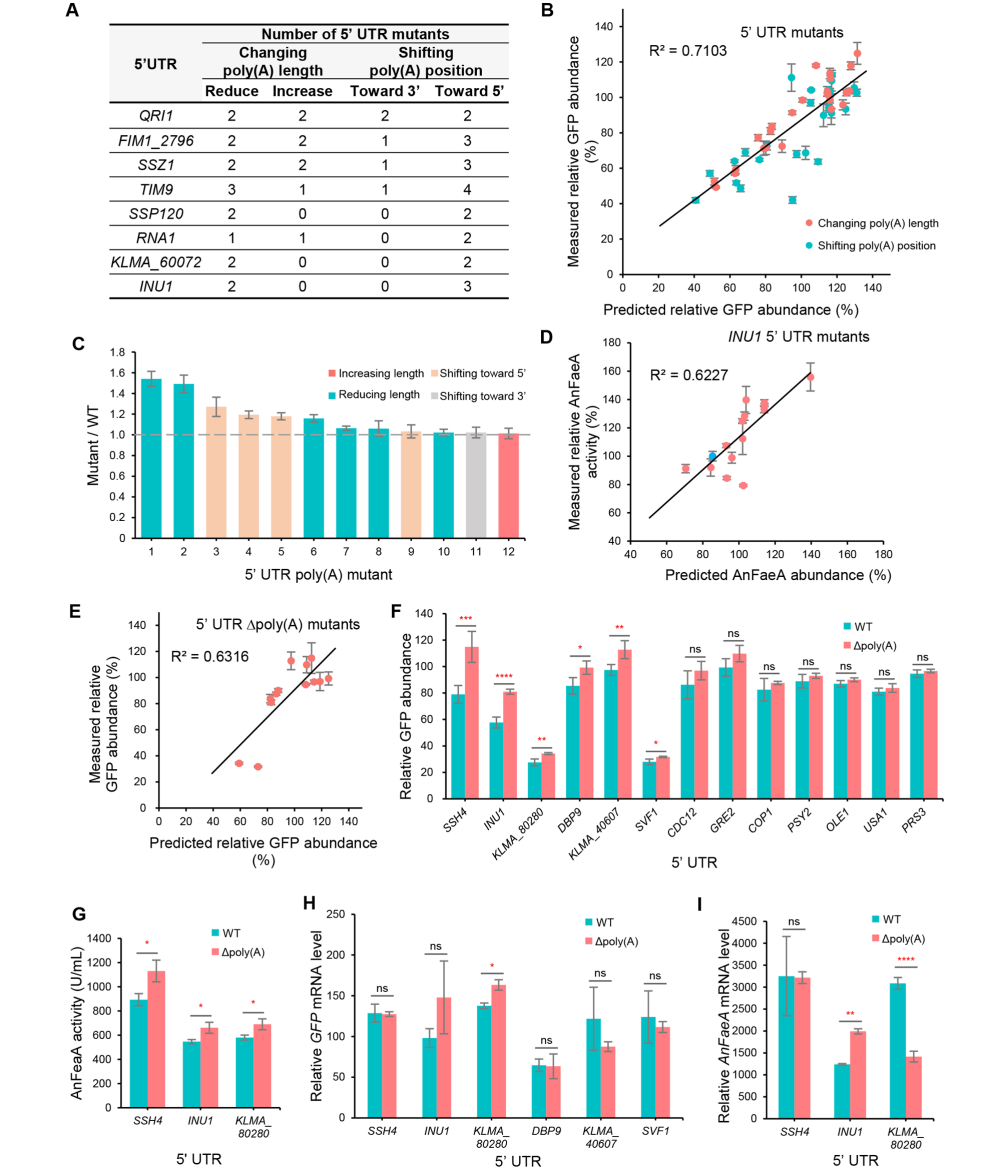
Increasing protein production by reducing or deleting 5´ UTR poly(A) with the guidance of the machine-learning model. (**A**) Summary of 5´ UTR mutants. The mutants were divided into two groups: one with changes in poly(A) length, and the other with shifts of the poly(A) position. (**B**) Plot representing the measured versus predicted relative GFP abundance of 5´ UTR mutants in (**A**). The value of R^2^ was shown in the plot. Standard deviations (SD) of measured abundance were shown (n = 3). Mutants that changed the poly(A) length and position were labelled in red and green, respectively. (**C**) Ratio between relative GFP abundance caused by mutant 5´ UTRs and that caused by wild-type 5´ UTRs. The mutants were ranked in descending order based on the ratios. Four types of mutants were distinguished by different colors. Mean ± SD was shown (n = 3). (**D**) Plot representing the measured AnFaeA activity versus predicted AnFaeA production in poly(A) mutants of *INU1* 5´ UTR. The value of R^2^ was shown in the plot. SD of measured abundance were shown (n = 3). The point representing the wild-type 5´ UTR of *INU1* was colored blue. (**E**) Plot representing the measured versus predicted relative GFP abundance caused by 5´ UTR Δpoly(A) mutants. The value of R^2^ was shown in the plot. Mean ± SD of measured abundance was shown (n = 3). (**F**) Comparison between the relative GFP abundance caused by 5´ UTR Δpoly(A) mutants and that caused by the wild-type 5´ UTRs. Mean ± SD was shown (n = 3). The significance was assessed by a two-tailed t-test. **** *p* < 0.0001; *** *p* < 0.001; ** *p* < 0.01; * *p* < 0.05; ns > 0.05. (**G**) Comparison between the AnFaeA activity caused by 5´ UTR Δpoly(A) mutants and that caused by the wild-type 5´ UTRs. Enzymatic activity was measured after culturing for 72 h. Mean ± SD was shown (n = 3). The significance was assessed by a two-tailed t-test. * *p* < 0.05. (**H**, **I**) Comparison between relative mRNA levels of *GFP* (**H**) or *AnFaeA* (**I**) expressed by 5´ UTR Δpoly(A) mutants and those expressed by wild-type 5´ UTRs. mRNA was extracted from samples described in (**E**) and (**F**) and subjected to qPCR analysis. The mRNA levels of *GFP* or *AnFaeA* were normalized with the mRNA level of *SWC4*. Mean ± SD was shown (n = 3). The significance was assessed by a two-tailed t-test. **** *p* < 0.0001; ** *p* < 0.01; * *p* < 0.05; ns > 0.05

The MLP-NN model was trained using data from natural 5´ UTRs. To validate the predictive accuracy of the model on non-natural sequences, 5´ UTRs from 8 genes were randomly selected and their poly(A) tracts were altered in length or position, resulting in a total of 50 5´ UTR mutants (Fig. 4A). The MLP-NN model predicted the relative GFP abundance caused by the 5´ UTR mutants and these predictions were compared with the experimental measurements. The results showed that the model’s predictions on 5´ UTR mutants were accurate, with an R^2^ value of 0.7103 (Fig. 4B). When ranking the ratio between the relative GFP abundance caused by the 5´ UTR mutants and that caused by the corresponding wild-type 5´ UTRs, 6 of the top 12 mutants with the largest fold changes contained a reduced length of poly(A) (Fig. 4C). These results suggest that reducing the poly(A) tracts tends to improve protein production, which is consistent with the observation that the length of poly(A) is negatively related to protein production (Fig. 3D). To validate the accuracy of the model’s predictions regarding the production of proteins other than GFP, alterations were made to 5´ UTR poly(A) in a multiple-copy plasmid [34], which drives secretory expression of a feruloyl esterase (AnFaeA) through the promoter and 5´ UTR of *INU1*. A total of 14 5´ UTR mutants were constructed. The secretory activities of AnFaeA caused by these *INU1* 5´ UTR mutants were measured and compared with that caused by wild-type *INU1* 5´ UTR. The measured relative activities of AnFaeA exhibited a linear correlation with the predicted values (R^2^ = 0.6227) (Fig. 4D). Therefore, these results suggest that the model is capable of predicting the production of different proteins in *K. marxianus*.

Compared to reducing the length of the 5´ UTR poly(A), deletion of the full poly(A) tract is more applicable in sequence engineering and might lead to a more dramatic effect on protein production. To avoid interfering with the potential positive effect of poly(A) with a distance to AUG between 10 ~ 30 nt (Fig. 3E), we proposed a strategy to delete poly(A) upstream of 30 nt preceding AUG. Thirteen 5´ UTRs containing poly(A) upstream of -30 nt were selected and deletions of poly(A)s in these 5´ UTRs were predicted to increase protein production by the MLP-NN model. These 5´ UTR Δpoly(A) mutants were constructed and measured. The measured relative GFP abundance caused by the 5´ UTR Δpoly(A) mutants showed a decent linear fit with predicted abundance (R^2^ = 0.6316), again proving the accuracy of the model’s prediction (Fig. 4E). Among the 13 5´ UTR Δpoly(A) mutants, 6 mutants caused a significant increase in the GFP abundance compared to the wild-type 5´ UTRs, while the remaining 7 mutants showed the same GFP abundance as the wild-type 5´ UTRs (Fig. 4F). Therefore, approximately 50% of the poly(A) mutants increased protein production. To verify the applicability of this strategy in different sequence contexts, the top three 5´ UTRs (*SSH4*, *INU1*, *KLMA_80280*) showing the highest fold increase in GFP abundance after deleting poly(A) were constructed on a multiple-copy vector to drive the secretory expression of AnFaeA. As shown in Fig. 4G, the deletions of poly(A)s in the 5´ UTRs of *SSH4*, *INU1*, and *KLMA_80280* significantly improved the production of AnFaeA compared to the wild-type 5´ UTRs. The results indicate that deletions of the 5´ UTR poly(A)s upstream of 30 nt preceding AUG tend to improve protein production in different ORF contexts.

A previous study showed that the presence of poly(A) in the 5´ UTR decreased mRNA levels [57]. However, we found that deletions of poly(A)s showed inconsistent effects on mRNA levels. Among the 7 mutants that caused an increase in GFP abundance, only the Δpoly(A) mutant of *KLMA_80280* 5´ UTR significantly increased the *GFP* mRNA level, while the mRNA levels expressed by the other Δpoly(A) mutants were not significantly different from those expressed by the wild-type 5´ UTRs (Fig. 4H). Among the three mutants that caused an increase in AnFaeA abundance, the Δpoly(A) mutant of *INU1* 5´ UTR increased the *AnFaeA* mRNA level, while the Δpoly(A) mutant of *KLMA_80280* 5´ UTR decreased the mRNA level (Fig. 4I). Therefore, the increased protein abundance observed in the 5´ UTR Δpoly(A) mutants was not solely due to increased mRNA levels. The results suggest that poly(A) represses protein production, either with or without reducing mRNA levels.

## Discussion

In prior studies, researchers synthesized randomly designed short 5´ UTRs (less than 100 nt) to build libraries and determined the impact of each 5´ UTR on reporter protein abundance [6, 7, 12, 25, 27]. A parallel assay was commonly performed during this step, wherein cells containing different 5´ UTRs were grown and sequenced together [6, 7, 12, 27]. Subsequently, predictive models were constructed by incorporating the features of the 5´ UTR [12, 25, 26]. In our study, we followed a similar strategy with some modifications. Firstly, we synthesized a library of natural 5´ UTRs with lengths ranging from 12 to 197 nt. Despite longer sequences increasing the complexity of machine learning, analyzing native sequences provides valuable information about natural regulation. Secondly, we evaluated each 5´ UTR’s impact on protein production separately in vivo, reducing potential interference from other cells during parallel reporter assays. Lastly, we incorporated the length and position of poly(A) into machine learning, which had not been done before. Our model accurately predicted protein production induced by the 5´ UTR (R^2^ = 0.7595), suggesting that poly(A) features are effective in constructing high-quality models. To further enhance the model’s performance, the dataset of 5´ UTRs needs to be expanded to augment the diversity of selected features, encompassing poly(A) features. Constructing a larger library comprising natural 5´ UTRs from *K. marxianus* using high-throughput synthesis techniques is a potential avenue [58, 59]. However, this task is challenging considering that the reported libraries typically consisted of 5´ UTRs with lengths of 100 nt or less [6, 7, 12, 25, 27]. Moreover, it’s crucial to introduce parallel assays to measure the impacts of 5´ UTRs on protein expression within a larger library.

The SHAP sensitivity analysis based on the MLP-NN model reveals a general negative correlation between poly(A) length and protein production, suggesting that poly(A) primarily functions as a negative regulator of protein production. Deletions of poly(A)s showed inconsistent effects on mRNA levels (Fig. 4H, I), suggesting that poly(A) represses protein production either with or without reducing mRNA levels. Consistent with this hypothesis, it was shown that the 5´ UTR poly(A) of *PABP1* can independently repress mRNA translation and reduce mRNA abundance [57]. To repress translation, the 5´ UTR poly(A) recruits PABP1 and prevents the 40S ribosomal subunit from moving to the initiation codon [22]. A similar mechanism may be employed by the 5´ UTR poly(A) in *K. marxianus*. Meanwhile, 5´ UTR poly(A) may reduce mRNA levels by decreasing its stability. The negative effect of poly(A) on mRNA stability is likely proportional to its length, since longer 5´ UTR poly(A) sequences were found to result in shorter mRNA half-lives in vitro [21]. This may partially explain the negative correlation between poly(A) length and protein production. The Ccr4–Not and Pan2–Pan3 complexes, which are responsible for the 3´-end poly(A) tail shortening, have been proposed to mediate the degradation of mRNAs containing 5´ UTR poly(A) [21]. These complexes are conserved from yeast to humans [60, 61], suggesting that they likely play similar roles in degrading 5´ UTR poly(A) sequences in *K. marxianus*.

It is noteworthy that the Δpoly(A) mutant of *KLMA_80280* 5´ UTR reduced the *AnFaeA* mRNA level (Fig. 4I). The poly(A) in *KLMA_80280* 5´ UTR is precisely located at the 5´ end of the mRNA, and the sequence around the transcriptional start site (CCA[+ 1]AAAA) matches the consensus sequence of the transcriptional initiator in *Schizosaccharomyces pombe* ((C/T)(C/T)(A/G)[+ 1]N(A/C)(A/C)) [62], where (A/G)[+ 1] represents the transcription start site. The transcriptional initiator is a core promoter element found near the transcription start site on the DNA, playing a role in directing transcription initiation [62]. Therefore, the deletion of the poly(A) tract might impair the transcription of *KLMA_80280* 5´ UTR, leading to decreased *AnFaeA* mRNA level. In contrast to the decrease in *AnFaeA* mRNA level, the ∆poly(A) mutant of *KLMA_80280* 5´ UTR slightly increased the *GFP* mRNA level (Fig. 4H). Similar inconsistent effects on mRNA levels were observed in the Δpoly(A) mutant of *INU1* 5´ UTR, which caused a slight increase in the *AnFaeA* mRNA level but no alteration in the *GFP* mRNA level (Fig. 4H, I). These inconsistent effects of poly(A) deletion on mRNA levels might be attributed to the distinct ORFs and 3´ UTRs present in the *GFP* and *AnFaeA* expression cassettes. As integral components of mature mRNA, the 5´ UTR, ORF, and 3´ UTR determine the translation process and secondary structure of mRNA, directly influencing mRNA decay and stability [63–68]. Hence, in different sequence contexts, deletions of 5´ UTR poly(A) might have varying effects on mRNA stability and levels.

**Fig. 5:**
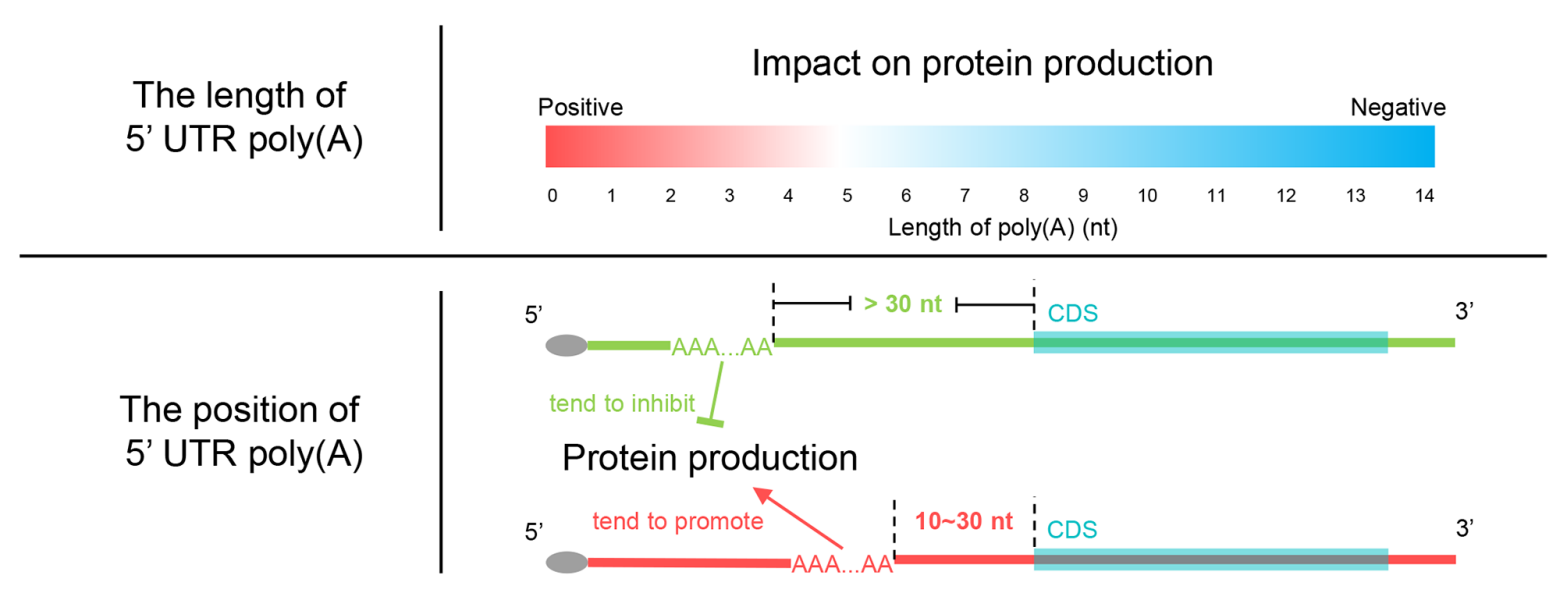
Impact of length and position of 5´ UTR poly(A) on protein production

The analysis of SHAP values also indicates a weak correlation between improved protein production and 5´ UTR poly(A) located between 10–30 nt upstream of AUG, suggesting a position-specific effect of 5´ UTR poly(A). This finding aligns with the observation that poly(A)s with a distance of 30 nt or less from AUG were enriched in genes with high translation efficiency (Fig. 1G). Poly(A) can form an IRES for cap-independent translation [17, 18, 21]. In yeast, the IRES is typically located immediately upstream of the AUG [69]. Hence, compared with poly(A) located further from the AUG, poly(A) close to the AUG has a higher probability of forming an IRES and enhancing translation. Cap-independent translation induced by nearby poly(A) may counteract the negative effect of poly(A), leading to a net positive effect on protein production. The effects of 5´ UTR poly(A) on protein production are summarized in Fig. 5.

The MLP-NN model was effectively employed to guide the optimization of natural 5´ UTR containing poly(A) (R^2^ = 0.6227). Reducing the length or removing poly(A) located upstream of 30 nt preceding AUG appeared to be an effective way to enhance protein production. This optimization strategy was successfully applied to GFP and AnFaeA (Fig. 4F, G), indicating its effectiveness across different transcriptional contexts. Traditionally, the natural promoter and 5´ UTR from the same gene were used together to drive the expression of the gene of interest. Several popular 5´ UTRs used in microbial cell factories, such as the 5´ UTRs of *TDH1* and *GAL1* in *S. cerevisiae* [70], and the 5´ UTR of *AOX1* in *Pichia pastoris* [71], contain poly(A) tracts beyond or around 30 nt preceding AUG. Manipulating poly(A)s in these 5´ UTRs might offer a new approach to enhancing yield.

## Conclusion

An MLP-NN model was trained using features encompassing 5´ UTR poly(A) length and position, which demonstrated good performance in predicting protein production. The model showed that poly(A) with a distance to AUG between 10 ~ 30 nt is slightly correlated with improved protein production. 5´ UTR poly(A) upstream of 30 nt is negatively associated with protein production. Moreover, the negative effect of poly(A) on protein production increases with tract length. With the guidance of the machine model, reducing or removing poly(A) upstream of 30 nt preceding AUG is an effective strategy for improving protein production. This approach could be applied to enhance the yield of *K. marxianus* and other microbial cell factories.

### Electronic supplementary material

Below is the link to the electronic supplementary material.

Additional file **1**: **Fig S1**: Enrichment and depletion of four bases between 100 nt and 30 nt preceding AUG (-100~-30) in different groups of genes. The genes were grouped based on the abundance of the encoded proteins (**A**, **B**) or the level of the produced mRNAs (**C**, **D**), where the top 20% (**A**, **C**) and bottom 20% (**B**, **D**) were selected to calculate the relative entropy of four bases in this region. The significance was assessed using a two-tailed Fisher’s exact test. Logos colored in red or blue represented *p* < 0.05, while gray logos represented *p* > 0.05. **Fig S2**: Comparison of protein abundance/mRNA level of different gene groups. Genes were categorized into two groups based on either the presence or absence of 5´ UTR poly(A) (**A**), or the presence or absence of 5´ UTR poly(A) with a distance of 30 nt or less from AUG (**B**). The significance was determined using a two-tailed t-test. ** *p* < 0.01. * *p* < 0.05. **Fig S3**: Validation of constructed MLP-NN models after five training-test splits using two types of feature selection. (**A**) A total of 15 features, including 5´ UTR length, poly(A) length and poly(A) position were included. A total of 5 different models were constructed using different training-test splits, and the last model was shown in Fig. 3A as a representative. The average coefficient of determination (R^2^) for predicting the test sets was 0.7290. (**B**) A total of 12 features were included, while features of 5´ UTR length, poly(A) length and poly(A) position were excluded. A total of 5 different models were constructed using different training-test splits. The average R^2^ for predicting the test sets was 0.6403. **Fig S4**: Validation of the random forest model (**A**) and the support vector machine model (**B**). The plot compared measured versus the predicted relative GFP abundance, with R^2^ for the train and test sets included. **Fig S5**: Validation of the MLP-NN model’s ability to predict protein production in *S. cerevisiae*. A 5´ UTR library consisting of half a million 50-nt sequences was constructed previously in *S. cerevisiae* [7]. The impact of each 5´ UTR on *HIS3* production was assessed by measuring the enrichment of cells harboring the 5´ UTR after cultivation in selection media. From this library, a total of 700 5´ UTRs with poly(A) and 115 5´ UTRs without poly(A) were selected. Fifteen features were extracted from the 5´ UTRs, and the MLP-NN model was employed to predict enrichments of 5´ UTRs. The predicted enrichments were compared with measured enrichments, resulting in an R^2^ of 0.503. **Fig S6**: The relationship between SHAP values and poly(A) features from models constructed using four additional training-test splits

Additional file **2**: **Table S1** List of plasmids. **Table S2** List of primers. **Table S3** Sequences of LHZ1138, LHZ1441 and LHZ1448

Additional file **3**: Sequences of 5´ UTRs

Additional file **4**: List of TPM values

Additional file **5**: List of emPAI values

Additional file **6**: Dataset for model training

## Data Availability

The datasets supporting the conclusions of this article are included within the article and its Additional files.
