# Supplementary material for: Characterization and optimization of 5´ untranslated region containing poly-adenine tracts in Kluyveromyces marxianus using machine-learning model
Source: Microb Cell Fact. 2024 Jan 3;23:7. doi: 10.1186/s12934-023-02271-3 (PMC10763412; doi:10.1186/s12934-023-02271-3)
Supplement: Supplementary file 1 — Additional file 1: Fig S1: Enrichment and depletion of four bases between 100 nt and 30 nt preceding AUG (-100~-30) in different groups of genes. The genes were grouped based on the abundance of the encoded proteins (A, B) or the level of the produced mRNAs (C, D), where the top 20% (A, C) and bottom 20% (B, D) were selected to calculate the relative entropy of four bases in this region. The significance was assessed using a two-tailed Fisher’s exact test. Logos colored in red or blue represented p < 0.05, while gray logos represented p > 0.05. Fig S2: Comparison of protein abundance/mRNA level of different gene groups. Genes were categorized into two groups based on either the presence or absence of 5´ UTR poly(A) (A), or the presence or absence of 5´ UTR poly(A) with a distance of 30 nt or less from AUG (B). The significance was determined using a two-tailed t-test. ** p < 0.01. * p < 0.05. Fig S3: Validation of constructed MLP-NN models after five training-test splits using two types of feature selection. (A) A total of 15 features, including 5´ UTR length, poly(A) length and poly(A) position were included. A total of 5 different models were constructed using different training-test splits, and the last model was shown in Fig. 3A as a representative. The average coefficient of determination (R2) for predicting the test sets was 0.7290. (B) A total of 12 features were included, while features of 5´ UTR length, poly(A) length and poly(A) position were excluded. A total of 5 different models were constructed using different training-test splits. The average R2 for predicting the test sets was 0.6403. Fig S4: Validation of the random forest model (A) and the support vector machine model (B). The plot compared measured versus the predicted relative GFP abundance, with R2 for the train and test sets included. Fig S5: Validation of the MLP-NN model’s ability to predict protein production in S. cerevisiae. A 5´ UTR library consisting of half a million 50-nt sequences was [file 12934_2023_2271_MOESM1_ESM.docx]

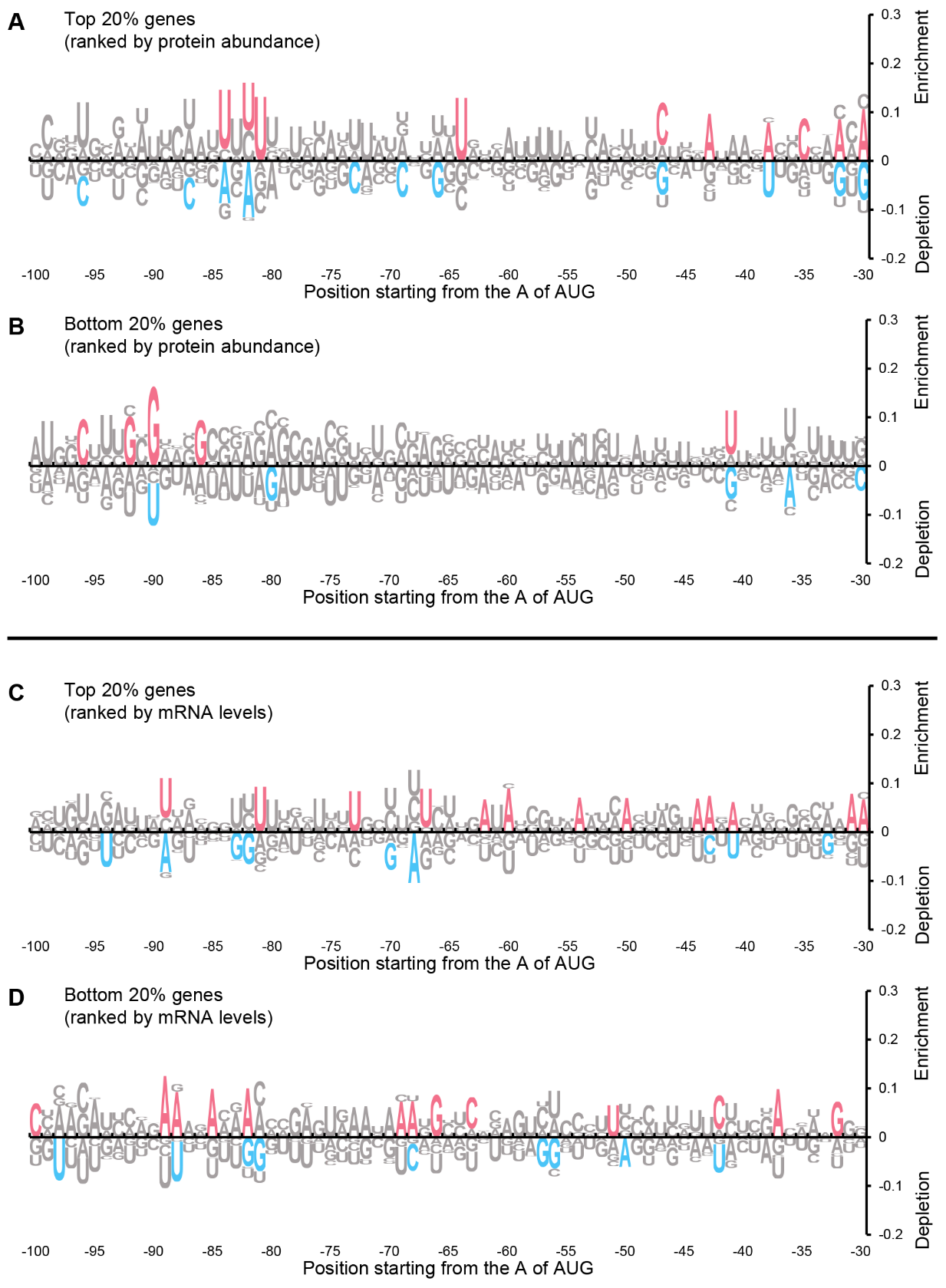


**Fig S1**: Enrichment and depletion of four bases between 100 nt and 30 nt preceding AUG (-100~-30) in different groups of genes. The genes were grouped based on the abundance of the encoded proteins (**A**, **B**) or the level of the produced mRNAs (**C**, **D**), where the top 20% (**A**, **C**) and bottom 20% (**B**, **D**) were selected to calculated the relative entropy of four bases in this region. The significance was assessed using a two-tailed Fisher’s exact test. Logos colored in red or blue represented p<0.05, while gray logos represented p>0.05.


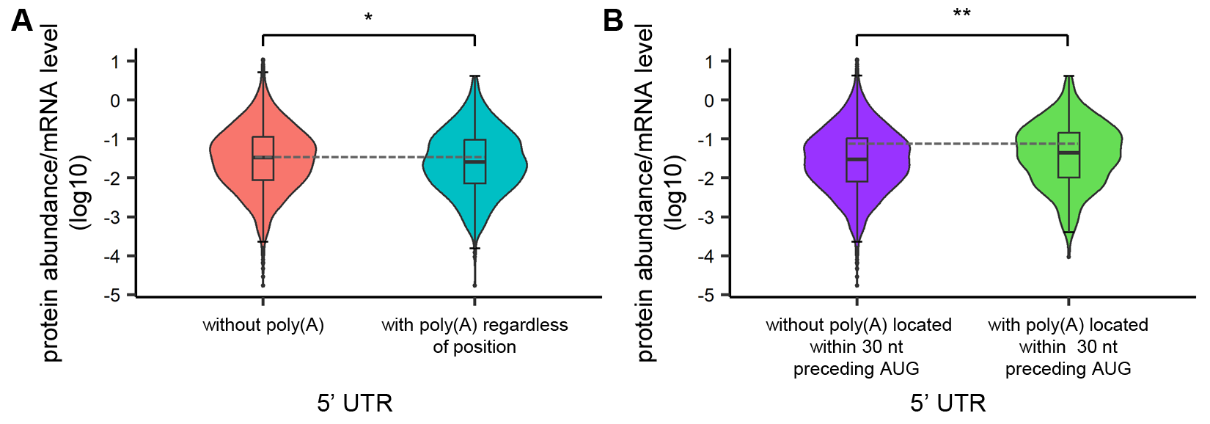


**Fig S2**: Comparison of protein abundance/mRNA level of different gene groups. Genes were categorized into two groups based on either the presence or absence of 5' UTR poly(A) (**A**), or the presence or absence of 5' UTR poly(A) with a distance of 30 nt or less from AUG (**B**). The significance was determined using a two-tailed t-test. ** p<0.01. * p<0.05.


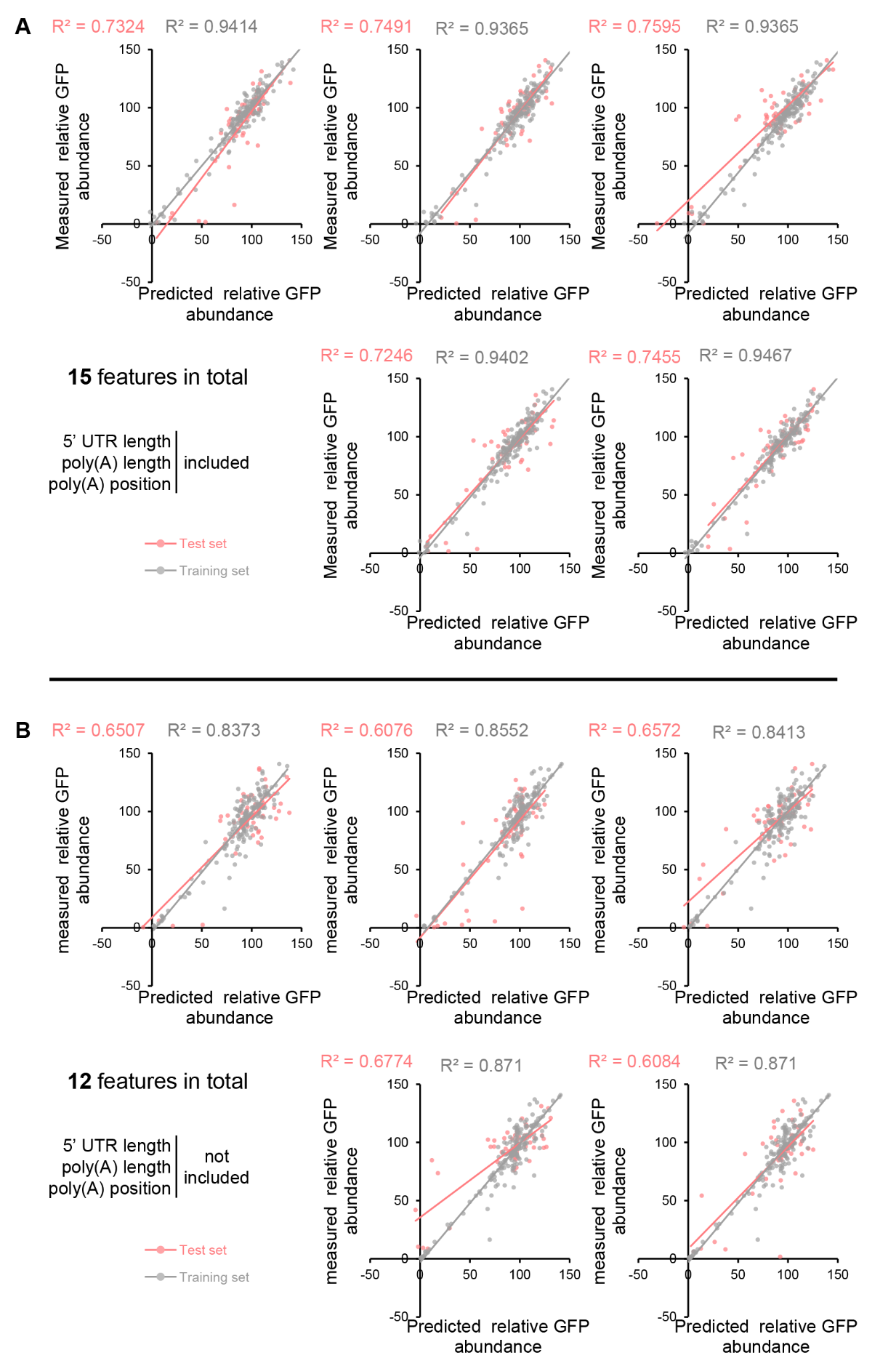


**Fig S3**: Validation of constructed MLP-NN models after five training-test splits using two types of feature selection. (**A**) A total of 15 features, including 5' UTR length, poly(A) length and poly(A) position were included. A total of 5 different models were constructed using different training-test splits, and the last model was shown in Fig 3A as a representative. The average coefficient of determination (R^2^) for predicting the test sets was 0.7290. (**B**) A total of 12 features were included, while features of 5' UTR length, poly(A) length and poly(A) position were excluded. A total of 5 different models were constructed using different training-test splits. The average R^2^ for predicting the test sets was 0.6403.


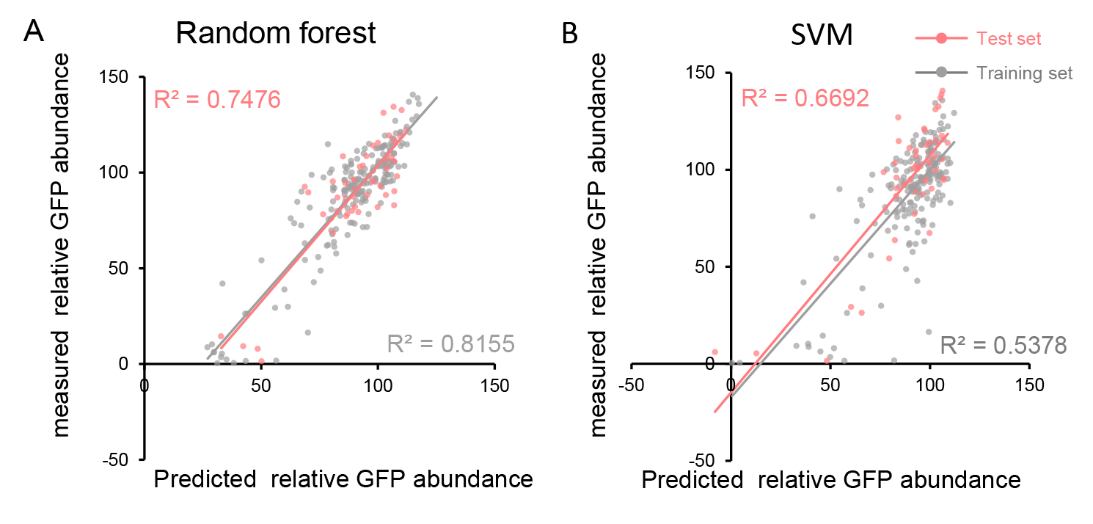


**Fig S4**: Validation of the random forest model (**A**) and the support vector machine model (**B**). The plot compared measured versus the predicted relative GFP abundance, with R^2^ for the train and test sets included.


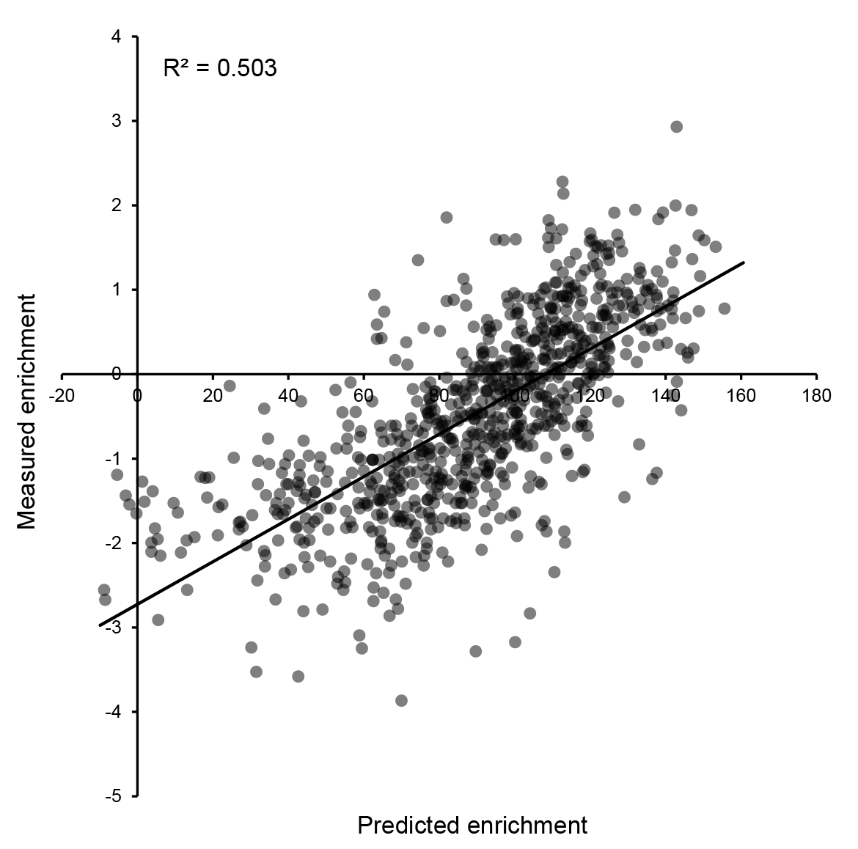


**Fig S5**: Validation of the MLP-NN model’s ability in predicting protein production in *S. cerevisiae*. A 5’ UTR library consisting of half a million 50-nt sequences was constructed previously in *S. cerevisiae* [1]. The impact of each 5’ UTR on *HIS3* production was assessed by measuring the enrichment of cells harboring the 5’ UTR after cultivation in selection media. From this library, a total of 700 5’ UTRs with poly(A) and 115 5’ UTRs without poly(A) were selected. Fifteen features were extracted from the 5' UTRs, and the MLP-NN model was employed to predict enrichments of 5’UTRs. The predicted enrichments were compared with measured enrichments, resulting in an R^2^ of 0.503.

1. Cuperus JT, Groves B, Kuchina A, Rosenberg AB, Jojic N, Fields S, Seelig G. Deep learning of the regulatory grammar of yeast 5' untranslated regions from 500,000 random sequences. Genome Res. 2017,27(12):2015-24. <https://doi.org/10.1101/gr.224964.117>


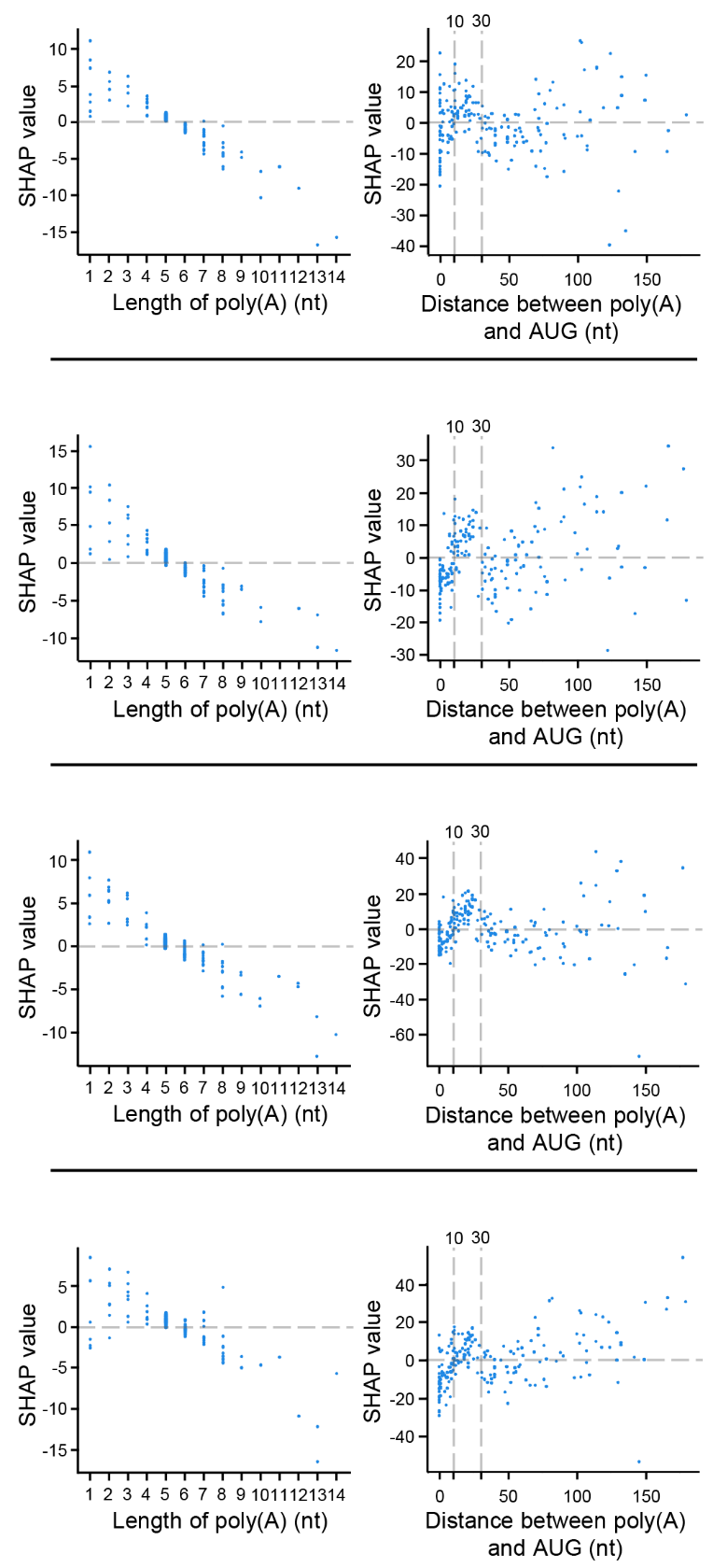


**Fig S6**: The relationship between SHAP values and poly(A) features from models constructed using four additional training-test splits.
