# Supplementary material for: Characterization and optimization of 5´ untranslated region containing poly-adenine tracts in Kluyveromyces marxianus using machine-learning model
Source: Microb Cell Fact. 2024 Jan 3;23:7. doi: 10.1186/s12934-023-02271-3 (PMC10763412; doi:10.1186/s12934-023-02271-3)
Supplement: Supplementary file 2 — Additional file 2: Table S1 List of plasmids. Table S2 List of primers. Table S3 Sequences of LHZ1138, LHZ1441 and LHZ1448 [file 12934_2023_2271_MOESM2_ESM.docx]

# Table S1 List of plasmids

| Name | Essential Features | Backbone | Application | Source or reference |
| --- | --- | --- | --- | --- |
| LHZ676 | ARS1, *AgTEF* promoter-*mCherry*-*AgTEF* terminator, *GFP*-*ScADH1* terminator, *KmURA3*^<1>^ | pMD18-T | / | <2> |
| LHZ1137 | ARS1, *AgTEF* promoter-*mCherry*-*AgTEF* terminator, *HXT4* promoter&5’ UTR-*GFP*-*ScADH1* terminator, *KmURA3* | LHZ676 | / | this study |
| LHZ1138 | ARS1, *AgTEF* promoter-*mCherry*-*AgTEF* terminator, *HXT4* promoter-*Xho* I-*GFP*-*ScADH1* terminator, *KmURA3* | LHZ1137 | Backbone vector for measuring GFP abundance caused by various 5’ UTR | this study |
| LHZ1139 | *SPP381* 5’ UTR was introduced into the *Xho* I site of LHZ1138 | LHZ1138 | Measuring *SPP381* 5’ UTR | this study |
| LHZ1140 | *GUK1* 5’ UTR was introduced into the *Xho* I site of LHZ1138 | LHZ1138 | Measuring *GUK1* 5’ UTR | this study |
| LHZ1141 | *SRX1* 5’ UTR was introduced into the *Xho* I site of LHZ1138 | LHZ1138 | Measuring *SRX1* 5’ UTR | this study |
| LHZ1142 | *SEC53* 5’ UTR was introduced into the *Xho* I site of LHZ1138 | LHZ1138 | Measuring *SEC53* 5’ UTR | this study |
| LHZ1143 | *OST1* 5’ UTR was introduced into the *Xho* I site of LHZ1138 | LHZ1138 | Measuring *OST1* 5’ UTR | this study |
| LHZ1144 | *KLMA*_*80108* 5’ UTR was introduced into the *Xho* I site of LHZ1138 | LHZ1138 | Measuring *KLMA*_*80108* 5’ UTR | this study |
| LHZ1145 | *RPL5* 5’ UTR was introduced into the *Xho* I site of LHZ1138 | LHZ1138 | Measuring *RPL5* 5’ UTR | this study |
| LHZ1146 | *AIM2* 5’ UTR was introduced into the *Xho* I site of LHZ1138 | LHZ1138 | Measuring *AIM2* 5’ UTR | this study |
| LHZ1147 | *KLMA*_*60307* 5’ UTR was introduced into the *Xho* I site of LHZ1138 | LHZ1138 | Measuring *KLMA*_*60307* 5’ UTR | this study |
| LHZ1148 | *GLO4* 5’ UTR was introduced into the *Xho* I site of LHZ1138 | LHZ1138 | Measuring *GLO4* 5’ UTR | this study |
| LHZ1149 | *FIM1*_*4812* 5’ UTR was introduced into the *Xho* I site of LHZ1138 | LHZ1138 | Measuring *FIM1*_*4812* 5’ UTR | this study |
| LHZ1150 | *MAK10* 5’ UTR was introduced into the *Xho* I site of LHZ1138 | LHZ1138 | Measuring *MAK10* 5’ UTR | this study |
| LHZ1151 | *RTC4* 5’ UTR was introduced into the *Xho* I site of LHZ1138 | LHZ1138 | Measuring *RTC4* 5’ UTR | this study |
| LHZ1152 | *KLMA*_*20131* 5’ UTR was introduced into the *Xho* I site of LHZ1138 | LHZ1138 | Measuring *KLMA*_*20131* 5’ UTR | this study |
| LHZ1153 | *KLMA*_*70012* 5’ UTR was introduced into the *Xho* I site of LHZ1138 | LHZ1138 | Measuring *KLMA*_*70012* 5’ UTR | this study |
| LHZ1154 | *CAR2* 5’ UTR was introduced into the *Xho* I site of LHZ1138 | LHZ1138 | Measuring *CAR2* 5’ UTR | this study |
| LHZ1155 | *LAS17* 5’ UTR was introduced into the *Xho* I site of LHZ1138 | LHZ1138 | Measuring *LAS17* 5’ UTR | this study |
| LHZ1156 | *SVF1* 5’ UTR was introduced into the *Xho* I site of LHZ1138 | LHZ1138 | Measuring *SVF1* 5’ UTR | this study |
| LHZ1157 | *SIW14* 5’ UTR was introduced into the *Xho* I site of LHZ1138 | LHZ1138 | Measuring *SIW14* 5’ UTR | this study |
| LHZ1158 | *KLMA*_*80280* 5’ UTR was introduced into the *Xho* I site of LHZ1138 | LHZ1138 | Measuring *KLMA*_*80280* 5’ UTR | this study |
| LHZ1159 | *LAC4* 5’ UTR was introduced into the *Xho* I site of LHZ1138 | LHZ1138 | Measuring *LAC4* 5’ UTR | this study |
| LHZ1160 | *ZPR1* 5’ UTR was introduced into the *Xho* I site of LHZ1138 | LHZ1138 | Measuring *ZPR1* 5’ UTR | this study |
| LHZ1161 | *GPT2* 5’ UTR was introduced into the *Xho* I site of LHZ1138 | LHZ1138 | Measuring *GPT2* 5’ UTR | this study |
| LHZ1162 | *KLMA*_*50514* 5’ UTR was introduced into the *Xho* I site of LHZ1138 | LHZ1138 | Measuring *KLMA*_*50514* 5’ UTR | this study |
| LHZ1163 | *SNO3* 5’ UTR was introduced into the *Xho* I site of LHZ1138 | LHZ1138 | Measuring *SNO3* 5’ UTR | this study |
| LHZ1164 | *INU1* 5’ UTR was introduced into the *Xho* I site of LHZ1138 | LHZ1138 | Measuring *INU1* 5’ UTR | this study |
| LHZ1165 | *FIM1*_*2796* 5’ UTR was introduced into the *Xho* I site of LHZ1138 | LHZ1138 | Measuring *FIM1*_*2796* 5’ UTR | this study |
| LHZ1166 | *ARC40* 5’ UTR was introduced into the *Xho* I site of LHZ1138 | LHZ1138 | Measuring *ARC40* 5’ UTR | this study |
| LHZ1167 | *SKN7* 5’ UTR was introduced into the *Xho* I site of LHZ1138 | LHZ1138 | Measuring *SKN7* 5’ UTR | this study |
| LHZ1168 | *CPS1* 5’ UTR was introduced into the *Xho* I site of LHZ1138 | LHZ1138 | Measuring *CPS1* 5’ UTR | this study |
| LHZ1169 | *ILV1* 5’ UTR was introduced into the *Xho* I site of LHZ1138 | LHZ1138 | Measuring *ILV1* 5’ UTR | this study |
| LHZ1170 | *DBP5* 5’ UTR was introduced into the *Xho* I site of LHZ1138 | LHZ1138 | Measuring *DBP5* 5’ UTR | this study |
| LHZ1171 | *NDI1* 5’ UTR was introduced into the *Xho* I site of LHZ1138 | LHZ1138 | Measuring *NDI1* 5’ UTR | this study |
| LHZ1172 | *SEC62* 5’ UTR was introduced into the *Xho* I site of LHZ1138 | LHZ1138 | Measuring *SEC62* 5’ UTR | this study |
| LHZ1173 | *FRS1* 5’ UTR was introduced into the *Xho* I site of LHZ1138 | LHZ1138 | Measuring *FRS1* 5’ UTR | this study |
| LHZ1174 | *TNA1* 5’ UTR was introduced into the *Xho* I site of LHZ1138 | LHZ1138 | Measuring *TNA1* 5’ UTR | this study |
| LHZ1175 | *NUP170* 5’ UTR was introduced into the *Xho* I site of LHZ1138 | LHZ1138 | Measuring *NUP170* 5’ UTR | this study |
| LHZ1176 | *rhb1* 5’ UTR was introduced into the *Xho* I site of LHZ1138 | LHZ1138 | Measuring *rhb1* 5’ UTR | this study |
| LHZ1177 | *NCE103* 5’ UTR was introduced into the *Xho* I site of LHZ1138 | LHZ1138 | Measuring *NCE103* 5’ UTR | this study |
| LHZ1178 | *ARC18* 5’ UTR was introduced into the *Xho* I site of LHZ1138 | LHZ1138 | Measuring *ARC18* 5’ UTR | this study |
| LHZ1179 | *PEX25* 5’ UTR was introduced into the *Xho* I site of LHZ1138 | LHZ1138 | Measuring *PEX25* 5’ UTR | this study |
| LHZ1180 | *SNU13* 5’ UTR was introduced into the *Xho* I site of LHZ1138 | LHZ1138 | Measuring *SNU13* 5’ UTR | this study |
| LHZ1181 | *KLMA*_*80013* 5’ UTR was introduced into the *Xho* I site of LHZ1138 | LHZ1138 | Measuring *KLMA*_*80013* 5’ UTR | this study |
| LHZ1182 | *SSH4* 5’ UTR was introduced into the *Xho* I site of LHZ1138 | LHZ1138 | Measuring *SSH4* 5’ UTR | this study |
| LHZ1183 | *NEW1* 5’ UTR was introduced into the *Xho* I site of LHZ1138 | LHZ1138 | Measuring *NEW1* 5’ UTR | this study |
| LHZ1184 | *COP1* 5’ UTR was introduced into the *Xho* I site of LHZ1138 | LHZ1138 | Measuring *COP1* 5’ UTR | this study |
| LHZ1185 | *GIM4* 5’ UTR was introduced into the *Xho* I site of LHZ1138 | LHZ1138 | Measuring *GIM4* 5’ UTR | this study |
| LHZ1186 | *CDC10* 5’ UTR was introduced into the *Xho* I site of LHZ1138 | LHZ1138 | Measuring *CDC10* 5’ UTR | this study |
| LHZ1187 | *CDC12* 5’ UTR was introduced into the *Xho* I site of LHZ1138 | LHZ1138 | Measuring *CDC12* 5’ UTR | this study |
| LHZ1188 | *LSM1* 5’ UTR was introduced into the *Xho* I site of LHZ1138 | LHZ1138 | Measuring *LSM1* 5’ UTR | this study |
| LHZ1189 | *DRE2* 5’ UTR was introduced into the *Xho* I site of LHZ1138 | LHZ1138 | Measuring *DRE2* 5’ UTR | this study |
| LHZ1190 | *YTA12* 5’ UTR was introduced into the *Xho* I site of LHZ1138 | LHZ1138 | Measuring *YTA12* 5’ UTR | this study |
| LHZ1191 | *QRI1* 5’ UTR was introduced into the *Xho* I site of LHZ1138 | LHZ1138 | Measuring *QRI1* 5’ UTR | this study |
| LHZ1192 | *SKG1* 5’ UTR was introduced into the *Xho* I site of LHZ1138 | LHZ1138 | Measuring *SKG1* 5’ UTR | this study |
| LHZ1193 | *ACH1* 5’ UTR was introduced into the *Xho* I site of LHZ1138 | LHZ1138 | Measuring *ACH1* 5’ UTR | this study |
| LHZ1194 | *PET8* 5’ UTR was introduced into the *Xho* I site of LHZ1138 | LHZ1138 | Measuring *PET8* 5’ UTR | this study |
| LHZ1195 | *TCM62* 5’ UTR was introduced into the *Xho* I site of LHZ1138 | LHZ1138 | Measuring *TCM62* 5’ UTR | this study |
| LHZ1196 | *PTK2* 5’ UTR was introduced into the *Xho* I site of LHZ1138 | LHZ1138 | Measuring *PTK2* 5’ UTR | this study |
| LHZ1197 | *USA1* 5’ UTR was introduced into the *Xho* I site of LHZ1138 | LHZ1138 | Measuring *USA1* 5’ UTR | this study |
| LHZ1198 | *SSE1* 5’ UTR was introduced into the *Xho* I site of LHZ1138 | LHZ1138 | Measuring *SSE1* 5’ UTR | this study |
| LHZ1199 | *ECM14* 5’ UTR was introduced into the *Xho* I site of LHZ1138 | LHZ1138 | Measuring *ECM14* 5’ UTR | this study |
| LHZ1200 | *CYS3* 5’ UTR was introduced into the *Xho* I site of LHZ1138 | LHZ1138 | Measuring *CYS3* 5’ UTR | this study |
| LHZ1201 | *ISD11* 5’ UTR was introduced into the *Xho* I site of LHZ1138 | LHZ1138 | Measuring *ISD11* 5’ UTR | this study |
| LHZ1202 | *MNP1* 5’ UTR was introduced into the *Xho* I site of LHZ1138 | LHZ1138 | Measuring *MNP1* 5’ UTR | this study |
| LHZ1203 | *HIS2* 5’ UTR was introduced into the *Xho* I site of LHZ1138 | LHZ1138 | Measuring *HIS2* 5’ UTR | this study |
| LHZ1204 | *RPT6* 5’ UTR was introduced into the *Xho* I site of LHZ1138 | LHZ1138 | Measuring *RPT6* 5’ UTR | this study |
| LHZ1205 | *UTP15* 5’ UTR was introduced into the *Xho* I site of LHZ1138 | LHZ1138 | Measuring *UTP15* 5’ UTR | this study |
| LHZ1206 | *COX16* 5’ UTR was introduced into the *Xho* I site of LHZ1138 | LHZ1138 | Measuring *COX16* 5’ UTR | this study |
| LHZ1207 | *MNN10* 5’ UTR was introduced into the *Xho* I site of LHZ1138 | LHZ1138 | Measuring *MNN10* 5’ UTR | this study |
| LHZ1208 | *SSB* 5’ UTR was introduced into the *Xho* I site of LHZ1138 | LHZ1138 | Measuring *SSB* 5’ UTR | this study |
| LHZ1209 | *OLE1* 5’ UTR was introduced into the *Xho* I site of LHZ1138 | LHZ1138 | Measuring *OLE1* 5’ UTR | this study |
| LHZ1210 | *RLI1* 5’ UTR was introduced into the *Xho* I site of LHZ1138 | LHZ1138 | Measuring *RLI1* 5’ UTR | this study |
| LHZ1211 | *PSY2* 5’ UTR was introduced into the *Xho* I site of LHZ1138 | LHZ1138 | Measuring *PSY2* 5’ UTR | this study |
| LHZ1212 | *KLMA*_*20249* 5’ UTR was introduced into the *Xho* I site of LHZ1138 | LHZ1138 | Measuring *KLMA*_*20249* 5’ UTR | this study |
| LHZ1213 | *GAL1* 5’ UTR was introduced into the *Xho* I site of LHZ1138 | LHZ1138 | Measuring *GAL1* 5’ UTR | this study |
| LHZ1214 | *RSM24* 5’ UTR was introduced into the *Xho* I site of LHZ1138 | LHZ1138 | Measuring *RSM24* 5’ UTR | this study |
| LHZ1215 | *FRS2* 5’ UTR was introduced into the *Xho* I site of LHZ1138 | LHZ1138 | Measuring *FRS2* 5’ UTR | this study |
| LHZ1216 | *AYR1* 5’ UTR was introduced into the *Xho* I site of LHZ1138 | LHZ1138 | Measuring *AYR1* 5’ UTR | this study |
| LHZ1217 | *LSB6* 5’ UTR was introduced into the *Xho* I site of LHZ1138 | LHZ1138 | Measuring *LSB6* 5’ UTR | this study |
| LHZ1218 | *SNF1* 5’ UTR was introduced into the *Xho* I site of LHZ1138 | LHZ1138 | Measuring *SNF1* 5’ UTR | this study |
| LHZ1219 | *rplM* 5’ UTR was introduced into the *Xho* I site of LHZ1138 | LHZ1138 | Measuring *rplM* 5’ UTR | this study |
| LHZ1220 | *SSA2* 5’ UTR was introduced into the *Xho* I site of LHZ1138 | LHZ1138 | Measuring *SSA2* 5’ UTR | this study |
| LHZ1221 | *GCD7* 5’ UTR was introduced into the *Xho* I site of LHZ1138 | LHZ1138 | Measuring *GCD7* 5’ UTR | this study |
| LHZ1222 | *PHB2* 5’ UTR was introduced into the *Xho* I site of LHZ1138 | LHZ1138 | Measuring *PHB2* 5’ UTR | this study |
| LHZ1223 | *YHM2* 5’ UTR was introduced into the *Xho* I site of LHZ1138 | LHZ1138 | Measuring *YHM2* 5’ UTR | this study |
| LHZ1224 | *STR3* 5’ UTR was introduced into the *Xho* I site of LHZ1138 | LHZ1138 | Measuring *STR3* 5’ UTR | this study |
| LHZ1225 | *SAC1* 5’ UTR was introduced into the *Xho* I site of LHZ1138 | LHZ1138 | Measuring *SAC1* 5’ UTR | this study |
| LHZ1226 | *OXA1* 5’ UTR was introduced into the *Xho* I site of LHZ1138 | LHZ1138 | Measuring *OXA1* 5’ UTR | this study |
| LHZ1227 | *DBP9* 5’ UTR was introduced into the *Xho* I site of LHZ1138 | LHZ1138 | Measuring *DBP9* 5’ UTR | this study |
| LHZ1228 | *PLP2* 5’ UTR was introduced into the *Xho* I site of LHZ1138 | LHZ1138 | Measuring *PLP2* 5’ UTR | this study |
| LHZ1229 | *ATG27* 5’ UTR was introduced into the *Xho* I site of LHZ1138 | LHZ1138 | Measuring *ATG27* 5’ UTR | this study |
| LHZ1230 | mutant *GAL1* 5’ UTR (harboring a ΔAT mutation at position [-23, 22], where the A of start codon is [+1]) was introduced into the *Xho* I site of LHZ1138 | LHZ1138 | Measuring *mutant* *GAL1* 5’ UTR | this study |
| LHZ1231 | *DUG2* 5’ UTR was introduced into the *Xho* I site of LHZ1138 | LHZ1138 | Measuring *DUG2* 5’ UTR | this study |
| LHZ1232 | *FIM1*_*2079* 5’ UTR was introduced into the *Xho* I site of LHZ1138 | LHZ1138 | Measuring *FIM1*_*2079* 5’ UTR | this study |
| LHZ1233 | *PSE1* 5’ UTR was introduced into the *Xho* I site of LHZ1138 | LHZ1138 | Measuring *PSE1* 5’ UTR | this study |
| LHZ1234 | *CAJ1* 5’ UTR was introduced into the *Xho* I site of LHZ1138 | LHZ1138 | Measuring *CAJ1* 5’ UTR | this study |
| LHZ1235 | *ARO1* 5’ UTR was introduced into the *Xho* I site of LHZ1138 | LHZ1138 | Measuring *ARO1* 5’ UTR | this study |
| LHZ1236 | *AAT2* 5’ UTR was introduced into the *Xho* I site of LHZ1138 | LHZ1138 | Measuring *AAT2* 5’ UTR | this study |
| LHZ1237 | *TPA1* 5’ UTR was introduced into the *Xho* I site of LHZ1138 | LHZ1138 | Measuring *TPA1* 5’ UTR | this study |
| LHZ1238 | *PET10* 5’ UTR was introduced into the *Xho* I site of LHZ1138 | LHZ1138 | Measuring *PET10* 5’ UTR | this study |
| LHZ1239 | *ADE17* 5’ UTR was introduced into the *Xho* I site of LHZ1138 | LHZ1138 | Measuring *ADE17* 5’ UTR | this study |
| LHZ1240 | *CDC11* 5’ UTR was introduced into the *Xho* I site of LHZ1138 | LHZ1138 | Measuring *CDC11* 5’ UTR | this study |
| LHZ1241 | *TSA1* 5’ UTR was introduced into the *Xho* I site of LHZ1138 | LHZ1138 | Measuring *TSA1* 5’ UTR | this study |
| LHZ1242 | *GPX2* 5’ UTR was introduced into the *Xho* I site of LHZ1138 | LHZ1138 | Measuring *GPX2* 5’ UTR | this study |
| LHZ1243 | *YAP1* 5’ UTR was introduced into the *Xho* I site of LHZ1138 | LHZ1138 | Measuring *YAP1* 5’ UTR | this study |
| LHZ1244 | *TUP1* 5’ UTR was introduced into the *Xho* I site of LHZ1138 | LHZ1138 | Measuring *TUP1* 5’ UTR | this study |
| LHZ1245 | *YPT52* 5’ UTR was introduced into the *Xho* I site of LHZ1138 | LHZ1138 | Measuring *YPT52* 5’ UTR | this study |
| LHZ1246 | mutant *AYR1* 5’ UTR (an A→G mutation at position [-1]) was introduced into the *Xho* I site of LHZ1138 | LHZ1138 | Measuring *mutant* *AYR1* 5’ UTR | this study |
| LHZ1247 | *CCT6* 5’ UTR was introduced into the *Xho* I site of LHZ1138 | LHZ1138 | Measuring *CCT6* 5’ UTR | this study |
| LHZ1248 | *PRS3* 5’ UTR was introduced into the *Xho* I site of LHZ1138 | LHZ1138 | Measuring *PRS3* 5’ UTR | this study |
| LHZ1249 | *DPB3* 5’ UTR was introduced into the *Xho* I site of LHZ1138 | LHZ1138 | Measuring *DPB3* 5’ UTR | this study |
| LHZ1250 | *PAA1* 5’ UTR was introduced into the *Xho* I site of LHZ1138 | LHZ1138 | Measuring *PAA1* 5’ UTR | this study |
| LHZ1251 | *KLMA*_*40607* 5’ UTR was introduced into the *Xho* I site of LHZ1138 | LHZ1138 | Measuring *KLMA*_*40607* 5’ UTR | this study |
| LHZ1252 | *RPN4* 5’ UTR was introduced into the *Xho* I site of LHZ1138 | LHZ1138 | Measuring *RPN4* 5’ UTR | this study |
| LHZ1253 | *KLMA*_*70208* 5’ UTR was introduced into the *Xho* I site of LHZ1138 | LHZ1138 | Measuring *KLMA*_*70208* 5’ UTR | this study |
| LHZ1254 | *PAM16* 5’ UTR was introduced into the *Xho* I site of LHZ1138 | LHZ1138 | Measuring *PAM16* 5’ UTR | this study |
| LHZ1255 | *KLMA*_*80335* 5’ UTR was introduced into the *Xho* I site of LHZ1138 | LHZ1138 | Measuring *KLMA*_*80335* 5’ UTR | this study |
| LHZ1256 | *RTN1* 5’ UTR was introduced into the *Xho* I site of LHZ1138 | LHZ1138 | Measuring *RTN1* 5’ UTR | this study |
| LHZ1257 | *rplF* 5’ UTR was introduced into the *Xho* I site of LHZ1138 | LHZ1138 | Measuring *rplF* 5’ UTR | this study |
| LHZ1258 | *KLMA*_*60154* 5’ UTR was introduced into the *Xho* I site of LHZ1138 | LHZ1138 | Measuring *KLMA*_*60154* 5’ UTR | this study |
| LHZ1259 | *GRE2* 5’ UTR was introduced into the *Xho* I site of LHZ1138 | LHZ1138 | Measuring *GRE2* 5’ UTR | this study |
| LHZ1260 | *KLMA*_*60072* 5’ UTR was introduced into the *Xho* I site of LHZ1138 | LHZ1138 | Measuring *KLMA*_*60072* 5’ UTR | this study |
| LHZ1261 | *COX23* 5’ UTR was introduced into the *Xho* I site of LHZ1138 | LHZ1138 | Measuring *COX23* 5’ UTR | this study |
| LHZ1262 | *ATP7* 5’ UTR was introduced into the *Xho* I site of LHZ1138 | LHZ1138 | Measuring *ATP7* 5’ UTR | this study |
| LHZ1263 | *KLMA*_*70011* 5’ UTR was introduced into the *Xho* I site of LHZ1138 | LHZ1138 | Measuring *KLMA*_*70011* 5’ UTR | this study |
| LHZ1264 | *LIA1* 5’ UTR was introduced into the *Xho* I site of LHZ1138 | LHZ1138 | Measuring *LIA1* 5’ UTR | this study |
| LHZ1265 | *VMA2* 5’ UTR was introduced into the *Xho* I site of LHZ1138 | LHZ1138 | Measuring *VMA2* 5’ UTR | this study |
| LHZ1266 | *TRL1* 5’ UTR was introduced into the *Xho* I site of LHZ1138 | LHZ1138 | Measuring *TRL1* 5’ UTR | this study |
| LHZ1267 | *PHM7* 5’ UTR was introduced into the *Xho* I site of LHZ1138 | LHZ1138 | Measuring *PHM7* 5’ UTR | this study |
| LHZ1268 | *VPS74* 5’ UTR was introduced into the *Xho* I site of LHZ1138 | LHZ1138 | Measuring *VPS74* 5’ UTR | this study |
| LHZ1269 | *GCD11* 5’ UTR was introduced into the *Xho* I site of LHZ1138 | LHZ1138 | Measuring *GCD11* 5’ UTR | this study |
| LHZ1270 | *MTR2* 5’ UTR was introduced into the *Xho* I site of LHZ1138 | LHZ1138 | Measuring *MTR2* 5’ UTR | this study |
| LHZ1271 | *RPB11* 5’ UTR was introduced into the *Xho* I site of LHZ1138 | LHZ1138 | Measuring *RPB11* 5’ UTR | this study |
| LHZ1272 | *HSP12* 5’ UTR was introduced into the *Xho* I site of LHZ1138 | LHZ1138 | Measuring *HSP12* 5’ UTR | this study |
| LHZ1273 | *GUA1* 5’ UTR was introduced into the *Xho* I site of LHZ1138 | LHZ1138 | Measuring *GUA1* 5’ UTR | this study |
| LHZ1274 | *FPR3* 5’ UTR was introduced into the *Xho* I site of LHZ1138 | LHZ1138 | Measuring *FPR3* 5’ UTR | this study |
| LHZ1275 | *ERV25* 5’ UTR was introduced into the *Xho* I site of LHZ1138 | LHZ1138 | Measuring *ERV25* 5’ UTR | this study |
| LHZ1276 | *RPC82* 5’ UTR was introduced into the *Xho* I site of LHZ1138 | LHZ1138 | Measuring *RPC82* 5’ UTR | this study |
| LHZ1277 | *DPM1* 5’ UTR was introduced into the *Xho* I site of LHZ1138 | LHZ1138 | Measuring *DPM1* 5’ UTR | this study |
| LHZ1278 | *CLC1* 5’ UTR was introduced into the *Xho* I site of LHZ1138 | LHZ1138 | Measuring *CLC1* 5’ UTR | this study |
| LHZ1279 | *fabG* 5’ UTR was introduced into the *Xho* I site of LHZ1138 | LHZ1138 | Measuring *fabG* 5’ UTR | this study |
| LHZ1280 | *FMP37* 5’ UTR was introduced into the *Xho* I site of LHZ1138 | LHZ1138 | Measuring *FMP37* 5’ UTR | this study |
| LHZ1281 | *COR1* 5’ UTR was introduced into the *Xho* I site of LHZ1138 | LHZ1138 | Measuring *COR1* 5’ UTR | this study |
| LHZ1282 | *TMA22* 5’ UTR was introduced into the *Xho* I site of LHZ1138 | LHZ1138 | Measuring *TMA22* 5’ UTR | this study |
| LHZ1283 | *CMD1* 5’ UTR was introduced into the *Xho* I site of LHZ1138 | LHZ1138 | Measuring *CMD1* 5’ UTR | this study |
| LHZ1284 | *SXM1* 5’ UTR was introduced into the *Xho* I site of LHZ1138 | LHZ1138 | Measuring *SXM1* 5’ UTR | this study |
| LHZ1285 | *COX5A* 5’ UTR was introduced into the *Xho* I site of LHZ1138 | LHZ1138 | Measuring *COX5A* 5’ UTR | this study |
| LHZ1286 | *LAT1* 5’ UTR was introduced into the *Xho* I site of LHZ1138 | LHZ1138 | Measuring *LAT1* 5’ UTR | this study |
| LHZ1287 | *ATP1* 5’ UTR was introduced into the *Xho* I site of LHZ1138 | LHZ1138 | Measuring *ATP1* 5’ UTR | this study |
| LHZ1288 | *RIB7* 5’ UTR was introduced into the *Xho* I site of LHZ1138 | LHZ1138 | Measuring *RIB7* 5’ UTR | this study |
| LHZ1289 | *PDI1* 5’ UTR was introduced into the *Xho* I site of LHZ1138 | LHZ1138 | Measuring *PDI1* 5’ UTR | this study |
| LHZ1290 | *YKT6* 5’ UTR was introduced into the *Xho* I site of LHZ1138 | LHZ1138 | Measuring *YKT6* 5’ UTR | this study |
| LHZ1291 | *GLC3* 5’ UTR was introduced into the *Xho* I site of LHZ1138 | LHZ1138 | Measuring *GLC3* 5’ UTR | this study |
| LHZ1292 | *RAG5* 5’ UTR was introduced into the *Xho* I site of LHZ1138 | LHZ1138 | Measuring *RAG5* 5’ UTR | this study |
| LHZ1293 | *ERG10* 5’ UTR was introduced into the *Xho* I site of LHZ1138 | LHZ1138 | Measuring *ERG10* 5’ UTR | this study |
| LHZ1294 | *ARC19* 5’ UTR was introduced into the *Xho* I site of LHZ1138 | LHZ1138 | Measuring *ARC19* 5’ UTR | this study |
| LHZ1295 | *MOS1* 5’ UTR was introduced into the *Xho* I site of LHZ1138 | LHZ1138 | Measuring *MOS1* 5’ UTR | this study |
| LHZ1296 | *KLMA*_*50074* 5’ UTR was introduced into the *Xho* I site of LHZ1138 | LHZ1138 | Measuring *KLMA*_*50074* 5’ UTR | this study |
| LHZ1297 | *ATG12* 5’ UTR was introduced into the *Xho* I site of LHZ1138 | LHZ1138 | Measuring *ATG12* 5’ UTR | this study |
| LHZ1298 | *THR4* 5’ UTR was introduced into the *Xho* I site of LHZ1138 | LHZ1138 | Measuring *THR4* 5’ UTR | this study |
| LHZ1299 | *BFR1* 5’ UTR was introduced into the *Xho* I site of LHZ1138 | LHZ1138 | Measuring *BFR1* 5’ UTR | this study |
| LHZ1300 | *PEP4* 5’ UTR was introduced into the *Xho* I site of LHZ1138 | LHZ1138 | Measuring *PEP4* 5’ UTR | this study |
| LHZ1301 | *PRO1* 5’ UTR was introduced into the *Xho* I site of LHZ1138 | LHZ1138 | Measuring *PRO1* 5’ UTR | this study |
| LHZ1302 | *HYP2* 5’ UTR was introduced into the *Xho* I site of LHZ1138 | LHZ1138 | Measuring *HYP2* 5’ UTR | this study |
| LHZ1303 | *ADY2* 5’ UTR was introduced into the *Xho* I site of LHZ1138 | LHZ1138 | Measuring *ADY2* 5’ UTR | this study |
| LHZ1304 | *KLMA*_*30365* 5’ UTR was introduced into the *Xho* I site of LHZ1138 | LHZ1138 | Measuring *KLMA*_*30365* 5’ UTR | this study |
| LHZ1305 | *IDP1* 5’ UTR was introduced into the *Xho* I site of LHZ1138 | LHZ1138 | Measuring *IDP1* 5’ UTR | this study |
| LHZ1306 | *GNT1*-*B* 5’ UTR was introduced into the *Xho* I site of LHZ1138 | LHZ1138 | Measuring *GNT1*-*B* 5’ UTR | this study |
| LHZ1307 | *AIP1* 5’ UTR was introduced into the *Xho* I site of LHZ1138 | LHZ1138 | Measuring *AIP1* 5’ UTR | this study |
| LHZ1308 | *LEU4* 5’ UTR was introduced into the *Xho* I site of LHZ1138 | LHZ1138 | Measuring *LEU4* 5’ UTR | this study |
| LHZ1309 | *VMA10* 5’ UTR was introduced into the *Xho* I site of LHZ1138 | LHZ1138 | Measuring *VMA10* 5’ UTR | this study |
| LHZ1310 | *RIP1* 5’ UTR was introduced into the *Xho* I site of LHZ1138 | LHZ1138 | Measuring *RIP1* 5’ UTR | this study |
| LHZ1311 | *RPT3* 5’ UTR was introduced into the *Xho* I site of LHZ1138 | LHZ1138 | Measuring *RPT3* 5’ UTR | this study |
| LHZ1312 | *PYK1* 5’ UTR was introduced into the *Xho* I site of LHZ1138 | LHZ1138 | Measuring *PYK1* 5’ UTR | this study |
| LHZ1313 | *YVH1* 5’ UTR was introduced into the *Xho* I site of LHZ1138 | LHZ1138 | Measuring *YVH1* 5’ UTR | this study |
| LHZ1314 | *KLMA*_*40364* 5’ UTR was introduced into the *Xho* I site of LHZ1138 | LHZ1138 | Measuring *KLMA*_*40364* 5’ UTR | this study |
| LHZ1315 | *HGH1* 5’ UTR was introduced into the *Xho* I site of LHZ1138 | LHZ1138 | Measuring *HGH1* 5’ UTR | this study |
| LHZ1316 | *STE50* 5’ UTR was introduced into the *Xho* I site of LHZ1138 | LHZ1138 | Measuring *STE50* 5’ UTR | this study |
| LHZ1317 | *KLMA*_*50171* 5’ UTR was introduced into the *Xho* I site of LHZ1138 | LHZ1138 | Measuring *KLMA*_*50171* 5’ UTR | this study |
| LHZ1318 | *FIM1*_*2625* 5’ UTR was introduced into the *Xho* I site of LHZ1138 | LHZ1138 | Measuring *FIM1*_*2625* 5’ UTR | this study |
| LHZ1319 | *ARB1* 5’ UTR was introduced into the *Xho* I site of LHZ1138 | LHZ1138 | Measuring *ARB1* 5’ UTR | this study |
| LHZ1320 | *SOU1* 5’ UTR was introduced into the *Xho* I site of LHZ1138 | LHZ1138 | Measuring *SOU1* 5’ UTR | this study |
| LHZ1321 | *KLMA*_*60098* 5’ UTR was introduced into the *Xho* I site of LHZ1138 | LHZ1138 | Measuring *KLMA*_*60098* 5’ UTR | this study |
| LHZ1322 | *TAL1* 5’ UTR was introduced into the *Xho* I site of LHZ1138 | LHZ1138 | Measuring *TAL1* 5’ UTR | this study |
| LHZ1323 | *KLMA*_*10828* 5’ UTR was introduced into the *Xho* I site of LHZ1138 | LHZ1138 | Measuring *KLMA*_*10828* 5’ UTR | this study |
| LHZ1324 | *KLMA*_*50448* 5’ UTR was introduced into the *Xho* I site of LHZ1138 | LHZ1138 | Measuring *KLMA*_*50448* 5’ UTR | this study |
| LHZ1325 | *DYS1* 5’ UTR was introduced into the *Xho* I site of LHZ1138 | LHZ1138 | Measuring *DYS1* 5’ UTR | this study |
| LHZ1326 | *RNA1* 5’ UTR was introduced into the *Xho* I site of LHZ1138 | LHZ1138 | Measuring *RNA1* 5’ UTR | this study |
| LHZ1327 | *MRPL33* 5’ UTR was introduced into the *Xho* I site of LHZ1138 | LHZ1138 | Measuring *MRPL33* 5’ UTR | this study |
| LHZ1328 | *SDH4* 5’ UTR was introduced into the *Xho* I site of LHZ1138 | LHZ1138 | Measuring *SDH4* 5’ UTR | this study |
| LHZ1329 | *SEC61* 5’ UTR was introduced into the *Xho* I site of LHZ1138 | LHZ1138 | Measuring *SEC61* 5’ UTR | this study |
| LHZ1330 | *GAL10* 5’ UTR was introduced into the *Xho* I site of LHZ1138 | LHZ1138 | Measuring *GAL10* 5’ UTR | this study |
| LHZ1331 | *GCD2* 5’ UTR was introduced into the *Xho* I site of LHZ1138 | LHZ1138 | Measuring *GCD2* 5’ UTR | this study |
| LHZ1332 | *GAP1* 5’ UTR was introduced into the *Xho* I site of LHZ1138 | LHZ1138 | Measuring *GAP1* 5’ UTR | this study |
| LHZ1333 | *LAC12* 5’ UTR was introduced into the *Xho* I site of LHZ1138 | LHZ1138 | Measuring *LAC12* 5’ UTR | this study |
| LHZ1334 | *SSP120* 5’ UTR was introduced into the *Xho* I site of LHZ1138 | LHZ1138 | Measuring *SSP120* 5’ UTR | this study |
| LHZ1335 | *UBI4* 5’ UTR was introduced into the *Xho* I site of LHZ1138 | LHZ1138 | Measuring *UBI4* 5’ UTR | this study |
| LHZ1336 | *JEN1* 5’ UTR was introduced into the *Xho* I site of LHZ1138 | LHZ1138 | Measuring *JEN1* 5’ UTR | this study |
| LHZ1337 | *VPS21* 5’ UTR was introduced into the *Xho* I site of LHZ1138 | LHZ1138 | Measuring *VPS21* 5’ UTR | this study |
| LHZ1338 | *NHP2* 5’ UTR was introduced into the *Xho* I site of LHZ1138 | LHZ1138 | Measuring *NHP2* 5’ UTR | this study |
| LHZ1339 | *KLMA*_*40301* 5’ UTR was introduced into the *Xho* I site of LHZ1138 | LHZ1138 | Measuring *KLMA*_*40301* 5’ UTR | this study |
| LHZ1340 | *VMA5* 5’ UTR was introduced into the *Xho* I site of LHZ1138 | LHZ1138 | Measuring *VMA5* 5’ UTR | this study |
| LHZ1341 | *SOR1* 5’ UTR was introduced into the *Xho* I site of LHZ1138 | LHZ1138 | Measuring *SOR1* 5’ UTR | this study |
| LHZ1342 | *KYE1* 5’ UTR was introduced into the *Xho* I site of LHZ1138 | LHZ1138 | Measuring *KYE1* 5’ UTR | this study |
| LHZ1343 | *MDH2* 5’ UTR was introduced into the *Xho* I site of LHZ1138 | LHZ1138 | Measuring *MDH2* 5’ UTR | this study |
| LHZ1344 | *FSF1* 5’ UTR was introduced into the *Xho* I site of LHZ1138 | LHZ1138 | Measuring *FSF1* 5’ UTR | this study |
| LHZ1345 | *KLMA*_*50610* 5’ UTR was introduced into the *Xho* I site of LHZ1138 | LHZ1138 | Measuring *KLMA*_*50610* 5’ UTR | this study |
| LHZ1346 | *RSM23* 5’ UTR was introduced into the *Xho* I site of LHZ1138 | LHZ1138 | Measuring *RSM23* 5’ UTR | this study |
| LHZ1347 | *RSM27* 5’ UTR was introduced into the *Xho* I site of LHZ1138 | LHZ1138 | Measuring *RSM27* 5’ UTR | this study |
| LHZ1348 | *KLMA*_*30482* 5’ UTR was introduced into the *Xho* I site of LHZ1138 | LHZ1138 | Measuring *KLMA*_*30482* 5’ UTR | this study |
| LHZ1349 | *FBP1* 5’ UTR was introduced into the *Xho* I site of LHZ1138 | LHZ1138 | Measuring *FBP1* 5’ UTR | this study |
| LHZ1350 | *GLC8* 5’ UTR was introduced into the *Xho* I site of LHZ1138 | LHZ1138 | Measuring *GLC8* 5’ UTR | this study |
| LHZ1351 | *QCR2* 5’ UTR was introduced into the *Xho* I site of LHZ1138 | LHZ1138 | Measuring *QCR2* 5’ UTR | this study |
| LHZ1352 | *YAR1* 5’ UTR was introduced into the *Xho* I site of LHZ1138 | LHZ1138 | Measuring *YAR1* 5’ UTR | this study |
| LHZ1353 | *CLU1* 5’ UTR was introduced into the *Xho* I site of LHZ1138 | LHZ1138 | Measuring *CLU1* 5’ UTR | this study |
| LHZ1354 | *RRP45* 5’ UTR was introduced into the *Xho* I site of LHZ1138 | LHZ1138 | Measuring *RRP45* 5’ UTR | this study |
| LHZ1355 | *SSZ1* 5’ UTR was introduced into the *Xho* I site of LHZ1138 | LHZ1138 | Measuring *SSZ1* 5’ UTR | this study |
| LHZ1356 | *CBP6* 5’ UTR was introduced into the *Xho* I site of LHZ1138 | LHZ1138 | Measuring *CBP6* 5’ UTR | this study |
| LHZ1357 | *SSA3* 5’ UTR was introduced into the *Xho* I site of LHZ1138 | LHZ1138 | Measuring *SSA3* 5’ UTR | this study |
| LHZ1358 | *TVP18* 5’ UTR was introduced into the *Xho* I site of LHZ1138 | LHZ1138 | Measuring *TVP18* 5’ UTR | this study |
| LHZ1359 | *DBP10* 5’ UTR was introduced into the *Xho* I site of LHZ1138 | LHZ1138 | Measuring *DBP10* 5’ UTR | this study |
| LHZ1360 | *EHT1* 5’ UTR was introduced into the *Xho* I site of LHZ1138 | LHZ1138 | Measuring *EHT1* 5’ UTR | this study |
| LHZ1361 | *PKP1* 5’ UTR was introduced into the *Xho* I site of LHZ1138 | LHZ1138 | Measuring *PKP1* 5’ UTR | this study |
| LHZ1362 | *HTB1* 5’ UTR was introduced into the *Xho* I site of LHZ1138 | LHZ1138 | Measuring *HTB1* 5’ UTR | this study |
| LHZ1363 | *HTB1* 5’ UTR was introduced into the *Xho* I site of LHZ1138 | LHZ1138 | Measuring *HTB1* 5’ UTR | this study |
| LHZ1364 | *NCE102* 5’ UTR was introduced into the *Xho* I site of LHZ1138 | LHZ1138 | Measuring *NCE102* 5’ UTR | this study |
| LHZ1365 | *KLMA*_*60481* 5’ UTR was introduced into the *Xho* I site of LHZ1138 | LHZ1138 | Measuring *KLMA*_*60481* 5’ UTR | this study |
| LHZ1366 | *NSR1* 5’ UTR was introduced into the *Xho* I site of LHZ1138 | LHZ1138 | Measuring *NSR1* 5’ UTR | this study |
| LHZ1367 | *SLD5* 5’ UTR was introduced into the *Xho* I site of LHZ1138 | LHZ1138 | Measuring *SLD5* 5’ UTR | this study |
| LHZ1368 | *HCH1* 5’ UTR was introduced into the *Xho* I site of LHZ1138 | LHZ1138 | Measuring *HCH1* 5’ UTR | this study |
| LHZ1369 | *ADH1* 5’ UTR was introduced into the *Xho* I site of LHZ1138 | LHZ1138 | Measuring *ADH1* 5’ UTR | this study |
| LHZ1370 | *POR1* 5’ UTR was introduced into the *Xho* I site of LHZ1138 | LHZ1138 | Measuring *POR1* 5’ UTR | this study |
| LHZ1371 | *INO1* 5’ UTR was introduced into the *Xho* I site of LHZ1138 | LHZ1138 | Measuring *INO1* 5’ UTR | this study |
| LHZ1372 | *ADH2* 5’ UTR was introduced into the *Xho* I site of LHZ1138 | LHZ1138 | Measuring *ADH2* 5’ UTR | this study |
| LHZ1373 | *HGT1* 5’ UTR was introduced into the *Xho* I site of LHZ1138 | LHZ1138 | Measuring *HGT1* 5’ UTR | this study |
| LHZ1374 | *TIM9* 5’ UTR was introduced into the *Xho* I site of LHZ1138 | LHZ1138 | Measuring *TIM9* 5’ UTR | this study |
| LHZ1375 | *SAM2* 5’ UTR was introduced into the *Xho* I site of LHZ1138 | LHZ1138 | Measuring *SAM2* 5’ UTR | this study |
| LHZ1376 | *MRP49* 5’ UTR was introduced into the *Xho* I site of LHZ1138 | LHZ1138 | Measuring *MRP49* 5’ UTR | this study |
| LHZ1377 | *KLMA*_*60492* 5’ UTR was introduced into the *Xho* I site of LHZ1138 | LHZ1138 | Measuring *KLMA*_*60492* 5’ UTR | this study |
| LHZ1378 | *NCB2* 5’ UTR was introduced into the *Xho* I site of LHZ1138 | LHZ1138 | Measuring *NCB2* 5’ UTR | this study |
| LHZ1379 | *TIM9* len2**^<3>^** 5’ UTR was introduced into the *Xho* I site of LHZ1138 | LHZ1138 | Measuring *TIM9* len2 5’ UTR | this study |
| LHZ1380 | *TIM9* len5 5’ UTR was introduced into the *Xho* I site of LHZ1138 | LHZ1138 | Measuring *TIM9* len5 5’ UTR | this study |
| LHZ1381 | *TIM9* len6 5’ UTR was introduced into the *Xho* I site of LHZ1138 | LHZ1138 | Measuring *TIM9* len6 5’ UTR | this study |
| LHZ1382 | *TIM9* len11 5’ UTR was introduced into the *Xho* I site of LHZ1138 | LHZ1138 | Measuring *TIM9* len11 5’ UTR | this study |
| LHZ1383 | *TIM9* u67**^<4>^** 5’ UTR was introduced into the *Xho* I site of LHZ1138 | LHZ1138 | Measuring *TIM9* u67 5’ UTR | this study |
| LHZ1384 | *TIM9* u60 5’ UTR was introduced into the *Xho* I site of LHZ1138 | LHZ1138 | Measuring *TIM9* u60 5’ UTR | this study |
| LHZ1385 | *TIM9* u39 5’ UTR was introduced into the *Xho* I site of LHZ1138 | LHZ1138 | Measuring *TIM9* u39 5’ UTR | this study |
| LHZ1386 | *TIM9* d4**^<5>^** 5’ UTR was introduced into the *Xho* I site of LHZ1138 | LHZ1138 | Measuring *TIM9* d4 5’ UTR | this study |
| LHZ1387 | *SSZ1* len1 5’ UTR was introduced into the *Xho* I site of LHZ1138 | LHZ1138 | Measuring *SSZ1* len1 5’ UTR | this study |
| LHZ1388 | *SSZ1* len2 5’ UTR was introduced into the *Xho* I site of LHZ1138 | LHZ1138 | Measuring *SSZ1* len2 5’ UTR | this study |
| LHZ1389 | *SSZ1* len8 5’ UTR was introduced into the *Xho* I site of LHZ1138 | LHZ1138 | Measuring *SSZ1* len8 5’ UTR | this study |
| LHZ1390 | *SSZ1* len10 5’ UTR was introduced into the *Xho* I site of LHZ1138 | LHZ1138 | Measuring *SSZ1* len10 5’ UTR | this study |
| LHZ1391 | *SSZ1* u75 5’ UTR was introduced into the *Xho* I site of LHZ1138 | LHZ1138 | Measuring *SSZ1* u75 5’ UTR | this study |
| LHZ1392 | *SSZ1* u61 5’ UTR was introduced into the *Xho* I site of LHZ1138 | LHZ1138 | Measuring *SSZ1* u61 5’ UTR | this study |
| LHZ1393 | *SSZ1* u40 5’ UTR was introduced into the *Xho* I site of LHZ1138 | LHZ1138 | Measuring *SSZ1* u40 5’ UTR | this study |
| LHZ1394 | *SSZ1* d5 5’ UTR was introduced into the *Xho* I site of LHZ1138 | LHZ1138 | Measuring *SSZ1* d5 5’ UTR | this study |
| LHZ1395 | *FIM1*_*2796* len0 5’ UTR was introduced into the *Xho* I site of LHZ1138 | LHZ1138 | Measuring *FIM1*_*2796* len0 5’ UTR | this study |
| LHZ1396 | *FIM1*_*2796* len1 5’ UTR was introduced into the *Xho* I site of LHZ1138 | LHZ1138 | Measuring *FIM1*_*2796* len1 5’ UTR | this study |
| LHZ1397 | *FIM1*_*2796* len9 5’ UTR was introduced into the *Xho* I site of LHZ1138 | LHZ1138 | Measuring *FIM1*_*2796* len9 5’ UTR | this study |
| LHZ1398 | *FIM1*_*2796* len10 5’ UTR was introduced into the *Xho* I site of LHZ1138 | LHZ1138 | Measuring *FIM1*_*2796* len10 5’ UTR | this study |
| LHZ1399 | *FIM1*_*2796* u114 5’ UTR was introduced into the *Xho* I site of LHZ1138 | LHZ1138 | Measuring *FIM1*_*2796* u114 5’ UTR | this study |
| LHZ1400 | *FIM1*_*2796* u103 5’ UTR was introduced into the *Xho* I site of LHZ1138 | LHZ1138 | Measuring *FIM1*_*2796* u103 5’ UTR | this study |
| LHZ1401 | *FIM1*_*2796* u81 5’ UTR was introduced into the *Xho* I site of LHZ1138 | LHZ1138 | Measuring *FIM1*_*2796* u81 5’ UTR | this study |
| LHZ1402 | *FIM1*_*2796* d48 5’ UTR was introduced into the *Xho* I site of LHZ1138 | LHZ1138 | Measuring *FIM1*_*2796* d48 5’ UTR | this study |
| LHZ1403 | *QRI1* len0 5’ UTR was introduced into the *Xho* I site of LHZ1138 | LHZ1138 | Measuring *QRI1* len0 5’ UTR | this study |
| LHZ1404 | *QRI1* len3 5’ UTR was introduced into the *Xho* I site of LHZ1138 | LHZ1138 | Measuring *QRI1* len3 5’ UTR | this study |
| LHZ1405 | *QRI1* len9 5’ UTR was introduced into the *Xho* I site of LHZ1138 | LHZ1138 | Measuring *QRI1* len9 5’ UTR | this study |
| LHZ1406 | *QRI1* len10 5’ UTR was introduced into the *Xho* I site of LHZ1138 | LHZ1138 | Measuring *QRI1* len10 5’ UTR | this study |
| LHZ1407 | *QRI1* u94 5’ UTR was introduced into the *Xho* I site of LHZ1138 | LHZ1138 | Measuring *QRI1* u94 5’ UTR | this study |
| LHZ1408 | *QRI1* u76 5’ UTR was introduced into the *Xho* I site of LHZ1138 | LHZ1138 | Measuring *QRI1* u76 5’ UTR | this study |
| LHZ1409 | *QRI1* d40 5’ UTR was introduced into the *Xho* I site of LHZ1138 | LHZ1138 | Measuring *QRI1* d40 5’ UTR | this study |
| LHZ1410 | *QRI1* d22 5’ UTR was introduced into the *Xho* I site of LHZ1138 | LHZ1138 | Measuring *QRI1* d22 5’ UTR | this study |
| LHZ1411 | *SSP120* len0 5’ UTR was introduced into the *Xho* I site of LHZ1138 | LHZ1138 | Measuring *SSP120* len0 5’ UTR | this study |
| LHZ1412 | *SSP120* len3 5’ UTR was introduced into the *Xho* I site of LHZ1138 | LHZ1138 | Measuring *SSP120* len3 5’ UTR | this study |
| LHZ1413 | *SSP120* u43 5’ UTR was introduced into the *Xho* I site of LHZ1138 | LHZ1138 | Measuring *SSP120* u43 5’ UTR | this study |
| LHZ1414 | *SSP120* u22 5’ UTR was introduced into the *Xho* I site of LHZ1138 | LHZ1138 | Measuring *SSP120* u22 5’ UTR | this study |
| LHZ1415 | *RNA1* len3 5’ UTR was introduced into the *Xho* I site of LHZ1138 | LHZ1138 | Measuring *RNA1* len3 5’ UTR | this study |
| LHZ1416 | *RNA1* len11 5’ UTR was introduced into the *Xho* I site of LHZ1138 | LHZ1138 | Measuring *RNA1* len11 5’ UTR | this study |
| LHZ1417 | *RNA1* u34 5’ UTR was introduced into the *Xho* I site of LHZ1138 | LHZ1138 | Measuring *RNA1* u34 5’ UTR | this study |
| LHZ1418 | *RNA1* u18 5’ UTR was introduced into the *Xho* I site of LHZ1138 | LHZ1138 | Measuring *RNA1* u18 5’ UTR | this study |
| LHZ1419 | *INU1* len0 5’ UTR was introduced into the *Xho* I site of LHZ1138 | LHZ1138 | Measuring *INU1* len0 5’ UTR | this study |
| LHZ1420 | *INU1* len1 5’ UTR was introduced into the *Xho* I site of LHZ1138 | LHZ1138 | Measuring *INU1* len1 5’ UTR | this study |
| LHZ1421 | *INU1* u136 5’ UTR was introduced into the *Xho* I site of LHZ1138 | LHZ1138 | Measuring *INU1* u136 5’ UTR | this study |
| LHZ1422 | *INU1* u108 5’ UTR was introduced into the *Xho* I site of LHZ1138 | LHZ1138 | Measuring *INU1* u108 5’ UTR | this study |
| LHZ1423 | *INU1* u97 5’ UTR was introduced into the *Xho* I site of LHZ1138 | LHZ1138 | Measuring *INU1* u97 5’ UTR | this study |
| LHZ1424 | *TIM9* u53 5’ UTR was introduced into the *Xho* I site of LHZ1138 | LHZ1138 | Measuring *TIM9* u53 5’ UTR | this study |
| LHZ1425 | *KLMA*_*60072* len1 5’ UTR was introduced into the *Xho* I site of LHZ1138 | LHZ1138 | Measuring *KLMA*_*60072* len1 5’ UTR | this study |
| LHZ1426 | *KLMA*_*60072* len2 5’ UTR was introduced into the *Xho* I site of LHZ1138 | LHZ1138 | Measuring *KLMA*_*60072* len2 5’ UTR | this study |
| LHZ1427 | *KLMA*_*60072* u78 5’ UTR was introduced into the *Xho* I site of LHZ1138 | LHZ1138 | Measuring *KLMA*_*60072* u78 5’ UTR | this study |
| LHZ1428 | *KLMA*_*60072* u24 5’ UTR was introduced into the *Xho* I site of LHZ1138 | LHZ1138 | Measuring *KLMA*_*60072* u24 5’ UTR | this study |
| LHZ1429 | *GRE2* len0 5’ UTR was introduced into the *Xho* I site of LHZ1138 | LHZ1138 | Measuring *GRE2* len0 5’ UTR | this study |
| LHZ1430 | *KLMA*_*40607* len0 5’ UTR was introduced into the *Xho* I site of LHZ1138 | LHZ1138 | Measuring *KLMA*_*40607* len0 5’ UTR | this study |
| LHZ1431 | *PRS3* len0 5’ UTR was introduced into the *Xho* I site of LHZ1138 | LHZ1138 | Measuring *PRS3* len0 5’ UTR | this study |
| LHZ1432 | *DBP9* len0 5’ UTR was introduced into the *Xho* I site of LHZ1138 | LHZ1138 | Measuring *DBP9* len0 5’ UTR | this study |
| LHZ1433 | *PSY2* len0 5’ UTR was introduced into the *Xho* I site of LHZ1138 | LHZ1138 | Measuring *PSY2* len0 5’ UTR | this study |
| LHZ1434 | *OLE1* len0 5’ UTR was introduced into the *Xho* I site of LHZ1138 | LHZ1138 | Measuring *OLE1* len0 5’ UTR | this study |
| LHZ1435 | *USA1* len0 5’ UTR was introduced into the *Xho* I site of LHZ1138 | LHZ1138 | Measuring *USA1* len0 5’ UTR | this study |
| LHZ1436 | *CDC12* len0 5’ UTR was introduced into the *Xho* I site of LHZ1138 | LHZ1138 | Measuring *CDC12* len0 5’ UTR | this study |
| LHZ1437 | *COP1* len0 5’ UTR was introduced into the *Xho* I site of LHZ1138 | LHZ1138 | Measuring *COP1* len0 5’ UTR | this study |
| LHZ1438 | *SSH4* len0 5’ UTR was introduced into the *Xho* I site of LHZ1138 | LHZ1138 | Measuring *SSH4* len0 5’ UTR | this study |
| LHZ1439 | *KLMA*_*80280* len0 5’ UTR was introduced into the *Xho* I site of LHZ1138 | LHZ1138 | Measuring *KLMA*_*80280* len0 5’ UTR | this study |
| LHZ1440 | *SVF1* len0 5’ UTR was introduced into the *Xho* I site of LHZ1138 | LHZ1138 | Measuring *SVF1* len0 5’ UTR | this study |
| pZP32 | pKS, KD, P&5’ UTR_INU1_-SS_INU1_-*Est1E*-*His_6_*-T_INU1_, *URA3* | / | / | <6> |
| LHZ766 | pMD18-T, pKD1, P&5’ UTR_TEF_-α signal peptide-*AnFaeA*-T_TEF_, *URA3* | / | Provide template of *AnFaeA* | <7> |
| LHZ1441 | pKS, pKD, *HXT4* promoter-*Sma* I-mutant *INU1* signal peptide (harboring a P10L mutation)-*AnFaeA*-*INU1* terminator-*KmURA3* | pZP32 | Backbone vector for measuring AnFaeA abundance caused by various 5’ UTR | this study |
| LHZ1442 | *SSH4* 5’ UTR was introduced into *Sma* I of LHZ1442 | LHZ1441 | Measuring *SSH4* 5’ UTR | this study |
| LHZ1443 | *SSH4* len0 5’ UTR was introduced into *Sma* I of LHZ1442 | LHZ1441 | Measuring *SSH4* len0 5’ UTR | this study |
| LHZ1444 | *INU1* 5’ UTR was introduced into *Sma* I of LHZ1442 | LHZ1441 | Measuring *INU1* 5’ UTR | this study |
| LHZ1445 | *INU1* len0 5’ UTR was introduced into *Sma* I of LHZ1442 | LHZ1441 | Measuring *INU1* len0 5’ UTR | this study |
| LHZ1446 | *KLMA*_*80280* 5’ UTR was introduced into *Sma* I of LHZ1442 | LHZ1441 | Measuring *KLMA*_*80280* 5’ UTR | this study |
| LHZ1447 | *KLMA*_*80280* len0 5’ UTR was introduced into *Sma* I of LHZ1442 | LHZ1441 | Measuring *KLMA*_*80280* len0 5’ UTR | this study |
| pZP28 | pKS, KD, P&5’ UTR_INU1_-SS_INU1_-*Est1E*-T_INU1_, *URA3* | / | / | <6> |
| LHZ1448 | pKS, KD, P&5’ UTR_INU1_-SS_INU1_-*AnFaeA*-T_INU1_, *URA3* | pZP28 | Measuring *INU1* wild-type 5’ UTR and serving as the template of mutagenesis PCR to obtain LHZ1449 | this study |
| LHZ1449 | The *INU1* wild-type 5’ UTR of LHZ1448 was replaced by *INU1* len0 5’ UTR | LHZ1448 | Measuring *INU1* len0 5’ UTR and serving as the template of mutagenesis PCR to obtain LHZ1450~LHZ1462 | this study |
| LHZ1450 | *INU1* len0 5’ UTR of LHZ1449 was replaced by *INU1* len3-d16 5’ UTR | LHZ1449 | Measuring *INU1* len3-d16 5’ UTR | this study |
| LHZ1451 | *INU1* len0 5’ UTR of LHZ1449 was replaced by *INU1* len4-d20 5’ UTR | LHZ1449 | Measuring *INU1* len4-d20 5’ UTR | this study |
| LHZ1452 | *INU1* len0 5’ UTR of LHZ1449 was replaced by *INU1* len4-d26 5’ UTR | LHZ1449 | Measuring *INU1* len4-d26 5’ UTR | this study |
| LHZ1453 | *INU1* len0 5’ UTR of LHZ1449 was replaced by *INU1* len2-d27 5’ UTR | LHZ1449 | Measuring *INU1* len2-d27 5’ UTR | this study |
| LHZ1454 | *INU1* len0 5’ UTR of LHZ1449 was replaced by *INU1* len2 5’ UTR | LHZ1449 | Measuring *INU1* len2 5’ UTR | this study |
| LHZ1455 | *INU1* len0 5’ UTR of LHZ1449 was replaced by *INU1* len3-d39 5’ UTR | LHZ1449 | Measuring *INU1* len3-d39 5’ UTR | this study |
| LHZ1456 | *INU1* len0 5’ UTR of LHZ1449 was replaced by *INU1* len8-d61 5’ UTR | LHZ1449 | Measuring *INU1* len8-d61 5’ UTR | this study |
| LHZ1457 | *INU1* len0 5’ UTR of LHZ1449 was replaced by *INU1* len4-d64 5’ UTR | LHZ1449 | Measuring *INU1* len4-d64 5’ UTR | this study |
| LHZ1458 | *INU1* len0 5’ UTR of LHZ1449 was replaced by *INU1* len10-u96 5’ UTR | LHZ1449 | Measuring *INU1* len10-u96 5’ UTR | this study |
| LHZ1459 | *INU1* len0 5’ UTR of LHZ1449 was replaced by *INU1* len5-u73 5’ UTR | LHZ1449 | Measuring *INU1* len5-u73 5’ UTR | this study |
| LHZ1460 | *INU1* len0 5’ UTR of LHZ1449 was replaced by *INU1* len7-d32 5’ UTR | LHZ1449 | Measuring *INU1* len7-d32 5’ UTR | this study |
| LHZ1461 | *INU1* len0 5’ UTR of LHZ1449 was replaced by *INU1* len8-u87 5’ UTR | LHZ1449 | Measuring *INU1* len8-u87 5’ UTR | this study |
| LHZ1462 | *INU1* len0 5’ UTR of LHZ1449 was replaced by *INU1* len9-u146 5’ UTR | LHZ1449 | Measuring *INU1* len9-u146 5’ UTR | this study |

<1> Ag refers to *Ashbya gossypii*. Sc refers to *Saccharomyces cerevisiae*. Genes without abbreviations are from *Kluyveromyces marxianus*.

<2> Wu P, Zhou J, Yu Y*, Lu H*. 2022. Characterization of essential elements for improved episomal expressions in *Kluyveromyces marxianus*. ***Biotechnol J*** 17(4):e2100382

<3> lenN, where N refers to a number between 0 and 14, indicates that the longest poly(A) tract in the 5' UTR has been replaced by a continuous stretch of N adenine nucleotides.

<4> uN, where N refers to a number between 0 and 200, indicates that the longest and most downstream poly(A) tract in the 5’ UTR has been shifted upstream to a position that is N nucleotides away from the start codon.

<5> dN, where N refers to a number between 0 and 200, indicates that the longest and most downstream poly(A) tract in the 5’ UTR has been shifted downstream to a position that is N nucleotides away from the start codon.

<6> Zhou J, Zhu P, Hu X, Lu H, Yu Y*. 2018. Improved secretory expression of lignocellulolytic enzymes in *Kluyveromyces marxianus* by promoter and signal sequence engineering. ***Biotechnol Biofuels*** 11:235.

<7> Shi T, Zhou J, Xue A, Lu H, He Y, Yu Y*. 2021. Characterization and modulation of endoplasmic reticulum stress response target genes in *Kluyveromyces marxianus* to improve secretory expressions of heterologous proteins. ***Biotechnol Biofuels*** 14:236.

# Table S2 List of primers

| Name | Sequence (5’ → 3’) | *Application* |
| --- | --- | --- |
| OZJY1F | CTTTTGAATGCTCAGACCCCGCCAACGCCTTAAGCTTATG | The overlap extension PCR for inserting *SPP381* 5’ UTR into *Xho* *I* site of LHZ1138 |
| OZJY1R | GTGAAAAGTTCTTCTCCTTTACTCATTCTCTAAAGTGCATCGTTCATCTCTTG | The overlap extension PCR for inserting *SPP381* 5’ UTR into *Xho* *I* site of LHZ1138 |
| OZJY2F | CTTTTGAATGCTCAGACCCCGACGACAACATAGATGTGTATGGAAGAAG | The amplification of *GUK1* 5’ UTR with homologous arms for insertion into *Xho* I site of LHZ1138 |
| OZJY2R | GTGAAAAGTTCTTCTCCTTTACTCATACAATGTCGGGACGATGCTTAC | The amplification of *GUK1* 5’ UTR with homologous arms for insertion into *Xho* I site of LHZ1138 |
| OZJY3F | CTTTTGAATGCTCAGACCCCGCCTGTTCCTCTGACAAATCG | The overlap extension PCR for inserting *SRX1* 5’ UTR into *Xho* *I* site of LHZ1138 |
| OZJY3R | GTGAAAAGTTCTTCTCCTTTACTCATGGTGTGGATAGGCTGGTG | The overlap extension PCR for inserting *SRX1* 5’ UTR into *Xho* *I* site of LHZ1138 |
| OZJY4F | CTTTTGAATGCTCAGACCCCGTGGTCAAAGTAATAAAAGCTTTGTTAAAAAG | The overlap extension PCR for inserting *SEC53* 5’ UTR into *Xho* *I* site of LHZ1138 |
| OZJY4R | GTGAAAAGTTCTTCTCCTTTACTCATTGTGTGGTATTATGGGGCGG | The overlap extension PCR for inserting *SEC53* 5’ UTR into *Xho* *I* site of LHZ1138 |
| OZJY5F | CTTTTGAATGCTCAGACCCCAAAAACATCAACTATTTCCATTGCAAC | The overlap extension PCR for inserting *OST1* 5’ UTR into *Xho* *I* site of LHZ1138 |
| OZJY5R | GAAAAGTTCTTCTCCTTTACTCATTCTTTTCAATATTTAACTGATTGATTTGTTACTTAG | The overlap extension PCR for inserting *OST1* 5’ UTR into *Xho* *I* site of LHZ1138 |
| OZJY6F | CTTTTGAATGCTCAGACCCCGCCCCACGATTACACACACG | The amplification of *KLMA*_*80108* 5’ UTR with homologous arms for insertion into *Xho* I site of LHZ1138 |
| OZJY6R | GTGAAAAGTTCTTCTCCTTTACTCATCAACTTGTCTTTAGAAGACCCTTTTCCTA | The amplification of *KLMA*_*80108* 5’ UTR with homologous arms for insertion into *Xho* I site of LHZ1138 |
| OZJY7F | CTTTTGAATGCTCAGACCCCTTGCACATCATCGCTCTCGCATC | The amplification of *RPL5* 5’ UTR with homologous arms for insertion into *Xho* I site of LHZ1138 |
| OZJY7R | GTGAAAAGTTCTTCTCCTTTACTCATGGTCGACAGATCGCTACGTTACTG | The amplification of *RPL5* 5’ UTR with homologous arms for insertion into *Xho* I site of LHZ1138 |
| OZJY8F | CTTTTGAATGCTCAGACCCCGCTCTAATGAATACATAAAGGGAAGG | The overlap extension PCR for inserting *AIM2* 5’ UTR into *Xho* *I* site of LHZ1138 |
| OZJY8R | GAAAAGTTCTTCTCCTTTACTCATTTTGAGTTTGTTCTTTACAGTACCTAATATAAATTG | The overlap extension PCR for inserting *AIM2* 5’ UTR into *Xho* *I* site of LHZ1138 |
| OZJY9F | CTTTTGAATGCTCAGACCCCGCGCTTCACATTTTCACATTTTTC | The overlap extension PCR for inserting *KLMA*_*60307* 5’ UTR into *Xho* *I* site of LHZ1138 |
| OZJY9R | GTGAAAAGTTCTTCTCCTTTACTCATTTCTCCTTACAATCCAAACACAATTGAC | The overlap extension PCR for inserting *KLMA*_*60307* 5’ UTR into *Xho* *I* site of LHZ1138 |
| OZJY10F | CTTTTGAATGCTCAGACCCCGCAATGTTGCTGACAGAAATATCG | The overlap extension PCR for inserting *GLO4* 5’ UTR into *Xho* *I* site of LHZ1138 |
| OZJY10R | GTGAAAAGTTCTTCTCCTTTACTCATAACCTGTGGTCTAACCTTTGTG | The overlap extension PCR for inserting *GLO4* 5’ UTR into *Xho* *I* site of LHZ1138 |
| OZJY11F | CTTTTGAATGCTCAGACCCCGAACAAAAAAAGAACCCCGTGTC | The overlap extension PCR for inserting *FIM1*_*4812* 5’ UTR into *Xho* *I* site of LHZ1138 |
| OZJY11R | GTGAAAAGTTCTTCTCCTTTACTCATAGTAAGGTATGGGCCCAGAG | The overlap extension PCR for inserting *FIM1*_*4812* 5’ UTR into *Xho* *I* site of LHZ1138 |
| OZJY12F | CTTTTGAATGCTCAGACCCCAACATAGGCCTATCTGAAAAATTAACAGC | The overlap extension PCR for inserting *MAK10* 5’ UTR into *Xho* *I* site of LHZ1138 |
| OZJY12R | GTGAAAAGTTCTTCTCCTTTACTCATCACTGGCACGGCCAAAAAAAAAG | The overlap extension PCR for inserting *MAK10* 5’ UTR into *Xho* *I* site of LHZ1138 |
| OZJY13F | CTTTTGAATGCTCAGACCCCACCTAATGACACTATCTAATTCGTTAAATGTTTAC | The overlap extension PCR for inserting *RTC4* 5’ UTR into *Xho* *I* site of LHZ1138 |
| OZJY13R | GTGAAAAGTTCTTCTCCTTTACTCATTAAATATCCACGATCCCTAAGAGTTACAG | The overlap extension PCR for inserting *RTC4* 5’ UTR into *Xho* *I* site of LHZ1138 |
| OZJY14F | CTTTTGAATGCTCAGACCCCGTTAATAAATTATGTTATAATTCTATAAAAAGGGTAAAG | The overlap extension PCR for inserting *KLMA*_*20131* 5’ UTR into *Xho* *I* site of LHZ1138 |
| OZJY14R | GTGAAAAGTTCTTCTCCTTTACTCATGCTGTGGGTTATAGTGTCGTTAC | The overlap extension PCR for inserting *KLMA*_*20131* 5’ UTR into *Xho* *I* site of LHZ1138 |
| OZJY15F | CTTTTGAATGCTCAGACCCCGTCTCCACTGTTCTATACTGCG | The overlap extension PCR for inserting *KLMA*_*70012* 5’ UTR into *Xho* *I* site of LHZ1138 |
| OZJY15R | GTGAAAAGTTCTTCTCCTTTACTCATAATGCTTATTAAGTGGGATAACGTAAAG | The overlap extension PCR for inserting *KLMA*_*70012* 5’ UTR into *Xho* *I* site of LHZ1138 |
| OZJY16F | CTTTTGAATGCTCAGACCCCGGAAGATCAACGAAAAGAACTGAGAAG | The overlap extension PCR for inserting *CAR2* 5’ UTR into *Xho* *I* site of LHZ1138 |
| OZJY16R | GTGAAAAGTTCTTCTCCTTTACTCATATTTGCTATTTTTTACACTATTGTTTGATTGATG | The overlap extension PCR for inserting *CAR2* 5’ UTR into *Xho* *I* site of LHZ1138 |
| OZJY17F | CTTTTGAATGCTCAGACCCCTTCTTGAAATTATACTATGTAAAAAGTTGTATATACAC | The overlap extension PCR for inserting *LAS17* 5’ UTR into *Xho* *I* site of LHZ1138 |
| OZJY17R | GTGAAAAGTTCTTCTCCTTTACTCATTTTTTTTTCTTCAGGGATAGTCCTAATTAC | The overlap extension PCR for inserting *LAS17* 5’ UTR into *Xho* *I* site of LHZ1138 |
| OZJY18F | CTTTTGAATGCTCAGACCCCGGCGGTTGATTAGAAGAGATGAAG | The overlap extension PCR for inserting *SVF1* 5’ UTR into *Xho* *I* site of LHZ1138 |
| OZJY18R | TGAAAAGTTCTTCTCCTTTACTCATTATCAATTATTTCCAAAATCTATATCTATTTCAAC | The overlap extension PCR for inserting *SVF1* 5’ UTR into *Xho* *I* site of LHZ1138 |
| OZJY19F | CTTTTGAATGCTCAGACCCCGACAAATGAGTGGCACCAGG | The overlap extension PCR for inserting *SIW14* 5’ UTR into *Xho* *I* site of LHZ1138 |
| OZJY19R | GAAAAGTTCTTCTCCTTTACTCATGCTTTTTTCACTTTATATCTTTAAAGTCCTTATTTG | The overlap extension PCR for inserting *SIW14* 5’ UTR into *Xho* *I* site of LHZ1138 |
| OZJY20F | CTTTTGAATGCTCAGACCCCAAAAATAGTTCAAGTAACCTGGCATC | The overlap extension PCR for inserting *KLMA*_*80280* 5’ UTR into *Xho* *I* site of LHZ1138 |
| OZJY20R | GTGAAAAGTTCTTCTCCTTTACTCATCGAACAATGAATCAAAGATAGAAGAAC | The overlap extension PCR for inserting *KLMA*_*80280* 5’ UTR into *Xho* *I* site of LHZ1138 |
| OZJY21F | CTTTTGAATGCTCAGACCCCGAGGTTTGTGTTTCTTAGTAGAATTATTATCC | The overlap extension PCR for inserting *LAC4* 5’ UTR into *Xho* *I* site of LHZ1138 |
| OZJY21R | GTGAAAAGTTCTTCTCCTTTACTCATATTTTTCAATTCTCGATGAGTATATGAGTG | The overlap extension PCR for inserting *LAC4* 5’ UTR into *Xho* *I* site of LHZ1138 |
| OZJY22F | CTTTTGAATGCTCAGACCCCCAAAACCTTTAATATGGGAAAAGAAGCTTTG | The overlap extension PCR for inserting *ZPR1* 5’ UTR into *Xho* *I* site of LHZ1138 |
| OZJY22R | GTGAAAAGTTCTTCTCCTTTACTCATCGCAAAAAATATATGTATCCAGAAACTATAATAC | The overlap extension PCR for inserting *ZPR1* 5’ UTR into *Xho* *I* site of LHZ1138 |
| OZJY23F | CTTTTGAATGCTCAGACCCCGCCTGATAAACGTCTGATAAGTCTAG | The overlap extension PCR for inserting *GPT2* 5’ UTR into *Xho* *I* site of LHZ1138 |
| OZJY23R | GTGAAAAGTTCTTCTCCTTTACTCATTCTATTATTTCTGTGAGAGTAGGAAAACCAC | The overlap extension PCR for inserting *GPT2* 5’ UTR into *Xho* *I* site of LHZ1138 |
| OZJY24F | CATCCAATTACCACCAAAAAGTAATCAAGAGTCCCACCGTGCTCAAATACTACTCTAAATGAGTAAAGGAGAAGAACTTTTCAC | The amplification of *KLMA*_*50514* 5’ UTR with homologous arms for insertion into *Xho* I site of LHZ1138 |
| OZJY24R | CTTTTTGGTGGTAATTGGATGTATAATTTCCTTAATATGCGGGGTCTGAGCATTCAAAAG | The amplification of *KLMA*_*50514* 5’ UTR with homologous arms for insertion into *Xho* I site of LHZ1138 |
| OZJY25F | GAAAAGGCTAATTTCATGTTCAAGGATTGATATCAGAAAAATAAGATATTACGTCCGTTCAAATGAGTAAAGGAGAAGAACTTTTCAC | The amplification of *SNO3* 5’ UTR with homologous arms for insertion into *Xho* I site of LHZ1138 |
| OZJY25R | CCTTGAACATGAAATTAGCCTTTTCTATTTACAATTAACGCATCAAAAGTTTAAAATGGATTCGGGGTCTGAGCATTCAAAAG | The amplification of *SNO3* 5’ UTR with homologous arms for insertion into *Xho* I site of LHZ1138 |
| OZJY26F | CTTTTGAATGCTCAGACCCCAATTAAATCCGGGGTAAGGAAGAATTAC | The overlap extension PCR for inserting *INU1* 5’ UTR into *Xho* *I* site of LHZ1138 |
| OZJY26R | TGAAAAGTTCTTCTCCTTTACTCATATCTAACAAAAAAAAAATTAAATGTGTCACTTATG | The overlap extension PCR for inserting *INU1* 5’ UTR into *Xho* *I* site of LHZ1138 |
| OZJY27F | CTTTTGAATGCTCAGACCCCGAGACGAGACAGACGGAC | The overlap extension PCR for inserting *FIM1*_*2796* 5’ UTR into *Xho* *I* site of LHZ1138 |
| OZJY27R | GTGAAAAGTTCTTCTCCTTTACTCATCTCGAGCTCATTCCCCGG | The overlap extension PCR for inserting *FIM1*_*2796* 5’ UTR into *Xho* *I* site of LHZ1138 |
| OZJY28F | CCAATCCATAGTCATTAGTCGAGAGTCTCCTAATGAGTAAAGGAGAAGAACTTTTCAC | The overlap extension PCR for inserting *ARC40* 5’ UTR into *Xho* *I* site of LHZ1138 |
| OZJY28R | CGACTAATGACTATGGATTGGTGTACGAATAAGCTAAAACAGTGCTTACACTAGGGTGGGGTCTGAGCATTCAAAAG | The overlap extension PCR for inserting *ARC40* 5’ UTR into *Xho* *I* site of LHZ1138 |
| OZJY29F | CTTTTGAATGCTCAGACCCCTTGCAGCGCTTTTTACGAG | The overlap extension PCR for inserting *SKN7* 5’ UTR into *Xho* *I* site of LHZ1138 |
| OZJY29R | GTGAAAAGTTCTTCTCCTTTACTCATCGCTCTTTTTACCACTAAATCGTG | The overlap extension PCR for inserting *SKN7* 5’ UTR into *Xho* *I* site of LHZ1138 |
| OZJY30F | AAAGGAAAACTAATACATATATATATAAAATATATGAGTAAAGGAGAAGAACTTTTCAC | The amplification of *CPS1* 5’ UTR with homologous arms for insertion into *Xho* I site of LHZ1138 |
| OZJY30R | TATATATATGTATTAGTTTTCCTTTATTGGTTTTTGCCTGGGGTCTGAGCATTCAAAAG | The amplification of *CPS1* 5’ UTR with homologous arms for insertion into *Xho* I site of LHZ1138 |
| OZJY31F | ATTTCTTATATTGAAGGAGAAAAAAAAAATATTCCAAATTGCTCAGAGGGTGTAACTAGTACCAATGAGTAAAGGAGAAGAACTTTTCAC | The amplification of *ILV1* 5’ UTR with homologous arms for insertion into *Xho* I site of LHZ1138 |
| OZJY31R | CTTCAATATAAGAAATAATCCTCTCTCTCTCTGTATTGTTCTTAACTCCCTCGCTGCCAATTGAGCCTTAGGGGTCTGAGCATTCAAAAG | The amplification of *ILV1* 5’ UTR with homologous arms for insertion into *Xho* I site of LHZ1138 |
| OZJY32F | CTTTTGAATGCTCAGACCCCAGATCATTTTGAAAAGAGAAGGCGTC | The overlap extension PCR for inserting *DBP5* 5’ UTR into *Xho* *I* site of LHZ1138 |
| OZJY32R | GAAAAGTTCTTCTCCTTTACTCATTTTTTTTTTTTTTTCTATCGATAATTAGTCAATCTG | The overlap extension PCR for inserting *DBP5* 5’ UTR into *Xho* *I* site of LHZ1138 |
| OZJY33F | CTTTTGAATGCTCAGACCCCGTGTATTTTTGGATTGTAGATTCTCCAG | The overlap extension PCR for inserting *NDI1* 5’ UTR into *Xho* *I* site of LHZ1138 |
| OZJY33R | GTGAAAAGTTCTTCTCCTTTACTCATTGCTGATGAATATTGATATTATTACTCGC | The overlap extension PCR for inserting *NDI1* 5’ UTR into *Xho* *I* site of LHZ1138 |
| OZJY34F | CTTTTGAATGCTCAGACCCCAGAGCTGAATCTCACAACCAGATAC | The overlap extension PCR for inserting *SEC62* 5’ UTR into *Xho* *I* site of LHZ1138 |
| OZJY34R | GTGAAAAGTTCTTCTCCTTTACTCATTTTATTAGGATTTTAATTCTTCAGCTTCTCTAAC | The overlap extension PCR for inserting *SEC62* 5’ UTR into *Xho* *I* site of LHZ1138 |
| OZJY35F | CTTTTGAATGCTCAGACCCCGCTGGATCGCTTTTTCATTACG | The overlap extension PCR for inserting *FRS1* 5’ UTR into *Xho* *I* site of LHZ1138 |
| OZJY35R | GTGAAAAGTTCTTCTCCTTTACTCATATCGATGGTTTTAGTGTATCACAAAAG | The overlap extension PCR for inserting *FRS1* 5’ UTR into *Xho* *I* site of LHZ1138 |
| OZJY36F | CTTTTGAATGCTCAGACCCCTACTTTTTCCGGAAAGCACTGTTCGTTTAG | The amplification of *TNA1* 5’ UTR with homologous arms for insertion into *Xho* I site of LHZ1138 |
| OZJY36R | GTGAAAAGTTCTTCTCCTTTACTCATTCCAAAAATCCTGGTCTGGTCTTGTC | The amplification of *TNA1* 5’ UTR with homologous arms for insertion into *Xho* I site of LHZ1138 |
| OZJY37F | CTTTTGAATGCTCAGACCCCAAGTTACTTGATCAATTAGTACAGTTGCATTTTTATAC | The amplification of *NUP170* 5’ UTR with homologous arms for insertion into *Xho* I site of LHZ1138 |
| OZJY37R | GTGAAAAGTTCTTCTCCTTTACTCATCTCGAGAAACACGATGCTAACTGGTG | The amplification of *NUP170* 5’ UTR with homologous arms for insertion into *Xho* I site of LHZ1138 |
| OZJY38F | CTTTTGAATGCTCAGACCCCGACACCAGGTCATAGTGTATACTATATTG | The overlap extension PCR for inserting *rhb1* 5’ UTR into *Xho* *I* site of LHZ1138 |
| OZJY38R | GTGAAAAGTTCTTCTCCTTTACTCATCCTAACCCTTGTTATGCTCTTTTTTG | The overlap extension PCR for inserting *rhb1* 5’ UTR into *Xho* *I* site of LHZ1138 |
| OZJY39F | AATACATACCATTGCCCCGTCTCCCGTCGTTACAATGAGTAAAGGAGAAGAACTTTTCAC | The amplification of *NCE103* 5’ UTR with homologous arms for insertion into *Xho* I site of LHZ1138 |
| OZJY39R | ACGGGGCAATGGTATGTATTGATTTTTTTTGCTAGTGCTTGGGGTCTGAGCATTCAAAAG | The amplification of *NCE103* 5’ UTR with homologous arms for insertion into *Xho* I site of LHZ1138 |
| OZJY40F | ACCACCTAGCCTAGCGTCTACCTGTGAAAAAGCATGAGTAAAGGAGAAGAACTTTTCAC | The amplification of *ARC18* 5’ UTR with homologous arms for insertion into *Xho* I site of LHZ1138 |
| OZJY40R | CACAGGTAGACGCTAGGCTAGGTGGTGAGTTGGCGGGGTCTGAGCATTCAAAAG | The amplification of *ARC18* 5’ UTR with homologous arms for insertion into *Xho* I site of LHZ1138 |
| OZJY41F | CTTTTGAATGCTCAGACCCCAAAAAACACAGTCTTATAGCATGAAAAGG | The overlap extension PCR for inserting *PEX25* 5’ UTR into *Xho* *I* site of LHZ1138 |
| OZJY41R | GTGAAAAGTTCTTCTCCTTTACTCATTTTGCTTAATTCAATGGATACTCTATTGTC | The overlap extension PCR for inserting *PEX25* 5’ UTR into *Xho* *I* site of LHZ1138 |
| OZJY42F | CATTGTTGCAAGGAACTTGGACCGAAACTCATTCAACACTACTAAAAAATCCTAATAATGAGTAAAGGAGAAGAACTTTTCAC | The amplification of *SNU13* 5’ UTR with homologous arms for insertion into *Xho* I site of LHZ1138 |
| OZJY42R | GTCCAAGTTCCTTGCAACAATGAAAAGCTCGCTTGAATCTGGGGTCTGAGCATTCAAAAG | The amplification of *SNU13* 5’ UTR with homologous arms for insertion into *Xho* I site of LHZ1138 |
| OZJY43F | CTTTTGAATGCTCAGACCCCGATGTTTGCATAATTCTTTGGTGTGTTG | The overlap extension PCR for inserting *KLMA*_*80013* 5’ UTR into *Xho* *I* site of LHZ1138 |
| OZJY43R | AAAGTTCTTCTCCTTTACTCATAGTGTCTCAATTAATTAATTAATTAATTTTTTTTCCCG | The overlap extension PCR for inserting *KLMA*_*80013* 5’ UTR into *Xho* *I* site of LHZ1138 |
| OZJY44F | ATTGAAGGTTTTTAAGTGTGTTCTGTTTGTGTGGTTTTGTTTTTGTTTGTGTGTTGTAATAATCATGAGTAAAGGAGAAGAACTTTTCAC | The amplification of *SSH4* 5’ UTR with homologous arms for insertion into *Xho* I site of LHZ1138 |
| OZJY44R | ACACTTAAAAACCTTCAATAACCAACTGTAAACTTTTTTCGATGATTATAGCCTCACAAAACTGCTCTTTGGGGTCTGAGCATTCAAAAG | The amplification of *SSH4* 5’ UTR with homologous arms for insertion into *Xho* I site of LHZ1138 |
| OZJY45F | CTTTTGAATGCTCAGACCCCAAGCAAAGATCTCGTAAGGTTCAC | The overlap extension PCR for inserting *NEW1* 5’ UTR into *Xho* *I* site of LHZ1138 |
| OZJY45R | GTGAAAAGTTCTTCTCCTTTACTCATTTCTTCTTCTTTTTAGTTTGTGAGGC | The overlap extension PCR for inserting *NEW1* 5’ UTR into *Xho* *I* site of LHZ1138 |
| OZJY46F | CTTTTGAATGCTCAGACCCCGAGACTTCAGGCACAAAGTGAG | The overlap extension PCR for inserting *COP1* 5’ UTR into *Xho* *I* site of LHZ1138 |
| OZJY46R | GTGAAAAGTTCTTCTCCTTTACTCATGGCAAAAAGGTTCTAAGAAGTGTAC | The overlap extension PCR for inserting *COP1* 5’ UTR into *Xho* *I* site of LHZ1138 |
| OZJY47F | CTTTTGAATGCTCAGACCCCTAGTGTGTTACTTAACTTACCAATTCAATAG | The overlap extension PCR for inserting *GIM4* 5’ UTR into *Xho* *I* site of LHZ1138 |
| OZJY47R | GTGAAAAGTTCTTCTCCTTTACTCATTTTTTTTGTATGCGCTCACACATATTC | The overlap extension PCR for inserting *GIM4* 5’ UTR into *Xho* *I* site of LHZ1138 |
| OZJY48F | CTTTTGAATGCTCAGACCCCGAGTACTTTTAGTTTTCTTTTGGCTGC | The overlap extension PCR for inserting *CDC10* 5’ UTR into *Xho* *I* site of LHZ1138 |
| OZJY48R | GTGAAAAGTTCTTCTCCTTTACTCATTATTTTCAATTTTAAACCGACCTTTTTTTTG | The overlap extension PCR for inserting *CDC10* 5’ UTR into *Xho* *I* site of LHZ1138 |
| OZJY49F | CTTTTGAATGCTCAGACCCCGGTTGCTTAGTGAATTGGTGC | The overlap extension PCR for inserting *CDC12* 5’ UTR into *Xho* *I* site of LHZ1138 |
| OZJY49R | GTGAAAAGTTCTTCTCCTTTACTCATTCTAGTCTATATATAACCTATCTTTGCCTGTTC | The overlap extension PCR for inserting *CDC12* 5’ UTR into *Xho* *I* site of LHZ1138 |
| OZJY50F | CTTTTGAATGCTCAGACCCCGAATTCAGATCATAGACCCACCAC | The overlap extension PCR for inserting *LSM1* 5’ UTR into *Xho* *I* site of LHZ1138 |
| OZJY50R | GTGAAAAGTTCTTCTCCTTTACTCATTATTATATGACGTGTTAATGCTTTTTGAAGTTG | The overlap extension PCR for inserting *LSM1* 5’ UTR into *Xho* *I* site of LHZ1138 |
| OZJY51F | CTTTTGAATGCTCAGACCCCAGAGTTAAGAGTTTAGAGGGCTGAAG | The overlap extension PCR for inserting *DRE2* 5’ UTR into *Xho* *I* site of LHZ1138 |
| OZJY51R | GTGAAAAGTTCTTCTCCTTTACTCATCCTCGCTTGCTGTCTTCTC | The overlap extension PCR for inserting *DRE2* 5’ UTR into *Xho* *I* site of LHZ1138 |
| OZJY52F | CTTTTGAATGCTCAGACCCCGAATAAAGGAGGTTATAGCGGCG | The overlap extension PCR for inserting *YTA12* 5’ UTR into *Xho* *I* site of LHZ1138 |
| OZJY52R | TGAAAAGTTCTTCTCCTTTACTCATAAAGTCCTATTTTTTCACTCAAATATCCTCTATTC | The overlap extension PCR for inserting *YTA12* 5’ UTR into *Xho* *I* site of LHZ1138 |
| OZJY53F | CAGCAACCAAAAAACACAAACAAACACAGCTTCCAGAAAGCAAGCCCTTCCACCCTAGACAAGATGAGTAAAGGAGAAGAACTTTTCAC | The amplification of *QRI1* 5’ UTR with homologous arms for insertion into *Xho* I site of LHZ1138 |
| OZJY53R | GTTTGTTTGTGTTTTTTGGTTGCTGAATCGCCTCCCCTAATACCACACCAGTAAGTTATTATAAGATTCGGGGTCTGAGCATTCAAAAG | The amplification of *QRI1* 5’ UTR with homologous arms for insertion into *Xho* I site of LHZ1138 |
| OZJY54F | CTTTTGAATGCTCAGACCCCGGTATAAGAGGGATTGAGCAAGGGAG | The overlap extension PCR for inserting *SKG1* 5’ UTR into *Xho* *I* site of LHZ1138 |
| OZJY54R | GTGAAAAGTTCTTCTCCTTTACTCATTTGTCCCTTGTTAATTTGACCAATCTG | The overlap extension PCR for inserting *SKG1* 5’ UTR into *Xho* *I* site of LHZ1138 |
| OZJY55F | CTTTTGAATGCTCAGACCCCGACGCCAGATTGATTTGGTTTC | The overlap extension PCR for inserting *ACH1* 5’ UTR into *Xho* *I* site of LHZ1138 |
| OZJY55R | GTGAAAAGTTCTTCTCCTTTACTCATCTTTTCTCTATATTATTATCACTGCTGGC | The overlap extension PCR for inserting *ACH1* 5’ UTR into *Xho* *I* site of LHZ1138 |
| OZJY56F | CTTTTGAATGCTCAGACCCCGAGTTAAGGCTCTAGAGGTGTTGTATTTC | The overlap extension PCR for inserting *PET8* 5’ UTR into *Xho* *I* site of LHZ1138 |
| OZJY56R | GTGAAAAGTTCTTCTCCTTTACTCATTATGTCCTTCTATCTCTGCTTTTTTTTATTTCCG | The overlap extension PCR for inserting *PET8* 5’ UTR into *Xho* *I* site of LHZ1138 |
| OZJY57F | CTTTTGAATGCTCAGACCCCTTTTCATAGTTTTCCATTAGAGAAGTGTATAG | The overlap extension PCR for inserting *TCM62* 5’ UTR into *Xho* *I* site of LHZ1138 |
| OZJY57R | GTGAAAAGTTCTTCTCCTTTACTCATTTTTTCGAAAAGACAATTAGCAATTATGC | The overlap extension PCR for inserting *TCM62* 5’ UTR into *Xho* *I* site of LHZ1138 |
| OZJY58F | CTTTTGAATGCTCAGACCCCGAGATAAGAATCCGAAAAATCTAAAATACAG | The overlap extension PCR for inserting *PTK2* 5’ UTR into *Xho* *I* site of LHZ1138 |
| OZJY58R | GTGAAAAGTTCTTCTCCTTTACTCATTGCGTATGTGTGCTTCCTTTTATC | The overlap extension PCR for inserting *PTK2* 5’ UTR into *Xho* *I* site of LHZ1138 |
| OZJY59F | CTTTTGAATGCTCAGACCCCCGTATTAATTCCATATTTCATCCTTATTTCAATATTAAC | The overlap extension PCR for inserting *USA1* 5’ UTR into *Xho* *I* site of LHZ1138 |
| OZJY59R | GTGAAAAGTTCTTCTCCTTTACTCATGGTGTAGCAAAATGTTTCCAATGATT | The overlap extension PCR for inserting *USA1* 5’ UTR into *Xho* *I* site of LHZ1138 |
| OZJY60F | CTTTTGAATGCTCAGACCCCGACATTATTCTACCAGCTAAAACAGC | The overlap extension PCR for inserting *SSE1* 5’ UTR into *Xho* *I* site of LHZ1138 |
| OZJY60R | GTGAAAAGTTCTTCTCCTTTACTCATCGCTCTTTTTAATATTCTATATTATTACCAGGAG | The overlap extension PCR for inserting *SSE1* 5’ UTR into *Xho* *I* site of LHZ1138 |
| OZJY61F | CTTTTGAATGCTCAGACCCCGCTTAAGATAAAACAAGAGTTCGTATCG | The overlap extension PCR for inserting *ECM14* 5’ UTR into *Xho* *I* site of LHZ1138 |
| OZJY61R | GTGAAAAGTTCTTCTCCTTTACTCATTTAGAGGGTATTTATTACACTAAACTACCCAATC | The overlap extension PCR for inserting *ECM14* 5’ UTR into *Xho* *I* site of LHZ1138 |
| OZJY62F | CTTTTGAATGCTCAGACCCCGCCAGAGCTTTTGTAAGCG | The overlap extension PCR for inserting *CYS3* 5’ UTR into *Xho* *I* site of LHZ1138 |
| OZJY62R | GTGAAAAGTTCTTCTCCTTTACTCATTTTTTCTAGTGTGTCAATTGATGGTTTTTG | The overlap extension PCR for inserting *CYS3* 5’ UTR into *Xho* *I* site of LHZ1138 |
| OZJY63F | CTTTTGAATGCTCAGACCCCAGCCAGAAAAAGAGCTAAAGCAG | The overlap extension PCR for inserting *ISD11* 5’ UTR into *Xho* *I* site of LHZ1138 |
| OZJY63R | GTGAAAAGTTCTTCTCCTTTACTCATATTTCCGTAGTTTAGTGAACTTTTCTTAACAG | The overlap extension PCR for inserting *ISD11* 5’ UTR into *Xho* *I* site of LHZ1138 |
| OZJY64F | ACAGAAAGTTCATTAAAAAATGAGTAAAGGAGAAGAACTTTTCAC | The amplification of *MNP1* 5’ UTR with homologous arms for insertion into *Xho* I site of LHZ1138 |
| OZJY64R | TTTTTAATGAACTTTCTGTGGGGTCTGAGCATTCAAAAG | The amplification of *MNP1* 5’ UTR with homologous arms for insertion into *Xho* I site of LHZ1138 |
| OZJY65F | CTTTTGAATGCTCAGACCCCAAATAAGAGAACAGCGGTAAAGAGAAG | The overlap extension PCR for inserting *HIS2* 5’ UTR into *Xho* *I* site of LHZ1138 |
| OZJY65R | GTGAAAAGTTCTTCTCCTTTACTCATTGCGGATTTAAGTAACTTGCTCG | The overlap extension PCR for inserting *HIS2* 5’ UTR into *Xho* *I* site of LHZ1138 |
| OZJY66F | CTTTTGAATGCTCAGACCCCGCGTATAAAGGATAACAGGGAGTTG | The overlap extension PCR for inserting *RPT6* 5’ UTR into *Xho* *I* site of LHZ1138 |
| OZJY66R | GTGAAAAGTTCTTCTCCTTTACTCATCTCTTATTAATGCTCTTTCCAATTTTTCTAATG | The overlap extension PCR for inserting *RPT6* 5’ UTR into *Xho* *I* site of LHZ1138 |
| OZJY67F | GAGGTTGATATTGATAGGCATTTGTGGGGTGTTAATATTAGGCGTTAAAAAAAGCAGAGAATGAGTAAAGGAGAAGAACTTTTCAC | The amplification of *UTP15* 5’ UTR with homologous arms for insertion into *Xho* I site of LHZ1138 |
| OZJY67R | GCCTATCAATATCAACCTCTATCTACTGGGATTGGCCTTGGGGTCTGAGCATTCAAAAG | The amplification of *UTP15* 5’ UTR with homologous arms for insertion into *Xho* I site of LHZ1138 |
| OZJY68F | CACAGTGCAGAGATGAGTAAAGGAGAAGAACTTTTCAC | The overlap extension PCR for inserting *COX16* 5’ UTR into *Xho* *I* site of LHZ1138 |
| OZJY68R | CTCTGCACTGTGGGGGTCTGAGCATTCAAAAG | The overlap extension PCR for inserting *COX16* 5’ UTR into *Xho* *I* site of LHZ1138 |
| OZJY69F | CTTTTGAATGCTCAGACCCCAGGAATAGGTGGGTGCTCTC | The overlap extension PCR for inserting *MNN10* 5’ UTR into *Xho* *I* site of LHZ1138 |
| OZJY69R | GTGAAAAGTTCTTCTCCTTTACTCATTGCGCCGGTTAAAATATGCTG | The overlap extension PCR for inserting *MNN10* 5’ UTR into *Xho* *I* site of LHZ1138 |
| OZJY70F | CTTTTGAATGCTCAGACCCCATATATGAGTATCAAATAAAAAGTGAACTTTAGTTCC | The overlap extension PCR for inserting *SSB* 5’ UTR into *Xho* *I* site of LHZ1138 |
| OZJY70R | GTGAAAAGTTCTTCTCCTTTACTCATTGTTGGAATATGAATGTAGCTTGACG | The overlap extension PCR for inserting *SSB* 5’ UTR into *Xho* *I* site of LHZ1138 |
| OZJY71F | CTTTTGAATGCTCAGACCCCAAAACCAAATAACCAAGGACTAACTAAC | The overlap extension PCR for inserting *OLE1* 5’ UTR into *Xho* *I* site of LHZ1138 |
| OZJY71R | GTGAAAAGTTCTTCTCCTTTACTCATGGTATTTGTTATGATTATTCCTATTCCTATTC | The overlap extension PCR for inserting *OLE1* 5’ UTR into *Xho* *I* site of LHZ1138 |
| OZJY72F | GTTTATTAGACAGACAGAGAGACAAAGGTTGGCTTTTAGACGAGAGCAAGCAGTATAGAAAAAGATGAGTAAAGGAGAAGAACTTTTCAC | The amplification of *RLI1* 5’ UTR with homologous arms for insertion into *Xho* I site of LHZ1138 |
| OZJY72R | CTCTGTCTGTCTAATAAACGAAAACCCTTTGTAATGTTATTTATCTAAGGATAATTTTATATCAGTGTCTGGGGTCTGAGCATTCAAAAG | The amplification of *RLI1* 5’ UTR with homologous arms for insertion into *Xho* I site of LHZ1138 |
| OZJY73F | CTTTTGAATGCTCAGACCCCAGGAAATACTTAGACCCAGTAACGTG | The overlap extension PCR for inserting *PSY2* 5’ UTR into *Xho* *I* site of LHZ1138 |
| OZJY73R | GTGAAAAGTTCTTCTCCTTTACTCATCGTCAATGCAAACCCACAATATTATAG | The overlap extension PCR for inserting *PSY2* 5’ UTR into *Xho* *I* site of LHZ1138 |
| OZJY74F | AGCGCTCGATTGATTGATTCAGTATGAGTAAAGGAGAAGAACTTTTCAC | The overlap extension PCR for inserting *KLMA*_*20249* 5’ UTR into *Xho* *I* site of LHZ1138 |
| OZJY74R | ACTGAATCAATCAATCGAGCGCTGGGGTCTGAGCATTCAAAAG | The overlap extension PCR for inserting *KLMA*_*20249* 5’ UTR into *Xho* *I* site of LHZ1138 |
| OZJY75F | CTTTTGAATGCTCAGACCCCAGAAACAGAAAATAGACACTTAGTCATATC | The overlap extension PCR for inserting *GAL1* 5’ UTR into *Xho* *I* site of LHZ1138 |
| OZJY75R | TGAAAAGTTCTTCTCCTTTACTCATATTAGCTAGTTAATAGGTATAATAATAATAACAAC | The overlap extension PCR for inserting *GAL1* 5’ UTR into *Xho* *I* site of LHZ1138 |
| OZJY76F | GCGGTAGTGTACGGTGCGATATGAGTAAAGGAGAAGAACTTTTCAC | The overlap extension PCR for inserting *RSM24* 5’ UTR into *Xho* *I* site of LHZ1138 |
| OZJY76R | ATCGCACCGTACACTACCGCGGGGTCTGAGCATTCAAAAG | The overlap extension PCR for inserting *RSM24* 5’ UTR into *Xho* *I* site of LHZ1138 |
| OZJY77F | CTTTTGAATGCTCAGACCCCAGGATCAGGAAAGCCAAAAACAAG | The overlap extension PCR for inserting *FRS2* 5’ UTR into *Xho* *I* site of LHZ1138 |
| OZJY77R | GTGAAAAGTTCTTCTCCTTTACTCATTTTTTATGGATTCTATAACAAGATCTTCGGATTG | The overlap extension PCR for inserting *FRS2* 5’ UTR into *Xho* *I* site of LHZ1138 |
| OZJY78F | CTTTTGAATGCTCAGACCCCACTGCAATTCGGTACGGAG | The overlap extension PCR for inserting *AYR1* 5’ UTR into *Xho* *I* site of LHZ1138 |
| OZJY78R | GTGAAAAGTTCTTCTCCTTTACTCATTGCAGAAATATCCTTTTTTCTTTCACG | The overlap extension PCR for inserting *AYR1* 5’ UTR into *Xho* *I* site of LHZ1138 |
| OZJY79F | GTGGTCGTGCAGGTGATCGATCGAGGATGAGTAAAGGAGAAGAACTTTTCAC | The overlap extension PCR for inserting *LSB6* 5’ UTR into *Xho* *I* site of LHZ1138 |
| OZJY79R | CCTCGATCGATCACCTGCACGACCACGGGGTCTGAGCATTCAAAAG | The overlap extension PCR for inserting *LSB6* 5’ UTR into *Xho* *I* site of LHZ1138 |
| OZJY80F | TTAGCTCGACTCGAGAGAAAGGAAAAAGGGGAAACAACAGAGAGATACAACAATTAAATTGGACATGAGTAAAGGAGAAGAACTTTTCAC | The amplification of *SNF1* 5’ UTR with homologous arms for insertion into *Xho* I site of LHZ1138 |
| OZJY80R | TTTCTCTCGAGTCGAGCTAACTGGTACACCTTTTCTGATTACTCTGCTGTGAGCTAATTCTTCGCTCTGCGGGGTCTGAGCATTCAAAAG | The amplification of *SNF1* 5’ UTR with homologous arms for insertion into *Xho* I site of LHZ1138 |
| OZJY81F | CGGCAGGTGCTTTTATAGATATCACAAAACAAATAAAAAGAAGACATGAGTAAAGGAGAAGAACTTTTCAC | The amplification of *rplM* 5’ UTR with homologous arms for insertion into *Xho* I site of LHZ1138 |
| OZJY81R | ATATCTATAAAAGCACCTGCCGAGTAGAATTTCACTGCTCGGGGTCTGAGCATTCAAAAG | The amplification of *rplM* 5’ UTR with homologous arms for insertion into *Xho* I site of LHZ1138 |
| OZJY82F | CTTTTGAATGCTCAGACCCCGACGCGCTAGCTAGTTTAGG | The overlap extension PCR for inserting *SSA2* 5’ UTR into *Xho* *I* site of LHZ1138 |
| OZJY82R | GTGAAAAGTTCTTCTCCTTTACTCATATTTGCTTAATTTATTAATTGACTTTTTTGAAG | The overlap extension PCR for inserting *SSA2* 5’ UTR into *Xho* *I* site of LHZ1138 |
| OZJY83F | GTCCTGCGGTGACAATTATTTGTGATCTGTGACAAAACACTTCAAAAAAAAATTAGGTTAGGATGAGTAAAGGAGAAGAACTTTTCAC | The amplification of *GCD7* 5’ UTR with homologous arms for insertion into *Xho* I site of LHZ1138 |
| OZJY83R | CAAATAATTGTCACCGCAGGACAATTCGAAAGGGCCTGCTTACAACCTTCCCTGGGGTCTGAGCATTCAAAAG | The amplification of *GCD7* 5’ UTR with homologous arms for insertion into *Xho* I site of LHZ1138 |
| OZJY84F | CTTTTGAATGCTCAGACCCCGAGCTTATAAAGGATAAATATCAATCAGCATCG | The overlap extension PCR for inserting *PHB2* 5’ UTR into *Xho* *I* site of LHZ1138 |
| OZJY84R | GTGAAAAGTTCTTCTCCTTTACTCATTTTATATTAATTTTATTGTATGCGCTGCTTGC | The overlap extension PCR for inserting *PHB2* 5’ UTR into *Xho* *I* site of LHZ1138 |
| OZJY85F | CTTTTGAATGCTCAGACCCCTGGTAGTCGTTTGGGTAGACAGTAG | The overlap extension PCR for inserting *YHM2* 5’ UTR into *Xho* *I* site of LHZ1138 |
| OZJY85R | GTGAAAAGTTCTTCTCCTTTACTCATCCTTATTCTTATTCTCTTTTCCCCTTTTTATTC | The overlap extension PCR for inserting *YHM2* 5’ UTR into *Xho* *I* site of LHZ1138 |
| OZJY86F | CTTTTGAATGCTCAGACCCCAGGATAGGTTATATTTTATTTATTCTCTCCG | The overlap extension PCR for inserting *STR3* 5’ UTR into *Xho* *I* site of LHZ1138 |
| OZJY86R | GTGAAAAGTTCTTCTCCTTTACTCATCTTGATCTTTTAATAAGGCATACAAAC | The overlap extension PCR for inserting *STR3* 5’ UTR into *Xho* *I* site of LHZ1138 |
| OZJY87F | TCCAATTGGTAGGTAATTGAGGTTGAAAAGTTTTAGAGAAGCTTCTTGGACAAAAATTGAGCGATGAGTAAAGGAGAAGAACTTTTCAC | The amplification of *SAC1* 5’ UTR with homologous arms for insertion into *Xho* I site of LHZ1138 |
| OZJY87R | CTTTTCAACCTCAATTACCTACCAATTGGATCCTCTCTAAAGCCCTAAATACTCTTAGTACACGTCCCTGGGGTCTGAGCATTCAAAAG | The amplification of *SAC1* 5’ UTR with homologous arms for insertion into *Xho* I site of LHZ1138 |
| OZJY88F | CTTTTGAATGCTCAGACCCCAAAAGCACTTAGCGCTATCAAGTATC | The overlap extension PCR for inserting *OXA1* 5’ UTR into *Xho* *I* site of LHZ1138 |
| OZJY88R | GTGAAAAGTTCTTCTCCTTTACTCATGGAGCATTTTTTGCTCGCAC | The overlap extension PCR for inserting *OXA1* 5’ UTR into *Xho* *I* site of LHZ1138 |
| OZJY89F | CCCATTAGAATTATAACTGCATTGTACTTGAAATCTGAAGCTCTACAAACAGCAGTATCATAATGAGTAAAGGAGAAGAACTTTTCAC | The amplification of *DBP9* 5’ UTR with homologous arms for insertion into *Xho* I site of LHZ1138 |
| OZJY89R | GCAGTTATAATTCTAATGGGTAACAGATACTTCAAGTTTTTCAAAGCTGGGGTCTGAGCATTCAAAAG | The amplification of *DBP9* 5’ UTR with homologous arms for insertion into *Xho* I site of LHZ1138 |
| OZJY90F | GATCACTTTGTATTTTAAGATCAGCAAAGGCATATTAAACCAAAAAGCGAGTCATTATGAGTAAAGGAGAAGAACTTTTCAC | The amplification of *PLP2* 5’ UTR with homologous arms for insertion into *Xho* I site of LHZ1138 |
| OZJY90R | CTTAAAATACAAAGTGATCTTTACTGGATGTTTTGCAAGCGGGGTCTGAGCATTCAAAAG | The amplification of *PLP2* 5’ UTR with homologous arms for insertion into *Xho* I site of LHZ1138 |
| OZJY91F | CTTTTGAATGCTCAGACCCCGCTTAGGCGTCAGGGTACC | The overlap extension PCR for inserting *ATG27* 5’ UTR into *Xho* *I* site of LHZ1138 |
| OZJY91R | GTGAAAAGTTCTTCTCCTTTACTCATATTTGATTTGATTTGGTTTCAGACTCC | The overlap extension PCR for inserting *ATG27* 5’ UTR into *Xho* *I* site of LHZ1138 |
| OZJY92F | CTTTTGAATGCTCAGACCCCAGAAACAGAAAATAGACACTTAGTCATATC | The overlap extension PCR for inserting *GAL1* 5’ UTR into *Xho* *I* site of LHZ1138 |
| OZJY92R | TGAAAAGTTCTTCTCCTTTACTCATATTAGCTAGTTAATAGGTATAATAATAATAACAAC | The overlap extension PCR for inserting *GAL1* 5’ UTR into *Xho* *I* site of LHZ1138 |
| OZJY93F | GGCAATATATCTTGGATAGAATCGTTGTGTGATATATATTAATTAAAAAGGTCAAAGGCGAAATGAGTAAAGGAGAAGAACTTTTCAC | The amplification of *DUG2* 5’ UTR with homologous arms for insertion into *Xho* I site of LHZ1138 |
| OZJY93R | CAACGATTCTATCCAAGATATATTGCCTCAGTCTGAATTCATAGATTAACAAATAGCTTTGGGGGGTCTGAGCATTCAAAAG | The amplification of *DUG2* 5’ UTR with homologous arms for insertion into *Xho* I site of LHZ1138 |
| OZJY94F | GAGATAAAAAGGATTCAATCAGATCAAATCAAATCAAATCTATCTTCTCAATGAGTAAAGGAGAAGAACTTTTCAC | The amplification of *FIM1*_*2079* 5’ UTR with homologous arms for insertion into *Xho* I site of LHZ1138 |
| OZJY94R | GATCTGATTGAATCCTTTTTATCTCTTCTCTCTGTCGGGGTCTGAGCATTCAAAAG | The amplification of *FIM1*_*2079* 5’ UTR with homologous arms for insertion into *Xho* I site of LHZ1138 |
| OZJY95F | CTTTTGAATGCTCAGACCCCAGGACGGCGAGGCTTTTTGAC | The amplification of *PSE1* 5’ UTR with homologous arms for insertion into *Xho* I site of LHZ1138 |
| OZJY95R | GTGAAAAGTTCTTCTCCTTTACTCATTGCGGTAACTATCTATCCCACACACAATG | The amplification of *PSE1* 5’ UTR with homologous arms for insertion into *Xho* I site of LHZ1138 |
| OZJY96F | CTTTTGAATGCTCAGACCCCGAGTTAAAGAGTTTGAGAAACAGTTCAG | The overlap extension PCR for inserting *CAJ1* 5’ UTR into *Xho* *I* site of LHZ1138 |
| OZJY96R | GTGAAAAGTTCTTCTCCTTTACTCATATTTCCGCACACAAAACCAC | The overlap extension PCR for inserting *CAJ1* 5’ UTR into *Xho* *I* site of LHZ1138 |
| OZJY97F | CTTTTGAATGCTCAGACCCCGTTTCAATTGACTCCGAACAGGTTC | The overlap extension PCR for inserting *ARO1* 5’ UTR into *Xho* *I* site of LHZ1138 |
| OZJY97R | GTGAAAAGTTCTTCTCCTTTACTCATGTTTAAACTTATTGCTTAATCTGCTAAAAAAGC | The overlap extension PCR for inserting *ARO1* 5’ UTR into *Xho* *I* site of LHZ1138 |
| OZJY98F | CTGCAATATCTACCAGAAGTAAAAAAACCGAATAAGCCTCCCCCTACCATGAGTAAAGGAGAAGAACTTTTCAC | The amplification of *AAT2* 5’ UTR with homologous arms for insertion into *Xho* I site of LHZ1138 |
| OZJY98R | ACTTCTGGTAGATATTGCAGGAGTTTGCGATGTTCGGGGTCTGAGCATTCAAAAG | The amplification of *AAT2* 5’ UTR with homologous arms for insertion into *Xho* I site of LHZ1138 |
| OZJY99F | CTTTTGAATGCTCAGACCCCGATTCAGTACTTAAAGTCCTGTTGCAG | The overlap extension PCR for inserting *TPA1* 5’ UTR into *Xho* *I* site of LHZ1138 |
| OZJY99R | GAAAAGTTCTTCTCCTTTACTCATCGCGCTATTACTTTACTTTTCTTTTTTTTTTAAATC | The overlap extension PCR for inserting *TPA1* 5’ UTR into *Xho* *I* site of LHZ1138 |
| OZJY100F | CTTTTGAATGCTCAGACCCCAGTTAGTCTTGGTGTGTTGTAGC | The overlap extension PCR for inserting *PET10* 5’ UTR into *Xho* *I* site of LHZ1138 |
| OZJY100R | GTGAAAAGTTCTTCTCCTTTACTCATTTGGTTCCGTTTTTTCGTTTGG | The overlap extension PCR for inserting *PET10* 5’ UTR into *Xho* *I* site of LHZ1138 |
| OZJY101F | CTTTTGAATGCTCAGACCCCAACCTCAATATAAAAAGAATTGGTAGTTC | The overlap extension PCR for inserting *ADE17* 5’ UTR into *Xho* *I* site of LHZ1138 |
| OZJY101R | TGAAAAGTTCTTCTCCTTTACTCATCTTTTGCTAATATAATACTTTTTATAATTCTCGAG | The overlap extension PCR for inserting *ADE17* 5’ UTR into *Xho* *I* site of LHZ1138 |
| OZJY102F | CTTTTGAATGCTCAGACCCCGTTTAGGTAGTCGCAAGTGTTTTGAAAG | The amplification of *CDC11* 5’ UTR with homologous arms for insertion into *Xho* I site of LHZ1138 |
| OZJY102R | GTGAAAAGTTCTTCTCCTTTACTCATTTCTGTATTCCAATTATAGATCTGTCTTATTAC | The amplification of *CDC11* 5’ UTR with homologous arms for insertion into *Xho* I site of LHZ1138 |
| OZJY103F | CACCAGTAATCCCATAACTAACATCAATTAAAAATGAGTAAAGGAGAAGAACTTTTCAC | The overlap extension PCR for inserting *TSA1* 5’ UTR into *Xho* *I* site of LHZ1138 |
| OZJY103R | GATGTTAGTTATGGGATTACTGGTGTGGTAATTGGGGTCTGAGCATTCAAAAG | The overlap extension PCR for inserting *TSA1* 5’ UTR into *Xho* *I* site of LHZ1138 |
| OZJY104F | CTTTTGAATGCTCAGACCCCAGTTGAAAAATAAGAGTGACCTCCCTC | The overlap extension PCR for inserting *GPX2* 5’ UTR into *Xho* *I* site of LHZ1138 |
| OZJY104R | GTGAAAAGTTCTTCTCCTTTACTCATCTGAATGGTCGATATGATACCAATACG | The overlap extension PCR for inserting *GPX2* 5’ UTR into *Xho* *I* site of LHZ1138 |
| OZJY105F | CTTTTGAATGCTCAGACCCCCAAAAAACACAATTCGACAACCCCAC | The overlap extension PCR for inserting *YAP1* 5’ UTR into *Xho* *I* site of LHZ1138 |
| OZJY105R | GTGAAAAGTTCTTCTCCTTTACTCATCGTGCTAATTCCAATAAGTAGGTCAG | The overlap extension PCR for inserting *YAP1* 5’ UTR into *Xho* *I* site of LHZ1138 |
| OZJY106F | CTTTTGAATGCTCAGACCCCAGGCGTCAATTGAAGAGGC | The overlap extension PCR for inserting *TUP1* 5’ UTR into *Xho* *I* site of LHZ1138 |
| OZJY106R | GTGAAAAGTTCTTCTCCTTTACTCATTATTGTCTGTATTAAATCTTATGTTTTTTGCTTG | The overlap extension PCR for inserting *TUP1* 5’ UTR into *Xho* *I* site of LHZ1138 |
| OZJY107F | CTTTTGAATGCTCAGACCCCGTTAACGGTACGCTGAAAC | The overlap extension PCR for inserting *YPT52* 5’ UTR into *Xho* *I* site of LHZ1138 |
| OZJY107R | GTGAAAAGTTCTTCTCCTTTACTCATTATTAAATTGGAATAACCTTTACTTTATCTTAC | The overlap extension PCR for inserting *YPT52* 5’ UTR into *Xho* *I* site of LHZ1138 |
| OZJY108F | CTTTTGAATGCTCAGACCCCACTGCAATTCGGTACGGAG | The overlap extension PCR for inserting *AYR1* 5’ UTR into *Xho* *I* site of LHZ1138 |
| OZJY108R | GTGAAAAGTTCTTCTCCTTTACTCATTGCAGAAATATCCTTTTTTCTTTCACG | The overlap extension PCR for inserting *AYR1* 5’ UTR into *Xho* *I* site of LHZ1138 |
| OZJY109F | GCAAAAACCAGCTTTCACCGCAGATATCTCACAGAATTAGTAAAAGAAAGACGGGTAGAAAAATGAGTAAAGGAGAAGAACTTTTCAC | The amplification of *CCT6* 5’ UTR with homologous arms for insertion into *Xho* I site of LHZ1138 |
| OZJY109R | GCGGTGAAAGCTGGTTTTTGCTGATAGAGATTCCGAAGAAGAAGGTTTCTGGGGTCTGAGCATTCAAAAG | The amplification of *CCT6* 5’ UTR with homologous arms for insertion into *Xho* I site of LHZ1138 |
| OZJY110F | CTTTTGAATGCTCAGACCCCAGGTTAGGTTTGCTAGGTATCCG | The overlap extension PCR for inserting *PRS3* 5’ UTR into *Xho* *I* site of LHZ1138 |
| OZJY110R | GTGAAAAGTTCTTCTCCTTTACTCATGCTTGTTAAGTTACTAGTAGATCCTAGTTAC | The overlap extension PCR for inserting *PRS3* 5’ UTR into *Xho* *I* site of LHZ1138 |
| OZJY111F | TATATAATAAATAAGGATACGGAGTACAGAACAGCAAGGTCATCAGAAGATAGAGCGTGTCGCCATGAGTAAAGGAGAAGAACTTTTCAC | The amplification of *DPB3* 5’ UTR with homologous arms for insertion into *Xho* I site of LHZ1138 |
| OZJY111R | TATCCTTATTTATTATATAGCACAGTTTTTGGTTATATTTTATCTAGATAACAATGTATGTGCTTTTATTGGGGTCTGAGCATTCAAAAG | The amplification of *DPB3* 5’ UTR with homologous arms for insertion into *Xho* I site of LHZ1138 |
| OZJY112F | CTTTTGAATGCTCAGACCCCGTTGAGAACATCTTATAATCTCTGGAG | The overlap extension PCR for inserting *PAA1* 5’ UTR into *Xho* *I* site of LHZ1138 |
| OZJY112R | GTGAAAAGTTCTTCTCCTTTACTCATGACGATTGATTTCTGATCTATACTAGTCTC | The overlap extension PCR for inserting *PAA1* 5’ UTR into *Xho* *I* site of LHZ1138 |
| OZJY113F | CTTTTGAATGCTCAGACCCCATCTATAACATTAGAATAAGTAGGAGTTAGAAGATTG | The overlap extension PCR for inserting *KLMA*_*40607* 5’ UTR into *Xho* *I* site of LHZ1138 |
| OZJY113R | TGAAAAGTTCTTCTCCTTTACTCATTTTTCAATGTATAATGTTGTCCTGTAATCAATTTC | The overlap extension PCR for inserting *KLMA*_*40607* 5’ UTR into *Xho* *I* site of LHZ1138 |
| OZJY114F | CTTTTGAATGCTCAGACCCCAGGAAACGGGCATCTGAG | The overlap extension PCR for inserting *RPN4* 5’ UTR into *Xho* *I* site of LHZ1138 |
| OZJY114R | GTGAAAAGTTCTTCTCCTTTACTCATCTTATCCTCTTTGCTCCTAATTACTAATG | The overlap extension PCR for inserting *RPN4* 5’ UTR into *Xho* *I* site of LHZ1138 |
| OZJY115F | CTTTTGAATGCTCAGACCCCAGTGTGTGGGATATCACTATCCG | The amplification of *KLMA*_*70208* 5’ UTR with homologous arms for insertion into *Xho* I site of LHZ1138 |
| OZJY115R | GTGAAAAGTTCTTCTCCTTTACTCATTCTCGCACGCTACTCTAAGATACTATCTATAC | The amplification of *KLMA*_*70208* 5’ UTR with homologous arms for insertion into *Xho* I site of LHZ1138 |
| OZJY116F | AAGGCAACCGCAAGTCGAGGCAGCATGAGTAAAGGAGAAGAACTTTTCAC | The overlap extension PCR for inserting *PAM16* 5’ UTR into *Xho* *I* site of LHZ1138 |
| OZJY116R | GCTGCCTCGACTTGCGGTTGCCTTGGGGTCTGAGCATTCAAAAG | The overlap extension PCR for inserting *PAM16* 5’ UTR into *Xho* *I* site of LHZ1138 |
| OZJY117F | GACAAGAGGAGTGTTTAGGGTTTTGCAAAAAGATATAACATCCAAATGAGTAAAGGAGAAGAACTTTTCAC | The amplification of *KLMA*_*80335* 5’ UTR with homologous arms for insertion into *Xho* I site of LHZ1138 |
| OZJY117R | GCAAAACCCTAAACACTCCTCTTGTCCTTCACTGTTCCACGGGGTCTGAGCATTCAAAAG | The amplification of *KLMA*_*80335* 5’ UTR with homologous arms for insertion into *Xho* I site of LHZ1138 |
| OZJY118F | GGAAAGACAATAAAAGGCTTGCATAGTTTTGTCTGAATCAAAATACAAAAAATTAGAAAAGATGAGTAAAGGAGAAGAACTTTTCAC | The amplification of *RTN1* 5’ UTR with homologous arms for insertion into *Xho* I site of LHZ1138 |
| OZJY118R | CTATGCAAGCCTTTTATTGTCTTTCCTTAGTTGCTACGATGAGAACAAAACACACTCTAGTTGGGGTCTGAGCATTCAAAAG | The amplification of *RTN1* 5’ UTR with homologous arms for insertion into *Xho* I site of LHZ1138 |
| OZJY119F | CCGAGGTGGAAACCAGTATAAGACAATAGAGGATAGAGATGAGTAAAGGAGAAGAACTTTTCAC | The amplification of *rplF* 5’ UTR with homologous arms for insertion into *Xho* I site of LHZ1138 |
| OZJY119R | TCTTATACTGGTTTCCACCTCGGAATGTCCTTTTTATGGCGGGGTCTGAGCATTCAAAAG | The amplification of *rplF* 5’ UTR with homologous arms for insertion into *Xho* I site of LHZ1138 |
| OZJY120F | TTCTGGTGGGAATTAGGGTCACAGATCAAAAAAAATGAGTAAAGGAGAAGAACTTTTCAC | The amplification of *KLMA*_*60154* 5’ UTR with homologous arms for insertion into *Xho* I site of LHZ1138 |
| OZJY120R | GACCCTAATTCCCACCAGAATACGCCTCGATTGGTCTCTCGGGGTCTGAGCATTCAAAAG | The amplification of *KLMA*_*60154* 5’ UTR with homologous arms for insertion into *Xho* I site of LHZ1138 |
| OZJY121F | ACAGACTTAGGTAGGAACAGAAAGGGATAAAAGTTTCTTTAGGACCAATTGGATAGAATTTAGGATGAGTAAAGGAGAAGAACTTTTCAC | The amplification of *GRE2* 5’ UTR with homologous arms for insertion into *Xho* I site of LHZ1138 |
| OZJY121R | TCTGTTCCTACCTAAGTCTGTTAGATAAAGGTTATATCTAGAAATAGGGTTATATTTCTCTACCTTTTTTGGGGTCTGAGCATTCAAAAG | The amplification of *GRE2* 5’ UTR with homologous arms for insertion into *Xho* I site of LHZ1138 |
| OZJY122F | GTAGGGTACCAGAAAGTTGGTGTTGATAACATATTTGAAGGAGAAACAGAGAAAAAAGCGGCGATGAGTAAAGGAGAAGAACTTTTCAC | The amplification of *KLMA*_*60072* 5’ UTR with homologous arms for insertion into *Xho* I site of LHZ1138 |
| OZJY122R | CACCAACTTTCTGGTACCCTACACACTACCAAACCAGTACGAAAAGCACTATAGTACCCTGTTTCCCCGGGGTCTGAGCATTCAAAAG | The amplification of *KLMA*_*60072* 5’ UTR with homologous arms for insertion into *Xho* I site of LHZ1138 |
| OZJY123F | CGCCAAACACCAAGAATTCCCCATAAAAAGACATGAGTAAAGGAGAAGAACTTTTCAC | The amplification of *COX23* 5’ UTR with homologous arms for insertion into *Xho* I site of LHZ1138 |
| OZJY123R | CTTTTTATGGGGAATTCTTGGTGTTTGGCGCGGGGTCTGAGCATTCAAAAG | The amplification of *COX23* 5’ UTR with homologous arms for insertion into *Xho* I site of LHZ1138 |
| OZJY124F | GCCGCCACTTAGCATACACGTATCATAATGAGTAAAGGAGAAGAACTTTTCAC | The overlap extension PCR for inserting *ATP7* 5’ UTR into *Xho* *I* site of LHZ1138 |
| OZJY124R | TATGATACGTGTATGCTAAGTGGCGGCGGGGTCTGAGCATTCAAAAG | The overlap extension PCR for inserting *ATP7* 5’ UTR into *Xho* *I* site of LHZ1138 |
| OZJY125F | CTTTTGAATGCTCAGACCCCATTGTTGGAAAGAGGAGAAGTCTAC | The overlap extension PCR for inserting *KLMA*_*70011* 5’ UTR into *Xho* *I* site of LHZ1138 |
| OZJY125R | TGAAAAGTTCTTCTCCTTTACTCATGTTCCTTAGTATTTTAACTCAAACTTATCTTTTTC | The overlap extension PCR for inserting *KLMA*_*70011* 5’ UTR into *Xho* *I* site of LHZ1138 |
| OZJY126F | GACTAGGGGCTACCAGAGACGCAAGCGAGAAATTCCAGAGAAAAAATGAGTAAAGGAGAAGAACTTTTCAC | The amplification of *LIA1* 5’ UTR with homologous arms for insertion into *Xho* I site of LHZ1138 |
| OZJY126R | GCTTGCGTCTCTGGTAGCCCCTAGTCCTGATGTCACACTTGGGGTCTGAGCATTCAAAAG | The amplification of *LIA1* 5’ UTR with homologous arms for insertion into *Xho* I site of LHZ1138 |
| OZJY127F | CTTTTGAATGCTCAGACCCCAGGCATATATTGTAGAGGATTGCTTG | The overlap extension PCR for inserting *VMA2* 5’ UTR into *Xho* *I* site of LHZ1138 |
| OZJY127R | GTGAAAAGTTCTTCTCCTTTACTCATATCTGTCTAATCTATGCTCTTCGCTTTTTTTTC | The overlap extension PCR for inserting *VMA2* 5’ UTR into *Xho* *I* site of LHZ1138 |
| OZJY128F | ATCTTCGAGTCGATATTGCATATAATCTCCAAATGAGTAAAGGAGAAGAACTTTTCAC | The amplification of *TRL1* 5’ UTR with homologous arms for insertion into *Xho* I site of LHZ1138 |
| OZJY128R | TTATATGCAATATCGACTCGAAGATTTTTCTATGCTACTGGGGTCTGAGCATTCAAAAG | The amplification of *TRL1* 5’ UTR with homologous arms for insertion into *Xho* I site of LHZ1138 |
| OZJY129F | GAAAAAATAAATTAAAACAAAAGTATTATAGCAATAGTGTGAAATTATTGTAAAGATGAGTAAAGGAGAAGAACTTTTCAC | The amplification of *PHM7* 5’ UTR with homologous arms for insertion into *Xho* I site of LHZ1138 |
| OZJY129R | GCTATAATACTTTTGTTTTAATTTATTTTTTCAGTTTATAGACAGATGCTTGGGGTCTGAGCATTCAAAAG | The amplification of *PHM7* 5’ UTR with homologous arms for insertion into *Xho* I site of LHZ1138 |
| OZJY130F | CTTTTGAATGCTCAGACCCCGCACCAACCATCCTCTCAAAC | The overlap extension PCR for inserting *VPS74* 5’ UTR into *Xho* *I* site of LHZ1138 |
| OZJY130R | GTGAAAAGTTCTTCTCCTTTACTCATCGTGCTATATATAAAGTATTACCCTTTTTTC | The overlap extension PCR for inserting *VPS74* 5’ UTR into *Xho* *I* site of LHZ1138 |
| OZJY131F | AGGATAATAACCGAAATAGTACTTTGTTGATTTTATTAGTTTGTTGTTAATAGCCAAATTCAAGATGAGTAAAGGAGAAGAACTTTTCAC | The amplification of *GCD11* 5’ UTR with homologous arms for insertion into *Xho* I site of LHZ1138 |
| OZJY131R | GTACTATTTCGGTTATTATCCTTTTTTAAGTACTTTCCCAAGAATTACAACGCCTTGCCGTTCTGTCTTCGGGGTCTGAGCATTCAAAAG | The amplification of *GCD11* 5’ UTR with homologous arms for insertion into *Xho* I site of LHZ1138 |
| OZJY132F | CTTTTGAATGCTCAGACCCCAAGGTACAAACTTTAAAGATAGCAAGAAG | The overlap extension PCR for inserting *MTR2* 5’ UTR into *Xho* *I* site of LHZ1138 |
| OZJY132R | GTGAAAAGTTCTTCTCCTTTACTCATCTTGTAAACTTTTTAGATATTAAAAGGTCTGC | The overlap extension PCR for inserting *MTR2* 5’ UTR into *Xho* *I* site of LHZ1138 |
| OZJY133F | GGTATCTTTTTTGTTAGTTTCCATTTTTATAGAATGAGTAAAGGAGAAGAACTTTTCAC | The overlap extension PCR for inserting *RPB11* 5’ UTR into *Xho* *I* site of LHZ1138 |
| OZJY133R | GGAAACTAACAAAAAAGATACCCTGGGATCCCGTGTCTGGGGTCTGAGCATTCAAAAG | The overlap extension PCR for inserting *RPB11* 5’ UTR into *Xho* *I* site of LHZ1138 |
| OZJY134F | CACATCCCCAATTGAATTGAACAAGTATTACTCATAGAATCCCAATAATGAGTAAAGGAGAAGAACTTTTCAC | The overlap extension PCR for inserting *HSP12* 5’ UTR into *Xho* *I* site of LHZ1138 |
| OZJY134R | GTTCAATTCAATTGGGGATGTGTTTTAAGTGTTAATTGCAACGCTTAAACTACCTTGATTGTTGGGGTCTGAGCATTCAAAAG | The overlap extension PCR for inserting *HSP12* 5’ UTR into *Xho* *I* site of LHZ1138 |
| OZJY135F | GGAAGTATCTACCCTTCTTGCAACAATCCGTATCGCCATGAGTAAAGGAGAAGAACTTTTCAC | The overlap extension PCR for inserting *GUA1* 5’ UTR into *Xho* *I* site of LHZ1138 |
| OZJY135R | CAAGAAGGGTAGATACTTCCAAAGAAACAAGTTGTTACGGGGTCTGAGCATTCAAAAG | The overlap extension PCR for inserting *GUA1* 5’ UTR into *Xho* *I* site of LHZ1138 |
| OZJY136F | AAAAATCAAGAAATCAACCACATCTAATAAAACATGAGTAAAGGAGAAGAACTTTTCAC | The amplification of *FPR3* 5’ UTR with homologous arms for insertion into *Xho* I site of LHZ1138 |
| OZJY136R | TTATTAGATGTGGTTGATTTCTTGATTTTTAGTCGTCGGGGTCTGAGCATTCAAAAG | The amplification of *FPR3* 5’ UTR with homologous arms for insertion into *Xho* I site of LHZ1138 |
| OZJY137F | CTTTTGAATGCTCAGACCCCGCTTCACAACTGACCAATTAATCC | The overlap extension PCR for inserting *ERV25* 5’ UTR into *Xho* *I* site of LHZ1138 |
| OZJY137R | GTGAAAAGTTCTTCTCCTTTACTCATCTAGACTCTTCTTTTTATGCAAACTGTAG | The overlap extension PCR for inserting *ERV25* 5’ UTR into *Xho* *I* site of LHZ1138 |
| OZJY138F | CTTATATATCGTATTATACTAGACCGGGACCATGAGTAAAGGAGAAGAACTTTTCAC | The overlap extension PCR for inserting *RPC82* 5’ UTR into *Xho* *I* site of LHZ1138 |
| OZJY138R | CTAGTATAATACGATATATAAGTACACTCGCCGCCGCTGGATCCTCTGGGGTCTGAGCATTCAAAAG | The overlap extension PCR for inserting *RPC82* 5’ UTR into *Xho* *I* site of LHZ1138 |
| OZJY139F | CTTTTGAATGCTCAGACCCCAAGGGAAAGAATCTTATAACGTATATTTACTG | The overlap extension PCR for inserting *DPM1* 5’ UTR into *Xho* *I* site of LHZ1138 |
| OZJY139R | GTGAAAAGTTCTTCTCCTTTACTCATGTTGTTTTTTGTTTTATTTTTAGTTATAGTAGTAATG | The overlap extension PCR for inserting *DPM1* 5’ UTR into *Xho* *I* site of LHZ1138 |
| OZJY140F | CTATTTTAGCGACCTTAGGAGAAAATACGAAAGTAGGACTACAATTGACAAAAAATGAGTAAAGGAGAAGAACTTTTCAC | The amplification of *CLC1* 5’ UTR with homologous arms for insertion into *Xho* I site of LHZ1138 |
| OZJY140R | CCTAAGGTCGCTAAAATAGGATCCTAAAAGGCTGCACTTGGGGTCTGAGCATTCAAAAG | The amplification of *CLC1* 5’ UTR with homologous arms for insertion into *Xho* I site of LHZ1138 |
| OZJY141F | AAGGAAAAAAAAGGCAAATGAGTAAAGGAGAAGAACTTTTCAC | The amplification of *fabG* 5’ UTR with homologous arms for insertion into *Xho* I site of LHZ1138 |
| OZJY141R | TTGCCTTTTTTTTCCTTGGGGTCTGAGCATTCAAAAG | The amplification of *fabG* 5’ UTR with homologous arms for insertion into *Xho* I site of LHZ1138 |
| OZJY142F | GAAACCAAGTCACACTAAAAAAATACTTCAGCAATGAGTAAAGGAGAAGAACTTTTCAC | The amplification of *FMP37* 5’ UTR with homologous arms for insertion into *Xho* I site of LHZ1138 |
| OZJY142R | TGCTGAAGTATTTTTTTAGTGTGACTTGGTTTCCGATCGGGGTCTGAGCATTCAAAAG | The amplification of *FMP37* 5’ UTR with homologous arms for insertion into *Xho* I site of LHZ1138 |
| OZJY143F | CTTTTGAATGCTCAGACCCCAGGATAAAAAACTCTTCAGCTGTTG | The overlap extension PCR for inserting *COR1* 5’ UTR into *Xho* *I* site of LHZ1138 |
| OZJY143R | GTGAAAAGTTCTTCTCCTTTACTCATTGTATATGGAAGATCTGTGATAATATATGTGGTG | The overlap extension PCR for inserting *COR1* 5’ UTR into *Xho* *I* site of LHZ1138 |
| OZJY144F | GAACAAAGACAAAAAAAATGAGTAAAGGAGAAGAACTTTTCAC | The amplification of *TMA22* 5’ UTR with homologous arms for insertion into *Xho* I site of LHZ1138 |
| OZJY144R | TTTTTTTGTCTTTGTTCGGGGTCTGAGCATTCAAAAG | The amplification of *TMA22* 5’ UTR with homologous arms for insertion into *Xho* I site of LHZ1138 |
| OZJY145F | GGGCTGATTTGGACATATAGATATAGAGGAGATGAGTAAAGGAGAAGAACTTTTCAC | The overlap extension PCR for inserting *CMD1* 5’ UTR into *Xho* *I* site of LHZ1138 |
| OZJY145R | CTATATGTCCAAATCAGCCCTTTAATGCCCCTAATCTCCTTTCCTTTCTGGGGTCTGAGCATTCAAAAG | The overlap extension PCR for inserting *CMD1* 5’ UTR into *Xho* *I* site of LHZ1138 |
| OZJY146F | CTTTTGAATGCTCAGACCCCGTGCGAGTACGAACTACGAAG | The overlap extension PCR for inserting *SXM1* 5’ UTR into *Xho* *I* site of LHZ1138 |
| OZJY146R | TGAAAAGTTCTTCTCCTTTACTCATGCCGTTTTAGTTAATTAAATCCAATGAAAAAATAC | The overlap extension PCR for inserting *SXM1* 5’ UTR into *Xho* *I* site of LHZ1138 |
| OZJY147F | CTTTTGAATGCTCAGACCCCAGTACACAATACAATAGTTGAATTGAAAC | The overlap extension PCR for inserting *COX5A* 5’ UTR into *Xho* *I* site of LHZ1138 |
| OZJY147R | GTGAAAAGTTCTTCTCCTTTACTCATTGTTTGTTTTATTTTATTGTATCTTGGTG | The overlap extension PCR for inserting *COX5A* 5’ UTR into *Xho* *I* site of LHZ1138 |
| OZJY148F | CTGCTTGTTTGAACTTCGAACTTTGGACTTTGGCCTAAAAATTACATACGGAAGAAATAAAAGATGAGTAAAGGAGAAGAACTTTTCAC | The amplification of *LAT1* 5’ UTR with homologous arms for insertion into *Xho* I site of LHZ1138 |
| OZJY148R | GTTCGAAGTTCAAACAAGCAGATAAGATAAAACAAACTTGAACAACGTGTGTTTAAAACCTCTCAATCGGGGTCTGAGCATTCAAAAG | The amplification of *LAT1* 5’ UTR with homologous arms for insertion into *Xho* I site of LHZ1138 |
| OZJY149F | CTTTTGAATGCTCAGACCCCAGAGTTAATTTAGTGAAAGGGATTGCC | The overlap extension PCR for inserting *ATP1* 5’ UTR into *Xho* *I* site of LHZ1138 |
| OZJY149R | TGAAAAGTTCTTCTCCTTTACTCATTGTTGTATTACTTTATCTTTTTACTTTCCTTTGTG | The overlap extension PCR for inserting *ATP1* 5’ UTR into *Xho* *I* site of LHZ1138 |
| OZJY150F | CTTTTGAATGCTCAGACCCCAGTCTGGTTCTGGAAGGTGAG | The overlap extension PCR for inserting *RIB7* 5’ UTR into *Xho* *I* site of LHZ1138 |
| OZJY150R | GTGAAAAGTTCTTCTCCTTTACTCATCTTCACCCTGTGTGTATGTGTCC | The overlap extension PCR for inserting *RIB7* 5’ UTR into *Xho* *I* site of LHZ1138 |
| OZJY151F | CTTTTGAATGCTCAGACCCCAAGCAATCCAATTCATTACTTGTGC | The overlap extension PCR for inserting *PDI1* 5’ UTR into *Xho* *I* site of LHZ1138 |
| OZJY151R | GTGAAAAGTTCTTCTCCTTTACTCATCCTTTATTATTTTTCGTCTATGGGTTTC | The overlap extension PCR for inserting *PDI1* 5’ UTR into *Xho* *I* site of LHZ1138 |
| OZJY152F | GAAAAAGGTTTGCTTGTGAGCAATAGCGGATCGAATAGGGTTCGAGTGTTTTTGCTATTGAGATGAGTAAAGGAGAAGAACTTTTCAC | The amplification of *YKT6* 5’ UTR with homologous arms for insertion into *Xho* I site of LHZ1138 |
| OZJY152R | CTATTGCTCACAAGCAAACCTTTTTCTTCCTTTCTACCCACTGGAAAAAAAAAACCAGTCAGCTTCGGGGTCTGAGCATTCAAAAG | The amplification of *YKT6* 5’ UTR with homologous arms for insertion into *Xho* I site of LHZ1138 |
| OZJY153F | CTTTTGAATGCTCAGACCCCAGAAGATTGTATAAATAGAGGTGCG | The overlap extension PCR for inserting *GLC3* 5’ UTR into *Xho* *I* site of LHZ1138 |
| OZJY153R | GTGAAAAGTTCTTCTCCTTTACTCATTTTGACTTTTTTTCTGTTACGTGATAC | The overlap extension PCR for inserting *GLC3* 5’ UTR into *Xho* *I* site of LHZ1138 |
| OZJY154F | CTTTTGAATGCTCAGACCCCGACCTTTTATTTAATTCATCTTCTTGTATTG | The overlap extension PCR for inserting *RAG5* 5’ UTR into *Xho* *I* site of LHZ1138 |
| OZJY154R | GTGAAAAGTTCTTCTCCTTTACTCATTTTTGTAAGTGTGTGTGTGTGC | The overlap extension PCR for inserting *RAG5* 5’ UTR into *Xho* *I* site of LHZ1138 |
| OZJY155F | CTTTTGAATGCTCAGACCCCAAGGAGTTTCTCAATTTCAGCTC | The overlap extension PCR for inserting *ERG10* 5’ UTR into *Xho* *I* site of LHZ1138 |
| OZJY155R | GTGAAAAGTTCTTCTCCTTTACTCATCTTCGAAAGTTATGGTATGTTTTTAGTC | The overlap extension PCR for inserting *ERG10* 5’ UTR into *Xho* *I* site of LHZ1138 |
| OZJY156F | CTAAGGAAACGGGTGGAATCGTGAGCAAACGTTCAAACACATAGAAAAAAAGAAAATAAAAGCAATGAGTAAAGGAGAAGAACTTTTCAC | The amplification of *ARC19* 5’ UTR with homologous arms for insertion into *Xho* I site of LHZ1138 |
| OZJY156R | GCTCACGATTCCACCCGTTTCCTTAGTAGTAGTAATCACTCCAGTACTTGGTAATTTGCAGGCTGGTCGGGGTCTGAGCATTCAAAAG | The amplification of *ARC19* 5’ UTR with homologous arms for insertion into *Xho* I site of LHZ1138 |
| OZJY157F | CAAAATATTGGTGTTGAATAGTAAAGAAGCAAAAACAAATCCATCTATGAGTAAAGGAGAAGAACTTTTCAC | The amplification of *MOS1* 5’ UTR with homologous arms for insertion into *Xho* I site of LHZ1138 |
| OZJY157R | CTTTACTATTCAACACCAATATTTTGGCAGCTGGATATCGGGGTCTGAGCATTCAAAAG | The amplification of *MOS1* 5’ UTR with homologous arms for insertion into *Xho* I site of LHZ1138 |
| OZJY158F | GTCGCTAAACACCACAAGTTACGTCAAAAAAAATGAGTAAAGGAGAAGAACTTTTCAC | The amplification of *KLMA*_*50074* 5’ UTR with homologous arms for insertion into *Xho* I site of LHZ1138 |
| OZJY158R | GACGTAACTTGTGGTGTTTAGCGACGTTTCTGGGGTCTGAGCATTCAAAAG | The amplification of *KLMA*_*50074* 5’ UTR with homologous arms for insertion into *Xho* I site of LHZ1138 |
| OZJY159F | CTGTGACCGCTAGCAGTGGATAAAAACGTTGGATATGAGTAAAGGAGAAGAACTTTTCAC | The amplification of *ATG12* 5’ UTR with homologous arms for insertion into *Xho* I site of LHZ1138 |
| OZJY159R | TTTTATCCACTGCTAGCGGTCACAGAGGCTCTTCATCCACGGGGTCTGAGCATTCAAAAG | The amplification of *ATG12* 5’ UTR with homologous arms for insertion into *Xho* I site of LHZ1138 |
| OZJY160F | GACTGTTTTTTTTGCAGCTTAGAGCAGTATAGAAGGTAAAATAAAAAAAAGCCATTAATAATATGAGTAAAGGAGAAGAACTTTTCAC | The amplification of *THR4* 5’ UTR with homologous arms for insertion into *Xho* I site of LHZ1138 |
| OZJY160R | CTGCTCTAAGCTGCAAAAAAAACAGTCTAGAGATTTACTGAGTTTTATTGCTTATTGCGTTCTATGAGCAGGGGTCTGAGCATTCAAAAG | The amplification of *THR4* 5’ UTR with homologous arms for insertion into *Xho* I site of LHZ1138 |
| OZJY161F | GCAACGCACAGTGGGGAAACTCGTTAAAGATAAAATAAAAGGAAAAAATGAGTAAAGGAGAAGAACTTTTCAC | The amplification of *BFR1* 5’ UTR with homologous arms for insertion into *Xho* I site of LHZ1138 |
| OZJY161R | GTTTCCCCACTGTGCGTTGCAGGTATGCTGCTGTGACTTGGGGGTCTGAGCATTCAAAAG | The amplification of *BFR1* 5’ UTR with homologous arms for insertion into *Xho* I site of LHZ1138 |
| OZJY162F | CTTTTGAATGCTCAGACCCCGCGACAGCATCATTATAACCTTAAC | The overlap extension PCR for inserting *PEP4* 5’ UTR into *Xho* *I* site of LHZ1138 |
| OZJY162R | GTGAAAAGTTCTTCTCCTTTACTCATTTTACAATAAAGTTAATATTAATTTGATATTAGCAAGGC | The overlap extension PCR for inserting *PEP4* 5’ UTR into *Xho* *I* site of LHZ1138 |
| OZJY163F | CTTTTGAATGCTCAGACCCCGATCTTTAAAAATACAAAGTACAACGGC | The overlap extension PCR for inserting *PRO1* 5’ UTR into *Xho* *I* site of LHZ1138 |
| OZJY163R | GTGAAAAGTTCTTCTCCTTTACTCATTTTATTCCGTTTACTGCTCTTGAAATTG | The overlap extension PCR for inserting *PRO1* 5’ UTR into *Xho* *I* site of LHZ1138 |
| OZJY164F | CTTTTGAATGCTCAGACCCCAGTTTTGATCTCTTGCTGGAATAGATAGATTC | The amplification of *HYP2* 5’ UTR with homologous arms for insertion into *Xho* I site of LHZ1138 |
| OZJY164R | GTGAAAAGTTCTTCTCCTTTACTCATGTTTGTTGTGTTGTGTTGTATGTGTTGTG | The amplification of *HYP2* 5’ UTR with homologous arms for insertion into *Xho* I site of LHZ1138 |
| OZJY165F | TTAAGTGCTTTATTGAACAGCAACAGGTAGGTGGAAGAAAGAAAAAAAAAGCAAGAAGTAGAAAATGAGTAAAGGAGAAGAACTTTTCAC | The amplification of *ADY2* 5’ UTR with homologous arms for insertion into *Xho* I site of LHZ1138 |
| OZJY165R | GCTGTTCAATAAAGCACTTAAAGGGATGTGTCTGTGTATACTTCGGGGTCTGAGCATTCAAAAG | The amplification of *ADY2* 5’ UTR with homologous arms for insertion into *Xho* I site of LHZ1138 |
| OZJY166F | GCAGCTGCTAGTGCAATATAGAATAAGAAAGCTCTCAATAAATCAGTTAAAAAAATGAGTAAAGGAGAAGAACTTTTCAC | The amplification of *KLMA*_*30365* 5’ UTR with homologous arms for insertion into *Xho* I site of LHZ1138 |
| OZJY166R | TATATTGCACTAGCAGCTGCTTTGTATCTATATGTAAGCCGGGGTCTGAGCATTCAAAAG | The amplification of *KLMA*_*30365* 5’ UTR with homologous arms for insertion into *Xho* I site of LHZ1138 |
| OZJY167F | GAATAATCCAATAGGTAATTATTTGCAAGATGAGTAAAGGAGAAGAACTTTTCAC | The overlap extension PCR for inserting *IDP1* 5’ UTR into *Xho* *I* site of LHZ1138 |
| OZJY167R | CAAATAATTACCTATTGGATTATTCTAAAGGTGCATGCAACTCACTGCCTGGGGTCTGAGCATTCAAAAG | The overlap extension PCR for inserting *IDP1* 5’ UTR into *Xho* *I* site of LHZ1138 |
| OZJY168F | GAAAAATCGCAACCATCAGCAACCGCCATCATGAGTAAAGGAGAAGAACTTTTCAC | The amplification of *GNT1*-*B* 5’ UTR with homologous arms for insertion into *Xho* I site of LHZ1138 |
| OZJY168R | GGCGGTTGCTGATGGTTGCGATTTTTCTGGGGTCTGAGCATTCAAAAG | The amplification of *GNT1*-*B* 5’ UTR with homologous arms for insertion into *Xho* I site of LHZ1138 |
| OZJY169F | GCGAAGCTTTGGTGAAGGTTAATTGATAGCAGGGAAACTGGAGATTAGAATGAGTAAAGGAGAAGAACTTTTCAC | The amplification of *AIP1* 5’ UTR with homologous arms for insertion into *Xho* I site of LHZ1138 |
| OZJY169R | TAACCTTCACCAAAGCTTCGCAAGTGCTTTTTTATCGCGGGGTCTGAGCATTCAAAAG | The amplification of *AIP1* 5’ UTR with homologous arms for insertion into *Xho* I site of LHZ1138 |
| OZJY170F | CTTTTGAATGCTCAGACCCCGTATACCTTTTATATAAGAGGAATCGAAAAAG | The overlap extension PCR for inserting *LEU4* 5’ UTR into *Xho* *I* site of LHZ1138 |
| OZJY170R | GTGAAAAGTTCTTCTCCTTTACTCATAGTGTTTCCAGATTGATGTATATATAAATATG | The overlap extension PCR for inserting *LEU4* 5’ UTR into *Xho* *I* site of LHZ1138 |
| OZJY171F | CAAGTGATAGATTAGAACAAAGATTATTTAAAATGAGTAAAGGAGAAGAACTTTTCAC | The overlap extension PCR for inserting *VMA10* 5’ UTR into *Xho* *I* site of LHZ1138 |
| OZJY171R | CTTTGTTCTAATCTATCACTTGTGGAGTTTTGATGCGTGACAGTAACGGGGTCTGAGCATTCAAAAG | The overlap extension PCR for inserting *VMA10* 5’ UTR into *Xho* *I* site of LHZ1138 |
| OZJY172F | CTTTTGAATGCTCAGACCCCAGATAACTTTGTAGATCTGTGTGAGTTAG | The overlap extension PCR for inserting *RIP1* 5’ UTR into *Xho* *I* site of LHZ1138 |
| OZJY172R | GTGAAAAGTTCTTCTCCTTTACTCATTGTATTATTTATGTGTTATTAATGCTTTCTTTTG | The overlap extension PCR for inserting *RIP1* 5’ UTR into *Xho* *I* site of LHZ1138 |
| OZJY173F | CTTTTGAATGCTCAGACCCCAGACAATCACAAATCCGTGTAAATATTCAAAAAG | The overlap extension PCR for inserting *RPT3* 5’ UTR into *Xho* *I* site of LHZ1138 |
| OZJY173R | TGAAAAGTTCTTCTCCTTTACTCATATCAAATGTAATTTATTGTCTTTGTTATATCCTTG | The overlap extension PCR for inserting *RPT3* 5’ UTR into *Xho* *I* site of LHZ1138 |
| OZJY174F | CTATACCCTTGACACATCATCACTCACACAAAAAAAACCGTACGCAATGAGTAAAGGAGAAGAACTTTTCAC | The amplification of *PYK1* 5’ UTR with homologous arms for insertion into *Xho* I site of LHZ1138 |
| OZJY174R | GAGTGATGATGTGTCAAGGGTATAGAGAATATCTTCCTTGGGGTCTGAGCATTCAAAAG | The amplification of *PYK1* 5’ UTR with homologous arms for insertion into *Xho* I site of LHZ1138 |
| OZJY175F | TTAAATAGACACTAGACACCTAGGTTTACATTGCCACACACTTTCAGACAAAAATTTGCAGCAATGAGTAAAGGAGAAGAACTTTTCAC | The amplification of *YVH1* 5’ UTR with homologous arms for insertion into *Xho* I site of LHZ1138 |
| OZJY175R | GGTGTCTAGTGTCTATTTAACAGCCAACTTCGTTGATCGGGGTCTGAGCATTCAAAAG | The amplification of *YVH1* 5’ UTR with homologous arms for insertion into *Xho* I site of LHZ1138 |
| OZJY176F | GACCACTACTAGCTAGTTAATTCAAGAAGCTGAAGTTAAATAAAACAAAAAGAAGAAGCAAAAATGAGTAAAGGAGAAGAACTTTTCAC | The amplification of *KLMA*_*40364* 5’ UTR with homologous arms for insertion into *Xho* I site of LHZ1138 |
| OZJY176R | GAATTAACTAGCTAGTAGTGGTCAATTATGTGCTTCTTGATCCCTGTGCAGATGCTACGAAAATTGTACGGGGTCTGAGCATTCAAAAG | The amplification of *KLMA*_*40364* 5’ UTR with homologous arms for insertion into *Xho* I site of LHZ1138 |
| OZJY177F | AATTCATTAAGAATTAATAGGTAAAAAAAAAAAATGAGTAAAGGAGAAGAACTTTTCAC | The amplification of *HGH1* 5’ UTR with homologous arms for insertion into *Xho* I site of LHZ1138 |
| OZJY177R | TTACCTATTAATTCTTAATGAATTTTAAATTCCAACTCTGGGGTCTGAGCATTCAAAAG | The amplification of *HGH1* 5’ UTR with homologous arms for insertion into *Xho* I site of LHZ1138 |
| OZJY178F | GGTGGTGGTCAGAAAAAGAGATAGAATAATCAATCGTGAAACATGAGTAAAGGAGAAGAACTTTTCAC | The amplification of *STE50* 5’ UTR with homologous arms for insertion into *Xho* I site of LHZ1138 |
| OZJY178R | CTTTTTCTGACCACCACCTTATGGAACGATATTGATCCATGGGGTCTGAGCATTCAAAAG | The amplification of *STE50* 5’ UTR with homologous arms for insertion into *Xho* I site of LHZ1138 |
| OZJY179F | GGAGGACACATTAAGAGCAGTTGATCAATAATTGGCGAAAAGGAAAAAAATGAGTAAAGGAGAAGAACTTTTCAC | The amplification of *KLMA*_*50171* 5’ UTR with homologous arms for insertion into *Xho* I site of LHZ1138 |
| OZJY179R | CTGCTCTTAATGTGTCCTCCTAACCAATCTTCTCGGCCGGGGTCTGAGCATTCAAAAG | The amplification of *KLMA*_*50171* 5’ UTR with homologous arms for insertion into *Xho* I site of LHZ1138 |
| OZJY180F | CGGTCTGGAACTAAACATCGATCAAAGTGGACAATAAAAAGAAAATGAGTAAAGGAGAAGAACTTTTCAC | The amplification of *FIM1*_*2625* 5’ UTR with homologous arms for insertion into *Xho* I site of LHZ1138 |
| OZJY180R | CGATGTTTAGTTCCAGACCGGTTCGATACTTCTTCCCTTGGGGTCTGAGCATTCAAAAG | The amplification of *FIM1*_*2625* 5’ UTR with homologous arms for insertion into *Xho* I site of LHZ1138 |
| OZJY181F | GATACAGCGGCAGAAAAACTCAAACTAGAAGACAACTGCGAAAAGATGAGTAAAGGAGAAGAACTTTTCAC | The amplification of *ARB1* 5’ UTR with homologous arms for insertion into *Xho* I site of LHZ1138 |
| OZJY181R | GAGTTTTTCTGCCGCTGTATCTATATTACTATGATACGGGGTCTGAGCATTCAAAAG | The amplification of *ARB1* 5’ UTR with homologous arms for insertion into *Xho* I site of LHZ1138 |
| OZJY182F | TGATATACATATATATCCCTAATAATTGCTTTAAACGACAACAGAAAACAAATATTTGAAACAATGAGTAAAGGAGAAGAACTTTTCAC | The amplification of *SOU1* 5’ UTR with homologous arms for insertion into *Xho* I site of LHZ1138 |
| OZJY182R | ATTAGGGATATATATGTATATCAATGCTTACGATTGATTTGCCTTGAATTTGCTGTACTATGGTTTTTTCGGGGTCTGAGCATTCAAAAG | The amplification of *SOU1* 5’ UTR with homologous arms for insertion into *Xho* I site of LHZ1138 |
| OZJY183F | CTTTTGAATGCTCAGACCCCAGATATTAAACTGCATTTCACTAGGTAG | The overlap extension PCR for inserting *KLMA*_*60098* 5’ UTR into *Xho* *I* site of LHZ1138 |
| OZJY183R | TGAAAAGTTCTTCTCCTTTACTCATTGGATATAGCTATGGTATCAGAATGATTAATATAG | The overlap extension PCR for inserting *KLMA*_*60098* 5’ UTR into *Xho* *I* site of LHZ1138 |
| OZJY184F | CTATTACCAATTGAGAATAAACACCACCAAACGATACAAGAATGAGTAAAGGAGAAGAACTTTTCAC | The overlap extension PCR for inserting *TAL1* 5’ UTR into *Xho* *I* site of LHZ1138 |
| OZJY184R | GGTGTTTATTCTCAATTGGTAATAGACAATTTCTAATCGGGGTCTGAGCATTCAAAAG | The overlap extension PCR for inserting *TAL1* 5’ UTR into *Xho* *I* site of LHZ1138 |
| OZJY185F | AAATCGAAAAAAGAAGGATTATTCAAACAAGAAATGAGTAAAGGAGAAGAACTTTTCAC | The amplification of *KLMA*_*10828* 5’ UTR with homologous arms for insertion into *Xho* I site of LHZ1138 |
| OZJY185R | AATAATCCTTCTTTTTTCGATTTTCGAATATTTAATGTTGGGGTCTGAGCATTCAAAAG | The amplification of *KLMA*_*10828* 5’ UTR with homologous arms for insertion into *Xho* I site of LHZ1138 |
| OZJY186F | CAGAGTGGGATTAATCAAGAGACAGTGCAATTGTTAAAAAGATGAGTAAAGGAGAAGAACTTTTCAC | The amplification of *KLMA*_*50448* 5’ UTR with homologous arms for insertion into *Xho* I site of LHZ1138 |
| OZJY186R | TGTCTCTTGATTAATCCCACTCTGGTAGCCCACAATTCCTGGGGTCTGAGCATTCAAAAG | The amplification of *KLMA*_*50448* 5’ UTR with homologous arms for insertion into *Xho* I site of LHZ1138 |
| OZJY187F | CTTTTGAATGCTCAGACCCCAAGCTACACAAGTCGTTAGATTGGTTC | The amplification of *DYS1* 5’ UTR with homologous arms for insertion into *Xho* I site of LHZ1138 |
| OZJY187R | GTGAAAAGTTCTTCTCCTTTACTCATCGCTTAACAATTTCAATACCAATTAAACCAC | The amplification of *DYS1* 5’ UTR with homologous arms for insertion into *Xho* I site of LHZ1138 |
| OZJY188F | CTGGGAGTTACAGTAAAAGTGGAAAAAGCAAGCATGAGTAAAGGAGAAGAACTTTTCAC | The amplification of *RNA1* 5’ UTR with homologous arms for insertion into *Xho* I site of LHZ1138 |
| OZJY188R | GCTTTTTCCACTTTTACTGTAACTCCCAGTTCCTAGGGGTCTGAGCATTCAAAAG | The amplification of *RNA1* 5’ UTR with homologous arms for insertion into *Xho* I site of LHZ1138 |
| OZJY189F | CTTTTGAATGCTCAGACCCCAGGATTCGTTGCTGGAAAATCATCAG | The amplification of *MRPL33* 5’ UTR with homologous arms for insertion into *Xho* I site of LHZ1138 |
| OZJY189R | GTGAAAAGTTCTTCTCCTTTACTCATGATATTATATAATATGACGTGATACAATTAACG | The amplification of *MRPL33* 5’ UTR with homologous arms for insertion into *Xho* I site of LHZ1138 |
| OZJY190F | CTTTTGAATGCTCAGACCCCGGAGCTTTCTTTATATCGCCGACCG | The amplification of *SDH4* 5’ UTR with homologous arms for insertion into *Xho* I site of LHZ1138 |
| OZJY190R | GTGAAAAGTTCTTCTCCTTTACTCATTGTGCAATATGTAAATTCTATGGTTAAACGTTC | The amplification of *SDH4* 5’ UTR with homologous arms for insertion into *Xho* I site of LHZ1138 |
| OZJY191F | CATTTTTGGGTTATACAGTTGGTACAAGACCGTAATTGTGGATTGAAAGACAGGAAGATACACAATGAGTAAAGGAGAAGAACTTTTCAC | The amplification of *SEC61* 5’ UTR with homologous arms for insertion into *Xho* I site of LHZ1138 |
| OZJY191R | CTTGTACCAACTGTATAACCCAAAAATGCGCTTTTTGAACCTTCACAGACAGTGGAGAAATCCTACGGGGTCTGAGCATTCAAAAG | The amplification of *SEC61* 5’ UTR with homologous arms for insertion into *Xho* I site of LHZ1138 |
| OZJY192F | GTATAGGATTCCAAGCAAACTAGAAAAAACCAAAAAAAACATCAAGAACAATCAAGAAATGAGTAAAGGAGAAGAACTTTTCAC | The amplification of *GAL10* 5’ UTR with homologous arms for insertion into *Xho* I site of LHZ1138 |
| OZJY192R | CTAGTTTGCTTGGAATCCTATACTTTTAGGGTCTCCAGACTATTACTATTTTACCTTTTGGTTGGGGTCTGAGCATTCAAAAG | The amplification of *GAL10* 5’ UTR with homologous arms for insertion into *Xho* I site of LHZ1138 |
| OZJY193F | ATCCTAGAGCAAATAAAAACACCAACGGCCATGAGTAAAGGAGAAGAACTTTTCAC | The amplification of *GCD2* 5’ UTR with homologous arms for insertion into *Xho* I site of LHZ1138 |
| OZJY193R | GGCCGTTGGTGTTTTTATTTGCTCTAGGATGGGGTCTGAGCATTCAAAAG | The amplification of *GCD2* 5’ UTR with homologous arms for insertion into *Xho* I site of LHZ1138 |
| OZJY194F | CTTTTGAATGCTCAGACCCCAAGGAACAAGGACACTTTTTCATACAAAG | The amplification of *GAP1* 5’ UTR with homologous arms for insertion into *Xho* I site of LHZ1138 |
| OZJY194R | GTGAAAAGTTCTTCTCCTTTACTCATCTTTTAAAATTATCTGAGTTGAGTTGTGTGTG | The amplification of *GAP1* 5’ UTR with homologous arms for insertion into *Xho* I site of LHZ1138 |
| OZJY195F | CTTTTGAATGCTCAGACCCCAAGCTAAACAGCAAGCTAAACAGC | The overlap extension PCR for inserting *LAC12* 5’ UTR into *Xho* *I* site of LHZ1138 |
| OZJY195R | GTGAAAAGTTCTTCTCCTTTACTCATTATGAAAACTGATTATCGTCCTGTTTTATTTTTC | The overlap extension PCR for inserting *LAC12* 5’ UTR into *Xho* *I* site of LHZ1138 |
| OZJY196F | CTTGGAAATAGCATTATTATCACTCTATCCTTGAGCTATAGAAGAAGCCAAAAATACACGAGATGAGTAAAGGAGAAGAACTTTTCAC | The amplification of *SSP120* 5’ UTR with homologous arms for insertion into *Xho* I site of LHZ1138 |
| OZJY196R | TAATAATGCTATTTCCAAGTTTAAGTACAAAGCCTAATCGGGGTCTGAGCATTCAAAAG | The amplification of *SSP120* 5’ UTR with homologous arms for insertion into *Xho* I site of LHZ1138 |
| OZJY197F | CTTTTGAATGCTCAGACCCCAGGAGAAGTACTTTCAACTAGCATAG | The overlap extension PCR for inserting *UBI4* 5’ UTR into *Xho* *I* site of LHZ1138 |
| OZJY197R | GTGAAAAGTTCTTCTCCTTTACTCATTTTTAATGTCTTGATGAGCTTGAGTGTTG | The overlap extension PCR for inserting *UBI4* 5’ UTR into *Xho* *I* site of LHZ1138 |
| OZJY198F | AAGCAAAAATTAATATCTATAACTATACCATGAGTAAAGGAGAAGAACTTTTCAC | The amplification of *JEN1* 5’ UTR with homologous arms for insertion into *Xho* I site of LHZ1138 |
| OZJY198R | GGTATAGTTATAGATATTAATTTTTGCTTGGGGTCTGAGCATTCAAAAG | The amplification of *JEN1* 5’ UTR with homologous arms for insertion into *Xho* I site of LHZ1138 |
| OZJY199F | CTTTTGAATGCTCAGACCCCGGAGAAAGCACTATCCTAAAAAGCTG | The overlap extension PCR for inserting *VPS21* 5’ UTR into *Xho* *I* site of LHZ1138 |
| OZJY199R | GTGAAAAGTTCTTCTCCTTTACTCATCGTATAATTAAAACTTGGTTCTCTTTAATCC | The overlap extension PCR for inserting *VPS21* 5’ UTR into *Xho* *I* site of LHZ1138 |
| OZJY200F | CAAAGTAGTATTCGGTTTATTAGTATATCCGAATAACTAGCAACCAAAAAAGAAGATACAAATGAGTAAAGGAGAAGAACTTTTCAC | The amplification of *NHP2* 5’ UTR with homologous arms for insertion into *Xho* I site of LHZ1138 |
| OZJY200R | TAAACCGAATACTACTTTGATAGCTTTAATCCAAATGTGTGGGGTCTGAGCATTCAAAAG | The amplification of *NHP2* 5’ UTR with homologous arms for insertion into *Xho* I site of LHZ1138 |
| OZJY201F | GAGCCGATAATACATATTATCAAAAACAGCTTGATTGACCGGGAAGAAATATATACCATGAGTAAAGGAGAAGAACTTTTCAC | The amplification of *KLMA*_*40301* 5’ UTR with homologous arms for insertion into *Xho* I site of LHZ1138 |
| OZJY201R | GATAATATGTATTATCGGCTCTTTATAGCTAAGTAATTCTCCTTTTAGAAGAAAGTCCACGGGGTCTGAGCATTCAAAAG | The amplification of *KLMA*_*40301* 5’ UTR with homologous arms for insertion into *Xho* I site of LHZ1138 |
| OZJY202F | CAATTAAAGGGCCTTAGCAAGGGATAACGGACCGCAATCGAATCACCATGAGTAAAGGAGAAGAACTTTTCAC | The amplification of *VMA5* 5’ UTR with homologous arms for insertion into *Xho* I site of LHZ1138 |
| OZJY202R | GTTATCCCTTGCTAAGGCCCTTTAATTGCCTTTTTCGCACGGGGTCTGAGCATTCAAAAG | The amplification of *VMA5* 5’ UTR with homologous arms for insertion into *Xho* I site of LHZ1138 |
| OZJY203F | CTTTTGAATGCTCAGACCCCAAGGATCGTTGGATTTATATACATATATAAAGCAAG | The overlap extension PCR for inserting *SOR1* 5’ UTR into *Xho* *I* site of LHZ1138 |
| OZJY203R | GTGAAAAGTTCTTCTCCTTTACTCATGGTTGATAATTTGTATTTTTGTTATTGGTAGCG | The overlap extension PCR for inserting *SOR1* 5’ UTR into *Xho* *I* site of LHZ1138 |
| OZJY204F | CTTTTGAATGCTCAGACCCCGAAAACAAGAGTACATACAGTATTACTATACAGTAC | The overlap extension PCR for inserting *KYE1* 5’ UTR into *Xho* *I* site of LHZ1138 |
| OZJY204R | GTGAAAAGTTCTTCTCCTTTACTCATTATTGTTACTCTTGTTACTCTTGTTTTGTTCC | The overlap extension PCR for inserting *KYE1* 5’ UTR into *Xho* *I* site of LHZ1138 |
| OZJY205F | CTTTTGAATGCTCAGACCCCAAGTAACATCTTTTTGAGATATACATTACTATTATAAAAG | The overlap extension PCR for inserting *MDH2* 5’ UTR into *Xho* *I* site of LHZ1138 |
| OZJY205R | GTGAAAAGTTCTTCTCCTTTACTCATTTTTGAATCTGTTGCTGACACTTTTTC | The overlap extension PCR for inserting *MDH2* 5’ UTR into *Xho* *I* site of LHZ1138 |
| OZJY206F | CATTCGAATTGTTATACTCGGCTCCAAGAACAATAACAAGAAACAACAGTAAAAACCAAAAAAATGAGTAAAGGAGAAGAACTTTTCAC | The amplification of *FSF1* 5’ UTR with homologous arms for insertion into *Xho* I site of LHZ1138 |
| OZJY206R | GAGCCGAGTATAACAATTCGAATGGCCTAACTGCAATTGTCAACGTACTTTATACTTTTGTACTTTGTTGGGGTCTGAGCATTCAAAAG | The amplification of *FSF1* 5’ UTR with homologous arms for insertion into *Xho* I site of LHZ1138 |
| OZJY207F | CATAGAGTCCGAACTGTTGTGAGCTGTGGAGTGTTTCTGAATCTTAAAAAAGTCACCAACTAAAATGAGTAAAGGAGAAGAACTTTTCAC | The amplification of *KLMA*_*50610* 5’ UTR with homologous arms for insertion into *Xho* I site of LHZ1138 |
| OZJY207R | CAACAGTTCGGACTCTATGAACGCTAGTTAAAACGTTGCTGGGGTCTGAGCATTCAAAAG | The amplification of *KLMA*_*50610* 5’ UTR with homologous arms for insertion into *Xho* I site of LHZ1138 |
| OZJY208F | GTACGTTATATTTTAGATTTAGAGGTATAGTTGTGGTTAAAAACTAACAGACATTGCAAAGATGAGTAAAGGAGAAGAACTTTTCAC | The amplification of *RSM23* 5’ UTR with homologous arms for insertion into *Xho* I site of LHZ1138 |
| OZJY208R | CCTCTAAATCTAAAATATAACGTACAATAAGTACAAATGTGGTTAATATTCAAATTGAACGGGGTCTGAGCATTCAAAAG | The amplification of *RSM23* 5’ UTR with homologous arms for insertion into *Xho* I site of LHZ1138 |
| OZJY209F | GTACAAGGTAGCTGCTGTAAAAAAAGGCAAACACACAGTTATATCTATACATTTTTGAAAGATGAGTAAAGGAGAAGAACTTTTCAC | The amplification of *RSM27* 5’ UTR with homologous arms for insertion into *Xho* I site of LHZ1138 |
| OZJY209R | TACAGCAGCTACCTTGTACTAGCACCTGTGAATCGGGGTCTGAGCATTCAAAAG | The amplification of *RSM27* 5’ UTR with homologous arms for insertion into *Xho* I site of LHZ1138 |
| OZJY210F | GGACATCTGCCAAGCAATAAAAAAATAAAAAGTATGAGTAAAGGAGAAGAACTTTTCAC | The amplification of *KLMA*_*30482* 5’ UTR with homologous arms for insertion into *Xho* I site of LHZ1138 |
| OZJY210R | TATTTTTTTATTGCTTGGCAGATGTCCTTCTTCTTCTGGGGTCTGAGCATTCAAAAG | The amplification of *KLMA*_*30482* 5’ UTR with homologous arms for insertion into *Xho* I site of LHZ1138 |
| OZJY211F | CAAATATCAACGTCTAGCAAGCTCATCGCATCACATATTTAATTTAGATCCTAAAAAAATGAGTAAAGGAGAAGAACTTTTCAC | The amplification of *FBP1* 5’ UTR with homologous arms for insertion into *Xho* I site of LHZ1138 |
| OZJY211R | GCTTGCTAGACGTTGATATTTGCTGTATCGACGATGCTTGGGGTCTGAGCATTCAAAAG | The amplification of *FBP1* 5’ UTR with homologous arms for insertion into *Xho* I site of LHZ1138 |
| OZJY212F | CTTTTGAATGCTCAGACCCCGGAAAAACATCTTCAGGTTAAGGTTG | The overlap extension PCR for inserting *GLC8* 5’ UTR into *Xho* *I* site of LHZ1138 |
| OZJY212R | GTGAAAAGTTCTTCTCCTTTACTCATGATTGTAATGATAAAAGCCTTGATTGATAATAC | The overlap extension PCR for inserting *GLC8* 5’ UTR into *Xho* *I* site of LHZ1138 |
| OZJY213F | CTTTTGAATGCTCAGACCCCACGGGGAATCAAGAAGACTTC | The overlap extension PCR for inserting *QCR2* 5’ UTR into *Xho* *I* site of LHZ1138 |
| OZJY213R | TGAAAAGTTCTTCTCCTTTACTCATTTCGTCTTATTATTCTTTCTTGTTATTATTCTTTC | The overlap extension PCR for inserting *QCR2* 5’ UTR into *Xho* *I* site of LHZ1138 |
| OZJY214F | CAAGGTTCGGTATTACATACACAAACACACACATAAATTTCATAAAAAAATGAGTAAAGGAGAAGAACTTTTCAC | The amplification of *YAR1* 5’ UTR with homologous arms for insertion into *Xho* I site of LHZ1138 |
| OZJY214R | GTGTATGTAATACCGAACCTTGGTGTCTGATAAAGCTGCGGGGTCTGAGCATTCAAAAG | The amplification of *YAR1* 5’ UTR with homologous arms for insertion into *Xho* I site of LHZ1138 |
| OZJY215F | GAAACCAGGATATATAGTCACCAAATTCCTCGAAAAACCATGAGTAAAGGAGAAGAACTTTTCAC | The amplification of *CLU1* 5’ UTR with homologous arms for insertion into *Xho* I site of LHZ1138 |
| OZJY215R | TGGTGACTATATATCCTGGTTTCTTATGTTTTAAACTATTGGGGTCTGAGCATTCAAAAG | The amplification of *CLU1* 5’ UTR with homologous arms for insertion into *Xho* I site of LHZ1138 |
| OZJY216F | TAGGGGAACGCTAAAAATAAGGCTACATTCACAATGAGTAAAGGAGAAGAACTTTTCAC | The amplification of *RRP45* 5’ UTR with homologous arms for insertion into *Xho* I site of LHZ1138 |
| OZJY216R | GTAGCCTTATTTTTAGCGTTCCCCTAGCCTCAAGCTCCTGGGGTCTGAGCATTCAAAAG | The amplification of *RRP45* 5’ UTR with homologous arms for insertion into *Xho* I site of LHZ1138 |
| OZJY217F | ATATCATAAATATTATTATTCTGGAAGGTAACCAACACAGAAATCTAAAAAGAAGTATCAAGAATGAGTAAAGGAGAAGAACTTTTCAC | The amplification of *SSZ1* 5’ UTR with homologous arms for insertion into *Xho* I site of LHZ1138 |
| OZJY217R | GAATAATAATATTTATGATATCTAATCAAAAGGAGAACCAATTGGGGTCTGAGCATTCAAAAG | The amplification of *SSZ1* 5’ UTR with homologous arms for insertion into *Xho* I site of LHZ1138 |
| OZJY218F | GTGACAAGTGGCCGATAATATTCATAGAGATTCCATAAAAAATGAGTAAAGGAGAAGAACTTTTCAC | The amplification of *CBP6* 5’ UTR with homologous arms for insertion into *Xho* I site of LHZ1138 |
| OZJY218R | TGAATATTATCGGCCACTTGTCACAACGTCACACTGGCTTGGGGTCTGAGCATTCAAAAG | The amplification of *CBP6* 5’ UTR with homologous arms for insertion into *Xho* I site of LHZ1138 |
| OZJY219F | CGCCAAGACTCTTTATTTTAAGTGAGTGAATTTTTACAGAGCTAAAAAAGAAGAAGAAGAATTATGAGTAAAGGAGAAGAACTTTTCAC | The amplification of *SSA3* 5’ UTR with homologous arms for insertion into *Xho* I site of LHZ1138 |
| OZJY219R | CACTCACTTAAAATAAAGAGTCTTGGCGACAGTTTACTTTGTTTAACTTAATAGTTTTCGTTAGTGGCTTGGGGTCTGAGCATTCAAAAG | The amplification of *SSA3* 5’ UTR with homologous arms for insertion into *Xho* I site of LHZ1138 |
| OZJY220F | GAAGGAAGACGTACAAGACAAGACAAGACCGGACAAGAAAAAGAATTGTAAACATACAAAATGAGTAAAGGAGAAGAACTTTTCAC | The amplification of *TVP18* 5’ UTR with homologous arms for insertion into *Xho* I site of LHZ1138 |
| OZJY220R | GTCTTGTCTTGTACGTCTTCCTTCTGGACTAGTACCTGCTCCCTTCAAGCGAATCCTTGGGGTCTGAGCATTCAAAAG | The amplification of *TVP18* 5’ UTR with homologous arms for insertion into *Xho* I site of LHZ1138 |
| OZJY221F | CCAGCATCTTAAAAAGAATAGAACAAGGCAGATAAGGTTTGTACGAATATACAAATAAGTGAAGATGAGTAAAGGAGAAGAACTTTTCAC | The amplification of *DBP10* 5’ UTR with homologous arms for insertion into *Xho* I site of LHZ1138 |
| OZJY221R | CTGCCTTGTTCTATTCTTTTTAAGATGCTGGAAAAAAGACAGCACAGTGGAGTATAGTAAACGAATACAGGGGTCTGAGCATTCAAAAG | The amplification of *DBP10* 5’ UTR with homologous arms for insertion into *Xho* I site of LHZ1138 |
| OZJY222F | ATTGATCTCTTGGTTGCTGATCAAAAGTTTTCAAAGTACAACTAATTATACATTCAAAAAGAGATGAGTAAAGGAGAAGAACTTTTCAC | The amplification of *EHT1* 5’ UTR with homologous arms for insertion into *Xho* I site of LHZ1138 |
| OZJY222R | CAGCAACCAAGAGATCAATGAACAATGTCGAAAATGGTTGGGGTCTGAGCATTCAAAAG | The amplification of *EHT1* 5’ UTR with homologous arms for insertion into *Xho* I site of LHZ1138 |
| OZJY223F | GCTCTTGTTACGTTGTGGAACAAAAGGTGCAGTAAAGTTTAACAGTTTCAACATGAGTAAAGGAGAAGAACTTTTCAC | The amplification of *PKP1* 5’ UTR with homologous arms for insertion into *Xho* I site of LHZ1138 |
| OZJY223R | GTTCCACAACGTAACAAGAGCTTTTTCTGTCTCTGGTTGGGGTCTGAGCATTCAAAAG | The amplification of *PKP1* 5’ UTR with homologous arms for insertion into *Xho* I site of LHZ1138 |
| OZJY224F | CAAACTAAATCACATATACATACACAATGAGTAAAGGAGAAGAACTTTTCAC | The overlap extension PCR for inserting *HTB1* 5’ UTR into *Xho* *I* site of LHZ1138 |
| OZJY224R | TGTGTATGTATATGTGATTTAGTTTGGTTTGGTTGGGGTCTGAGCATTCAAAAG | The overlap extension PCR for inserting *HTB1* 5’ UTR into *Xho* *I* site of LHZ1138 |
| OZJY225F | TACGCCCCCCAGATAACCAAATAATACAGAAAAAATGAGTAAAGGAGAAGAACTTTTCAC | The amplification of *HTB1* 5’ UTR with homologous arms for insertion into *Xho* I site of LHZ1138 |
| OZJY225R | TATTTGGTTATCTGGGGGGCGTAGTAGTTACTTACTGACTGGGGTCTGAGCATTCAAAAG | The amplification of *HTB1* 5’ UTR with homologous arms for insertion into *Xho* I site of LHZ1138 |
| OZJY226F | TAGTGTTATAATTATTACTATTATCATCATCATCAGTACAGCTATAATAGAAAAAATCAAGAGATGAGTAAAGGAGAAGAACTTTTCAC | The amplification of *NCE102* 5’ UTR with homologous arms for insertion into *Xho* I site of LHZ1138 |
| OZJY226R | ATAGTAATAATTATAACACTAGTATAAGCTCTTGTTCTCTGGGGTCTGAGCATTCAAAAG | The amplification of *NCE102* 5’ UTR with homologous arms for insertion into *Xho* I site of LHZ1138 |
| OZJY227F | GTTGAATTAGCTATACACTTGATATTAACCCCAGACAAAAAAATGAGTAAAGGAGAAGAACTTTTCAC | The amplification of *KLMA*_*60481* 5’ UTR with homologous arms for insertion into *Xho* I site of LHZ1138 |
| OZJY227R | AGTGTATAGCTAATTCAACAGTCTTTTAGCTGATTATACTGGGGTCTGAGCATTCAAAAG | The amplification of *KLMA*_*60481* 5’ UTR with homologous arms for insertion into *Xho* I site of LHZ1138 |
| OZJY228F | GCATCTTCGAGAAGGTAACGCTGGTAGTAGATTAGAAACATCATCGTTCCAAAAACTAAAAATGAGTAAAGGAGAAGAACTTTTCAC | The amplification of *NSR1* 5’ UTR with homologous arms for insertion into *Xho* I site of LHZ1138 |
| OZJY228R | CCAGCGTTACCTTCTCGAAGATGCTTTAGCAATTAATTGTTCTAAGCAACAATTCAAATGCTCTGGGGTCTGAGCATTCAAAAG | The amplification of *NSR1* 5’ UTR with homologous arms for insertion into *Xho* I site of LHZ1138 |
| OZJY229F | GCTATAGTGTGATATATATATACTGAATAACATTATTTGAACTGAGTTTGCATATACCTACAAAATGAGTAAAGGAGAAGAACTTTTCAC | The amplification of *SLD5* 5’ UTR with homologous arms for insertion into *Xho* I site of LHZ1138 |
| OZJY229R | CAGTATATATATATCACACTATAGCAGAAAGCTAGCCTTTTTAACCATGTACCTAGGGGTCTGAGCATTCAAAAG | The amplification of *SLD5* 5’ UTR with homologous arms for insertion into *Xho* I site of LHZ1138 |
| OZJY230F | CAACAAACAAGTTTTTGCTTTCCTAATTTTCTAACAAAAATGAGTAAAGGAGAAGAACTTTTCAC | The amplification of *HCH1* 5’ UTR with homologous arms for insertion into *Xho* I site of LHZ1138 |
| OZJY230R | GGAAAGCAAAAACTTGTTTGTTGTATACGCAAAATGCAGTGGGGTCTGAGCATTCAAAAG | The amplification of *HCH1* 5’ UTR with homologous arms for insertion into *Xho* I site of LHZ1138 |
| OZJY231F | TAAGTAAGTGGTTACTACATTTAAAAACAAAATCATACACAACACAATGAGTAAAGGAGAAGAACTTTTCAC | The amplification of *ADH1* 5’ UTR with homologous arms for insertion into *Xho* I site of LHZ1138 |
| OZJY231R | ATGTAGTAACCACTTACTTAATTTAAGTTGTGCTTGGGGTCTGAGCATTCAAAAG | The amplification of *ADH1* 5’ UTR with homologous arms for insertion into *Xho* I site of LHZ1138 |
| OZJY232F | CACACCCCCATACACATATATAAAAACACAATTGCGTCACAATGAGTAAAGGAGAAGAACTTTTCAC | The amplification of *POR1* 5’ UTR with homologous arms for insertion into *Xho* I site of LHZ1138 |
| OZJY232R | TTTTATATATGTGTATGGGGGTGTGATTTAAATTAACCTTGGGGTCTGAGCATTCAAAAG | The amplification of *POR1* 5’ UTR with homologous arms for insertion into *Xho* I site of LHZ1138 |
| OZJY233F | CGCCCTACTAATACACTTAAAAGAACTTCAATAATGAGTAAAGGAGAAGAACTTTTCAC | The overlap extension PCR for inserting *INO1* 5’ UTR into *Xho* *I* site of LHZ1138 |
| OZJY233R | CTTTTAAGTGTATTAGTAGGGCGTATTGTGTATTAAGTCAGATTTGGCTTGGGGTCTGAGCATTCAAAAG | The overlap extension PCR for inserting *INO1* 5’ UTR into *Xho* *I* site of LHZ1138 |
| OZJY234F | ACGCTAGCTACAAACTAACTCCAAAAACAACAACCAACAACTAGAAATGAGTAAAGGAGAAGAACTTTTCAC | The amplification of *ADH2* 5’ UTR with homologous arms for insertion into *Xho* I site of LHZ1138 |
| OZJY234R | TGGAGTTAGTTTGTAGCTAGCGTTTTAATGCTTGATTGCTGGGGTCTGAGCATTCAAAAG | The amplification of *ADH2* 5’ UTR with homologous arms for insertion into *Xho* I site of LHZ1138 |
| OZJY235F | TACTATAACAAACACTAATCAATCAAACAAGCATTATTGAACGCAAAATATTAAAAAATGAGTAAAGGAGAAGAACTTTTCAC | The amplification of *HGT1* 5’ UTR with homologous arms for insertion into *Xho* I site of LHZ1138 |
| OZJY235R | TGATTAGTGTTTGTTATAGTATTAAAGCTTGCTATTGCTTGGGGTCTGAGCATTCAAAAG | The amplification of *HGT1* 5’ UTR with homologous arms for insertion into *Xho* I site of LHZ1138 |
| OZJY236F | CAATTTTTATTGAGTCTTGGACCGTCGATTGCTAGAACAAAAAAATCAAATACACAGTTAAAAATGAGTAAAGGAGAAGAACTTTTCAC | The amplification of *TIM9* 5’ UTR with homologous arms for insertion into *Xho* I site of LHZ1138 |
| OZJY236R | CCAAGACTCAATAAAAATTGACTCTTGTATCTTTCTTCACGGGGTCTGAGCATTCAAAAG | The amplification of *TIM9* 5’ UTR with homologous arms for insertion into *Xho* I site of LHZ1138 |
| OZJY237F | CTGGAGATAGTAGCAGCTGCATATAATAGTTAATCACAAGCCAACCTAAAAACAATCATCATCATGAGTAAAGGAGAAGAACTTTTCAC | The amplification of *SAM2* 5’ UTR with homologous arms for insertion into *Xho* I site of LHZ1138 |
| OZJY237R | CAGCTGCTACTATCTCCAGCGTTGCTAGTGGCTCTAACCTGGGGTCTGAGCATTCAAAAG | The amplification of *SAM2* 5’ UTR with homologous arms for insertion into *Xho* I site of LHZ1138 |
| OZJY238F | AGTAACTACAATAAACTAATAATAAAATATCAGGATGAGTAAAGGAGAAGAACTTTTCAC | The amplification of *MRP49* 5’ UTR with homologous arms for insertion into *Xho* I site of LHZ1138 |
| OZJY238R | ATTATTAGTTTATTGTAGTTACTTCAATCTGTAACGTGTTGGGGTCTGAGCATTCAAAAG | The amplification of *MRP49* 5’ UTR with homologous arms for insertion into *Xho* I site of LHZ1138 |
| OZJY239F | GCATAATTATTAAACACAGAAGATAAGAAACAATGAGTAAAGGAGAAGAACTTTTCAC | The overlap extension PCR for inserting *KLMA*_*60492* 5’ UTR into *Xho* *I* site of LHZ1138 |
| OZJY239R | CTTCTGTGTTTAATAATTATGCTTTAAAGCTTATAATAGTATAGTGTTCTATATTATTACCTGCGGGGTCTGAGCATTCAAAAG | The overlap extension PCR for inserting *KLMA*_*60492* 5’ UTR into *Xho* *I* site of LHZ1138 |
| OZJY240F | CTTTTGAATGCTCAGACCCCAGAATTCAGAGTAGAGAGAAAGACAATAAG | The overlap extension PCR for inserting *NCB2* 5’ UTR into *Xho* *I* site of LHZ1138 |
| OZJY240R | GTGAAAAGTTCTTCTCCTTTACTCATCTTTGTTTCTTTCTTCGAGTCTCTCTC | The overlap extension PCR for inserting *NCB2* 5’ UTR into *Xho* *I* site of LHZ1138 |
| OZJY241F | ATTGCTAGAACAATCAAATACACAGTTAAAAATGAGTAAAGGAG | The generation of *TIM9* len2^<1>^ mutant 5’ UTR by PCR-based mutagenesis |
| OZJY241R | GTGTATTTGATTGTTCTAGCAATCGACGGTCCAAGAC | The generation of *TIM9* len2 mutant 5’ UTR by PCR-based mutagenesis |
| OZJY242F | ATTGCTAGAACAAAAATCAAATACACAGTTAAAAATGAGTAAAGGAG | The generation of *TIM9* len5 mutant 5’ UTR by PCR-based mutagenesis |
| OZJY242R | GTGTATTTGATTTTTGTTCTAGCAATCGACGGTCCAAGAC | The generation of *TIM9* len5 mutant 5’ UTR by PCR-based mutagenesis |
| OZJY243F | ATTGCTAGAACAAAAAATCAAATACACAGTTAAAAATGAGTAAAGGAG | The generation of *TIM9* len6 mutant 5’ UTR by PCR-based mutagenesis |
| OZJY243R | GTGTATTTGATTTTTTGTTCTAGCAATCGACGGTCCAAGAC | The generation of *TIM9* len6 mutant 5’ UTR by PCR-based mutagenesis |
| OZJY244F | ATTGCTAGAACAAAAAAAAAAATCAAATACACAGTTAAAAATGAGTAAAGGAG | The generation of *TIM9* len11 mutant 5’ UTR by PCR-based mutagenesis |
| OZJY244R | GTGTATTTGATTTTTTTTTTTGTTCTAGCAATCGACGGTCCAAGAC | The generation of *TIM9* len11 mutant 5’ UTR by PCR-based mutagenesis |
| OZJY245F | GAAGAAAAAAAAAAGATACAAGAGTCAATTTTTATTGAGTCTTGGACCGTCG | The generation of *TIM9* u67^<2>^ mutant 5’ UTR by PCR-based mutagenesis |
| OZJY245R | GACTCTTGTATCTTTTTTTTTTCTTCACGGGGTCTGAGC | The generation of *TIM9* u67 mutant 5’ UTR by PCR-based mutagenesis |
| OZJY246F | GATACAAAAAAAAAGAGTCAATTTTTATTGAGTCTTGGACCGTCG | The generation of *TIM9* u60 mutant 5’ UTR by PCR-based mutagenesis |
| OZJY246R | CTCAATAAAAATTGACTCTTTTTTTTTGTATCTTTCTTCACGGGGTCTG | The generation of *TIM9* u60 mutant 5’ UTR by PCR-based mutagenesis |
| OZJY247F | TGGACCGTCGATTGCTAGAACTCAAATACACAGTTAAAAATGAGTAAAGGAG | The generation of *TIM9* u39 mutant 5’ UTR by PCR-based mutagenesis |
| OZJY247R | TTCTAGCAATCGACGGTCCATTTTTTTAGACTCAATAAAAATTGACTCTTGTATCTTTC | The generation of *TIM9* u39 mutant 5’ UTR by PCR-based mutagenesis |
| OZJY248F | CAAATACACAGTTAAAAAAAAAAAATGAGTAAAGGAGAAGAACTTTTCAC | The generation of *TIM9* d4^<3>^ mutant 5’ UTR by PCR-based mutagenesis |
| OZJY248R | CATTTTTTTTTTTTAACTGTGTATTTGAGTTCTAGCAATCGACGGTCCAAG | The generation of *TIM9* d4 mutant 5’ UTR by PCR-based mutagenesis |
| OZJY249F | GGTAACCAACACAGAAATCTAGAAGTATCAAGAATGAGTAAAGGAGAAGAAC | The generation of *SSZ1* len1 mutant 5’ UTR by PCR-based mutagenesis |
| OZJY249R | ACTCATTCTTGATACTTCTAGATTTCTGTGTTGGTTACCTTCCAG | The generation of *SSZ1* len1 mutant 5’ UTR by PCR-based mutagenesis |
| OZJY250F | GGTAACCAACACAGAAATCTAAGAAGTATCAAGAATGAGTAAAGGAGAAGAAC | The generation of *SSZ1* len2 mutant 5’ UTR by PCR-based mutagenesis |
| OZJY250R | ACTCATTCTTGATACTTCTTAGATTTCTGTGTTGGTTACCTTCCAG | The generation of *SSZ1* len2 mutant 5’ UTR by PCR-based mutagenesis |
| OZJY251F | GGTAACCAACACAGAAATCTAAAAAAAAGAAGTATCAAGAATGAGTAAAGGAGAAGAAC | The generation of *SSZ1* len8 mutant 5’ UTR by PCR-based mutagenesis |
| OZJY251R | ACTCATTCTTGATACTTCTTTTTTTTAGATTTCTGTGTTGGTTACCTTCCAG | The generation of *SSZ1* len8 mutant 5’ UTR by PCR-based mutagenesis |
| OZJY252F | TAACCAACACAGAAATCTAAAAAAAAAAGAAGTATCAAGAATGAGTAAAGGAGAAGAAC | The generation of *SSZ1* len10 mutant 5’ UTR by PCR-based mutagenesis |
| OZJY252R | ACTCATTCTTGATACTTCTTTTTTTTTTAGATTTCTGTGTTGGTTACCTTCCAG | The generation of *SSZ1* len10 mutant 5’ UTR by PCR-based mutagenesis |
| OZJY253F | CCTTTTGATTAGATATCATAAATATTATTATTCTGGAAGGTAACCAACACAG | The generation of *SSZ1* u75 mutant 5’ UTR by PCR-based mutagenesis |
| OZJY253R | TATTTATGATATCTAATCAAAAGGAGAACTTTTTCAATTGGGGTCTGAGCATTC | The generation of *SSZ1* u75 mutant 5’ UTR by PCR-based mutagenesis |
| OZJY254F | GATAAAAATAGATATCATAAATATTATTATTCTGGAAGGTAACCAACACAG | The generation of *SSZ1* u61 mutant 5’ UTR by PCR-based mutagenesis |
| OZJY254R | GAATAATAATATTTATGATATCTATTTTTATCAAAAGGAGAACCAATTGGGGTC | The generation of *SSZ1* u61 mutant 5’ UTR by PCR-based mutagenesis |
| OZJY255F | TTCTGGAAGGTAACCAACACAGAAATCTGAAGTATCAAGAATGAGTAAAGGAGAAGAAC | The generation of *SSZ1* u40 mutant 5’ UTR by PCR-based mutagenesis |
| OZJY255R | GTTGGTTACCTTCCAGAATTTTTTAATAATATTTATGATATCTAATCAAAAGGAGAACC | The generation of *SSZ1* u40 mutant 5’ UTR by PCR-based mutagenesis |
| OZJY256F | ACAGAAATCTGAAGTATAAAAACAAGAATGAGTAAAGGAGAAGAACTTTTC | The generation of *SSZ1* d5 mutant 5’ UTR by PCR-based mutagenesis |
| OZJY256R | TTACTCATTCTTGTTTTTATACTTCAGATTTCTGTGTTGGTTACCTTCC | The generation of *SSZ1* d5 mutant 5’ UTR by PCR-based mutagenesis |
| OZJY257F | ATTAACGAGTGATAGAAGACCAAACACAGGCACAGAGCC | The generation of *FIM1*_*2796* len0 mutant 5’ UTR by PCR-based mutagenesis |
| OZJY257R | GCTCTGTGCCTGTGTTTGGTCTTCTATCACTCGTTAATTAGCTACCTC | The generation of *FIM1*_*2796* len0 mutant 5’ UTR by PCR-based mutagenesis |
| OZJY258F | ATTAACGAGTGATAGAAGACACAAACACAGGCACAGAGCC | The generation of *FIM1*_*2796* len1 mutant 5’ UTR by PCR-based mutagenesis |
| OZJY258R | GCTCTGTGCCTGTGTTTGTGTCTTCTATCACTCGTTAATTAGCTACCTC | The generation of *FIM1*_*2796* len1 mutant 5’ UTR by PCR-based mutagenesis |
| OZJY259F | ATTAACGAGTGATAGAAGACAAAAAAAAACAAACACAGGCACAGAGCC | The generation of *FIM1*_*2796* len9 mutant 5’ UTR by PCR-based mutagenesis |
| OZJY259R | GCTCTGTGCCTGTGTTTGTTTTTTTTTGTCTTCTATCACTCGTTAATTAGCTACCTC | The generation of *FIM1*_*2796* len9 mutant 5’ UTR by PCR-based mutagenesis |
| OZJY260F | ATTAACGAGTGATAGAAGACAAAAAAAAAACAAACACAGGCACAGAGCC | The generation of *FIM1*_*2796* len10 mutant 5’ UTR by PCR-based mutagenesis |
| OZJY260R | GCTCTGTGCCTGTGTTTGTTTTTTTTTTGTCTTCTATCACTCGTTAATTAGCTACCTC | The generation of *FIM1*_*2796* len10 mutant 5’ UTR by PCR-based mutagenesis |
| OZJY261F | CAAAAAAAGACGGACAAAAGAGTATTAGCAGCGAGGTAGCTAATTAACGAGTGATAG | The generation of *FIM1*_*2796* u114 mutant 5’ UTR by PCR-based mutagenesis |
| OZJY261R | CTAATACTCTTTTGTCCGTCTTTTTTTGTCTCGTCTCGGGGTCTGAG | The generation of *FIM1*_*2796* u114 mutant 5’ UTR by PCR-based mutagenesis |
| OZJY262F | GACGGACAAAAAAAAAAGAGTATTAGCAGCGAGGTAGCTAATTAACGAGTGATAG | The generation of *FIM1*_*2796* u103 mutant 5’ UTR by PCR-based mutagenesis |
| OZJY262R | CTCGCTGCTAATACTCTTTTTTTTTTGTCCGTCTGTCTCGTCTCG | The generation of *FIM1*_*2796* u103 mutant 5’ UTR by PCR-based mutagenesis |
| OZJY263F | CAAAAAATAATTAACGAGTGATAGAAGACCAAACACAGGCACAGAGCCAC | The generation of *FIM1*_*2796* u81 mutant 5’ UTR by PCR-based mutagenesis |
| OZJY263R | GTTTGGTCTTCTATCACTCGTTAATTATTTTTTGCTACCTCGCTGCTAATACTCTTTTG | The generation of *FIM1*_*2796* u81 mutant 5’ UTR by PCR-based mutagenesis |
| OZJY264F | AGACCAAACACAGGCAAAAAAACAGAGCCACAGGAAAGAGG | The generation of *FIM1*_*2796* d48 mutant 5’ UTR by PCR-based mutagenesis |
| OZJY264R | CTGTTTTTTTGCCTGTGTTTGGTCTTCTATCACTCGTTAATTAGCTACCTC | The generation of *FIM1*_*2796* d48 mutant 5’ UTR by PCR-based mutagenesis |
| OZJY265F | CGATTCAGCAACCCACAAACAAACACAGCTTCCAGAAAG | The generation of *QRI1* len0 mutant 5’ UTR by PCR-based mutagenesis |
| OZJY265R | GCTGTGTTTGTTTGTGGGTTGCTGAATCGCCTCCCCTAATAC | The generation of *QRI1* len0 mutant 5’ UTR by PCR-based mutagenesis |
| OZJY266F | CGATTCAGCAACCAAACACAAACAAACACAGCTTCCAGAAAG | The generation of *QRI1* len3 mutant 5’ UTR by PCR-based mutagenesis |
| OZJY266R | GCTGTGTTTGTTTGTGTTTGGTTGCTGAATCGCCTCCCCTAATAC | The generation of *QRI1* len3 mutant 5’ UTR by PCR-based mutagenesis |
| OZJY267F | CGATTCAGCAACCAAAAAAAAACACAAACAAACACAGCTTCCAGAAAG | The generation of *QRI1* len9 mutant 5’ UTR by PCR-based mutagenesis |
| OZJY267R | GCTGTGTTTGTTTGTGTTTTTTTTTGGTTGCTGAATCGCCTCCCCTAATAC | The generation of *QRI1* len9 mutant 5’ UTR by PCR-based mutagenesis |
| OZJY268F | CGATTCAGCAACCAAAAAAAAAACACAAACAAACACAGCTTCCAGAAAG | The generation of *QRI1* len10 mutant 5’ UTR by PCR-based mutagenesis |
| OZJY268R | GCTGTGTTTGTTTGTGTTTTTTTTTTGGTTGCTGAATCGCCTCCCCTAATAC | The generation of *QRI1* len10 mutant 5’ UTR by PCR-based mutagenesis |
| OZJY269F | CTTAAAAAAATAATAACTTACTGGTGTGGTATTAGGGGAGGCGATTCAGC | The generation of *QRI1* u94 mutant 5’ UTR by PCR-based mutagenesis |
| OZJY269R | CCACACCAGTAAGTTATTATTTTTTTAAGATTCGGGGTCTGAGCATTC | The generation of *QRI1* u94 mutant 5’ UTR by PCR-based mutagenesis |
| OZJY270F | GTATTAGGGGAGGCGATTCAGCAACCCACAAACAAACACAGCTTCCAGAAAG | The generation of *QRI1* u76 mutant 5’ UTR by PCR-based mutagenesis |
| OZJY270R | CTGAATCGCCTCCCCTAATACCTTTTTTACACCAGTAAGTTATTATAAGATTCGGGGTC | The generation of *QRI1* u76 mutant 5’ UTR by PCR-based mutagenesis |
| OZJY271F | CAACCCACAAACAAAAAAAAACACAGCTTCCAGAAAGCAAGC | The generation of *QRI1* d40 mutant 5’ UTR by PCR-based mutagenesis |
| OZJY271R | CTGTGTTTTTTTTTGTTTGTGGGTTGCTGAATCGCCTCCCCTAATAC | The generation of *QRI1* d40 mutant 5’ UTR by PCR-based mutagenesis |
| OZJY272F | CAAACAAACACAGCTTCCAGAAAGCAAAAAAAAGCCCTTCCACCCTAGACAAG | The generation of *QRI1* d22 mutant 5’ UTR by PCR-based mutagenesis |
| OZJY272R | GCTTTCTGGAAGCTGTGTTTGTTTGTGGGTTGCTGAATCGCCTCCCCTAATAC | The generation of *QRI1* d22 mutant 5’ UTR by PCR-based mutagenesis |
| OZJY273F | CTATAGAAGAAGCCTACACGAGATGAGTAAAGGAGAAGAACTTTTC | The generation of *SSP120* len0 mutant 5’ UTR by PCR-based mutagenesis |
| OZJY273R | CTCATCTCGTGTAGGCTTCTTCTATAGCTCAAGGATAGAGTG | The generation of *SSP120* len0 mutant 5’ UTR by PCR-based mutagenesis |
| OZJY274F | CTATAGAAGAAGCCAAATACACGAGATGAGTAAAGGAGAAGAACTTTTC | The generation of *SSP120* len3 mutant 5’ UTR by PCR-based mutagenesis |
| OZJY274R | CTCATCTCGTGTATTTGGCTTCTTCTATAGCTCAAGGATAGAGTG | The generation of *SSP120* len3 mutant 5’ UTR by PCR-based mutagenesis |
| OZJY275F | TATCACTCTATCCTTGAGCTATAGAAGAAGCCTACACGAGATGAGTAAAGGAGAAGAAC | The generation of *SSP120* u43 mutant 5’ UTR by PCR-based mutagenesis |
| OZJY275R | ATAGCTCAAGGATAGAGTGATAATATTTTTATGCTATTTCCAAGTTTAAGTACAAAGCC | The generation of *SSP120* u43 mutant 5’ UTR by PCR-based mutagenesis |
| OZJY276F | GAGAAAAACTATAGAAGAAGCCTACACGAGATGAGTAAAGGAGAAGAACTTTTC | The generation of *SSP120* u22 mutant 5’ UTR by PCR-based mutagenesis |
| OZJY276R | GTAGGCTTCTTCTATAGTTTTTCTCAAGGATAGAGTGATAATAATGCTATTTCCAAG | The generation of *SSP120* u22 mutant 5’ UTR by PCR-based mutagenesis |
| OZJY277F | GAGTTACAGTAAAAGTGGAAAGCAAGCATGAGTAAAGGAGAAGAAC | The generation of *RNA1* len3 mutant 5’ UTR by PCR-based mutagenesis |
| OZJY277R | CCTTTACTCATGCTTGCTTTCCACTTTTACTGTAACTCCCAGTTCCTAG | The generation of *RNA1* len3 mutant 5’ UTR by PCR-based mutagenesis |
| OZJY278F | GAGTTACAGTAAAAGTGGAAAAAAAAAAAGCAAGCATGAGTAAAGGAGAAGAAC | The generation of *RNA1* len11 mutant 5’ UTR by PCR-based mutagenesis |
| OZJY278R | CCTTTACTCATGCTTGCTTTTTTTTTTTCCACTTTTACTGTAACTCCCAGTTCCTAG | The generation of *RNA1* len11 mutant 5’ UTR by PCR-based mutagenesis |
| OZJY279F | CAAAAATAGGAACTGGGAGTTACAGTAAAAGTGGGCAAGCATGAGTAAAGGAGAAGAAC | The generation of *RNA1* u34 mutant 5’ UTR by PCR-based mutagenesis |
| OZJY279R | ACTCCCAGTTCCTATTTTTGGGGTCTGAGCATTCAAAAGATAATAATAATAAATATAAC | The generation of *RNA1* u34 mutant 5’ UTR by PCR-based mutagenesis |
| OZJY280F | GTTAAAAAACAGTAAAAGTGGGCAAGCATGAGTAAAGGAGAAGAAC | The generation of *RNA1* u18 mutant 5’ UTR by PCR-based mutagenesis |
| OZJY280R | CTTGCCCACTTTTACTGTTTTTTAACTCCCAGTTCCTAGGGGTC | The generation of *RNA1* u18 mutant 5’ UTR by PCR-based mutagenesis |
| OZJY281F | GCCATAGAGAAAGGAGAGTTTGTGAAGATCTTCCATTCGAATC | The generation of *INU1* len0 mutant 5’ UTR by PCR-based mutagenesis |
| OZJY281R | CACAAACTCTCCTTTCTCTATGGCAATGGAAAAAAAAAATAAAAAACG | The generation of *INU1* len0 mutant 5’ UTR by PCR-based mutagenesis |
| OZJY282F | GCCATAGAGAAAGAAGAGAGTTTGTGAAGATCTTCCATTCGAATC | The generation of *INU1* len2 mutant 5’ UTR by PCR-based mutagenesis |
| OZJY282R | CACAAACTCTCTTCTTTCTCTATGGCAATGGAAAAAAAAAATAAAAAACG | The generation of *INU1* len2 mutant 5’ UTR by PCR-based mutagenesis |
| OZJY283F | TACTACTGTGTGTAACGGTTATATTTCGTTTTTTATTTTTTTTTTCCATTGCCATAGAG | The generation of *INU1* u136 mutant 5’ UTR by PCR-based mutagenesis |
| OZJY283R | ACCGTTACACACAGTAGTAATTCTTCTTTTTTTTTTTTTCTTACCCCGGATTTAATTGG | The generation of *INU1* u136 mutant 5’ UTR by PCR-based mutagenesis |
| OZJY284F | GTTAAAAAAAAAAAAAATATTTCGTTTTTTATTTTTTTTTTCCATTGCCATAGAGAAAG | The generation of *INU1* u108 mutant 5’ UTR by PCR-based mutagenesis |
| OZJY284R | ATAAAAAACGAAATATTTTTTTTTTTTTTAACCGTTACACACAGTAGTAATTC | The generation of *INU1* u108 mutant 5’ UTR by PCR-based mutagenesis |
| OZJY285F | TATTTTTTTTTTCCATTGCCATAGAGAAAGGAGAGTTTGTGAAGATCTTCCATTC | The generation of *INU1* u97 mutant 5’ UTR by PCR-based mutagenesis |
| OZJY285R | GGCAATGGAAAAAAAAAATAATTTTTTTTTTTTTAAAACGAAATATAACCGTTACACAC | The generation of *INU1* u97 mutant 5’ UTR by PCR-based mutagenesis |
| OZJY286F | ATCTTCCATTCGAAAAAAAAAAAAAAATCCCATAAGTGACACATTTAATTTTTTTTTTG | The generation of *INU1* d38 mutant 5’ UTR by PCR-based mutagenesis |
| OZJY286R | TTTCGAATGGAAGATCTTCACAAACTCTCCTTTCTCTATGGCAATGGAAAAAAAAAATA | The generation of *INU1* d38 mutant 5’ UTR by PCR-based mutagenesis |
| OZJY287F | GGAGAAACAGAGAGCGGCGATGAGTAAAGGAGAAG | The generation of *KLMA*_*60072* len1 mutant 5’ UTR by PCR-based mutagenesis |
| OZJY287R | CATCGCCGCTCTCTGTTTCTCCTTCAAATATGTTATCAAC | The generation of *KLMA*_*60072* len1 mutant 5’ UTR by PCR-based mutagenesis |
| OZJY288F | GGAGAAACAGAGAAGCGGCGATGAGTAAAGGAGAAG | The generation of *KLMA*_*60072* len2 mutant 5’ UTR by PCR-based mutagenesis |
| OZJY288R | CATCGCCGCTTCTCTGTTTCTCCTTCAAATATGTTATCAAC | The generation of *KLMA*_*60072* len2 mutant 5’ UTR by PCR-based mutagenesis |
| OZJY289F | AGTGTGTAGGGTACCAGAAAGTTGGTGTTGATAACATATTTGAAGGAGAAACAGAGGCG | The generation of *KLMA*_*60072* u78 mutant 5’ UTR by PCR-based mutagenesis |
| OZJY289R | GTACCCTACACACTACCAAACCAGTACGAATTTTTTAAGCACTATAGTACCCTGTTTCC | The generation of *KLMA*_*60072* u78 mutant 5’ UTR by PCR-based mutagenesis |
| OZJY290F | TAAAAAAATTTGAAGGAGAAACAGAGGCGGCGATGAGTAAAGG | The generation of *KLMA*_*60072* u24 mutant 5’ UTR by PCR-based mutagenesis |
| OZJY290R | CTCCTTCAAATTTTTTTATGTTATCAACACCAACTTTCTGGTAC | The generation of *KLMA*_*60072* u24 mutant 5’ UTR by PCR-based mutagenesis |
| OZJY291F | CTTTTGAATGCTCAGACCCCGGTAGAGAAATATAACCCTATTTCTAGATATAACC | The generation of *GRE2* len0 mutant 5’ UTR by PCR-based mutagenesis |
| OZJY291R | GGGTTATATTTCTCTACCGGGGTCTGAGCATTCAAAAGATAATAATAATAAATATAAC | The generation of *GRE2* len0 mutant 5’ UTR by PCR-based mutagenesis |
| OZJY292F | CTGAAACCAGAAAAGTTATTCAGTAAGTTTCACGGAAATTGATTAC | The generation of *KLMA*_*40607* len0 mutant 5’ UTR by PCR-based mutagenesis |
| OZJY292R | GAAACTTACTGAATAACTTTTCTGGTTTCAGCAAGCG | The generation of *KLMA*_*40607* len0 mutant 5’ UTR by PCR-based mutagenesis |
| OZJY293F | GTGTTGTGGAAAGGCTGCACGGTTTTTTTCATTCGCAC | The generation of *PRS3* len0 mutant 5’ UTR by PCR-based mutagenesis |
| OZJY293R | GAAAAAAACCGTGCAGCCTTTCCACAACACTAATGTG | The generation of *PRS3* len0 mutant 5’ UTR by PCR-based mutagenesis |
| OZJY294F | GCTCAGACCCCAGCTTTGCTTGAAGTATCTGTTACCCATTAGAATTATAAC | The generation of *DBP9* len0 mutant 5’ UTR by PCR-based mutagenesis |
| OZJY294R | GGGTAACAGATACTTCAAGCAAAGCTGGGGTCTGAGC | The generation of *DBP9* len0 mutant 5’ UTR by PCR-based mutagenesis |
| OZJY295F | GACCCAGTAACGTGTATCAGAGAGTATTTAACAACCAGAATTATTGAC | The generation of *PSY2* len0 mutant 5’ UTR by PCR-based mutagenesis |
| OZJY295R | GTTAAATACTCTCTGATACACGTTACTGGGTCTAAGTATTTCCTG | The generation of *PSY2* len0 mutant 5’ UTR by PCR-based mutagenesis |
| OZJY296F | GATTTCAAGAAAGAAAGCCAGGCTTGCTACTGCTAGTGAATAATTG | The generation of *OLE1* len0 mutant 5’ UTR by PCR-based mutagenesis |
| OZJY296R | CAGTAGCAAGCCTGGCTTTCTTTCTTGAAATCAAATCTATCCTTTAC | The generation of *OLE1* len0 mutant 5’ UTR by PCR-based mutagenesis |
| OZJY297F | GTTTATTCAGAGGGGTTCCGCATACTCGGCTGGATATC | The generation of *USA1* len0 mutant 5’ UTR by PCR-based mutagenesis |
| OZJY297R | CCAGCCGAGTATGCGGAACCCCTCTGAATAAACCTCTTTTGTTC | The generation of *USA1* len0 mutant 5’ UTR by PCR-based mutagenesis |
| OZJY298F | GAAGCAAATAGCAGCTTAAAGGTTTGAACTAGGTTCGGAGAC | The generation of *CDC12* len0 mutant 5’ UTR by PCR-based mutagenesis |
| OZJY298R | CTAGTTCAAACCTTTAAGCTGCTATTTGCTTCTTGCTAATTTTC | The generation of *CDC12* len0 mutant 5’ UTR by PCR-based mutagenesis |
| OZJY299F | GAGTAAAGTTGAACGAATTCAATTGAGTTGGGTTCCCAAG | The generation of *COP1* len0 mutant 5’ UTR by PCR-based mutagenesis |
| OZJY299R | CAACTCAATTGAATTCGTTCAACTTTACTCTTGAATAACACCTCAC | The generation of *COP1* len0 mutant 5’ UTR by PCR-based mutagenesis |
| OZJY300F | GAGGCTATAATCATCGGTTTACAGTTGGTTATTGAAGGTTTTTAAGTG | The generation of *SSH4* len0 mutant 5’ UTR by PCR-based mutagenesis |
| OZJY300R | CTTCAATAACCAACTGTAAACCGATGATTATAGCCTCACAAAACTGC | The generation of *SSH4* len0 mutant 5’ UTR by PCR-based mutagenesis |
| OZJY301F | CTTTTGAATGCTCAGACCCCTAGTTCAAGTAACCTGGCATCTACTG | The generation of *KLMA*_*80280* len0 mutant 5’ UTR by PCR-based mutagenesis |
| OZJY301R | CAGGTTACTTGAACTAGGGGTCTGAGCATTCAAAAGATAATAATAATAAATATAAC | The generation of *KLMA*_*80280* len0 mutant 5’ UTR by PCR-based mutagenesis |
| OZJY302F | GATGAAGAAAGGGGGGAGGCAATAACCAAGCCATAGCG | The generation of *SVF1* len0 mutant 5’ UTR by PCR-based mutagenesis |
| OZJY302R | GCTTGGTTATTGCCTCCCCCCTTTCTTCATCTCTTCTAATCAAC | The generation of *SVF1* len0 mutant 5’ UTR by PCR-based mutagenesis |
| OZJY303F | ATCGTATCTCAATCTATCTCTCTCTG | The amplification of wild-type and len0 5’ UTRs of *SSH4*, *INU1* and *KLMA_80280* |
| OZJY304R | GAGGGAGTATGCTAACTTCATGATTATTACAACACACAAACAAAAACAAAACC | The amplification of *SSH4* 5’ UTR and *SSH4* len0 5’ UTR |
| OZJY305R | GAGGGAGTATGCTAACTTCATATCTAACAAAAAAAAAATTAAATGTGTCACTTATG | The amplification of *INU1* 5’ UTR and *INU1* len0 5’ UTR |
| OZJY306R | GAGGGAGTATGCTAACTTCATCGAACAATGAATCAAAGATAGAAGAACAAAAAAATTC | The amplification of *KLMA_80280* 5’ UTR and *KLMA_80280* len0 5’ UTRs |
| OZJY307F | GCCATAGAGAAAGGAGAGTTTGTGAAGATCTTCCATTCGAATC | The generation of *INU1* len0 mutant 5’ UTR by PCR-based mutagenesis from *INU1* wild-type 5’ UTR |
| OZJY307R | CACAAACTCTCCTTTCTCTATGGCAATGGAAAAAAAAAATAAAAAACG | The generation of *INU1* len0 mutant 5’ UTR by PCR-based mutagenesis from *INU1* wild-type 5’ UTR |
| OZJY308F | GACACATTTAATAAATTTTTTTTTGTTAGATATGAAGTTAGCATACTC | The generation of *INU1* len3-d16 mutant 5’ UTR by PCR-based mutagenesis from *INU1* len0 5’ UTR |
| OZJY308R | CATATCTAACAAAAAAAAATTTATTAAATGTGTCACTTATGGGATTCGAATG | The generation of *INU1* len0-d16 mutant 5’ UTR by PCR-based mutagenesis from *INU1* len0 5’ UTR |
| OZJY309F | CATAAGTGACACATTAAAATAATTTTTTTTTTGTTAGATATGAAGTTAGCATACTC | The generation of *INU1* len4-d20 mutant 5’ UTR by PCR-based mutagenesis from *INU1* len0 5’ UTR |
| OZJY309R | CTAACAAAAAAAAAATTATTTTAATGTGTCACTTATGGGATTCGAATG | The generation of *INU1* len4-d20 mutant 5’ UTR by PCR-based mutagenesis from *INU1* len0 5’ UTR |
| OZJY310F | GAATCCCATAAGTGAAAAACACATTTAATTTTTTTTTTGTTAGATATGAAGTTAGCATACTC | The generation of *INU1* len4-d26 mutant 5’ UTR by PCR-based mutagenesis from *INU1* len0 5’ UTR |
| OZJY310R | CAAAAAAAAAATTAAATGTGTTTTTCACTTATGGGATTCGAATGGAAGATC | The generation of *INU1* len4-d26 mutant 5’ UTR by PCR-based mutagenesis from *INU1* len0 5’ UTR |
| OZJY311F | CCATAAGTGAAACACATTTAATTTTTTTTTTGTTAGATATGAAGTTAGCATACTC | The generation of *INU1* len2-d27 mutant 5’ UTR by PCR-based mutagenesis from *INU1* len0 5’ UTR |
| OZJY311R | AAAAAAAATTAAATGTGTTTCACTTATGGGATTCGAATGGAAGATC | The generation of *INU1* len2-d27 mutant 5’ UTR by PCR-based mutagenesis from *INU1* len0 5’ UTR |
| OZJY312F | GCCATAGAGAAAGAAGAGAGTTTGTGAAGATCTTCCATTCGAATC | The generation of *INU1* len2 mutant 5’ UTR by PCR-based mutagenesis from *INU1* len0 5’ UTR |
| OZJY312R | CACAAACTCTCTTCTTTCTCTATGGCAATGGAAAAAAAAAATAAAAAACG | The generation of *INU1* len2 mutant 5’ UTR by PCR-based mutagenesis from *INU1* len0 5’ UTR |
| OZJY313F | GATCTTCCATTCGAAAAATCCCATAAGTGACACATTTAATTTTTTTTTTG | The generation of *INU1* len3-d39 mutant 5’ UTR by PCR-based mutagenesis from *INU1* len0 5’ UTR |
| OZJY313R | CACTTATGGGATTTTTCGAATGGAAGATCTTCACAAACTC | The generation of *INU1* len3-d39 mutant 5’ UTR by PCR-based mutagenesis from *INU1* len0 5’ UTR |
| OZJY314F | GTTATATTTCGTTTTTAAAAAAAAAATATTTTTTTTTTCCATTGCCATAGAGAAAGG | The generation of *INU1* len8-d61 mutant 5’ UTR by PCR-based mutagenesis from *INU1* len0 5’ UTR |
| OZJY314R | CAATGGAAAAAAAAAATATTTTTTTTTTAAAAACGAAATATAACCGTTACACACAG | The generation of *INU1* len8-d61 mutant 5’ UTR by PCR-based mutagenesis from *INU1* len0 5’ UTR |
| OZJY315F | CATAGAGAAAGGAAAAAGAGTTTGTGAAGATCTTCCATTCG | The generation of *INU1* len4-d64 mutant 5’ UTR by PCR-based mutagenesis from *INU1* len0 5’ UTR |
| OZJY315R | GATCTTCACAAACTCTTTTTCCTTTCTCTATGGCAATGGA | The generation of *INU1* len4-d64 mutant 5’ UTR by PCR-based mutagenesis from *INU1* len0 5’ UTR |
| OZJY316F | GTTATATTTCGTTTTTAAAAAAAAAATATTTTTTTTTTCCATTGCCATAGAGAAAGG | The generation of *INU1* len10-u96 mutant 5’ UTR by PCR-based mutagenesis from *INU1* len0 5’ UTR |
| OZJY316R | CAATGGAAAAAAAAAATATTTTTTTTTTAAAAACGAAATATAACCGTTACACACAG | The generation of *INU1* len10-u96 mutant 5’ UTR by PCR-based mutagenesis from *INU1* len0 5’ UTR |
| OZJY317F | CCATTGCCATAAAAAAGAGAAAGGAGAGTTTGTGAAGATCTTC | The generation of *INU1* len5-u73 mutant 5’ UTR by PCR-based mutagenesis from *INU1* len0 5’ UTR |
| OZJY317R | CTCTCCTTTCTCTTTTTTATGGCAATGGAAAAAAAAAATAAAAAACG | The generation of *INU1* len5-u73 mutant 5’ UTR by PCR-based mutagenesis from *INU1* len0 5’ UTR |
| OZJY318F | CCCATAAAAAAAAAGTGACACATTTAATTTTTTTTTTGTTAGATATG | The generation of *INU1* len7-d32 mutant 5’ UTR by PCR-based mutagenesis from *INU1* len0 5’ UTR |
| OZJY318R | GTGTCACTTTTTTTTTATGGGATTCGAATGGAAGATCTTC | The generation of *INU1* len7-d32 mutant 5’ UTR by PCR-based mutagenesis from *INU1* len0 5’ UTR |
| OZJY319F | CGTTTTTTATTTTTTTAAAAAAAATTTCCATTGCCATAGAGAAAGGAG | The generation of *INU1* len8-u87 mutant 5’ UTR by PCR-based mutagenesis from *INU1* len0 5’ UTR |
| OZJY319R | GGCAATGGAAATTTTTTTTAAAAAAATAAAAAACGAAATATAACCGTTACAC | The generation of *INU1* len8-d87 mutant 5’ UTR by PCR-based mutagenesis from *INU1* len0 5’ UTR |
| OZJY320F | CAATTAAATAAAAAAAAACCGGGGTAAGGAAGAATTACTAC | The generation of *INU1* len9-u146 mutant 5’ UTR by PCR-based mutagenesis from *INU1* len0 5’ UTR |
| OZJY320R | CTTACCCCGGTTTTTTTTTATTTAATTGCTGATTAGGGGAAG | The generation of *INU1* len9-d146 mutant 5’ UTR by PCR-based mutagenesis from *INU1* len0 5’ UTR |
| GFP_RT_F | ACTCCAATTGGCGATGGCCCTGT | Used to perform qRT-PCR of *GFP* |
| GFP_RT_R | CCATGCCATGTGTAATCCCAGCAGC | Used to perform qRT-PCR of *GFP* |
| AnFaeA_RT_F | CGGCTGGATCTCCGTCCAAGACC | Used to perform qRT-PCR of *AnFaeA* |
| AnFaeA_RT_R | TGGCGGACAATTGAGCAGCGGT | Used to perform qRT-PCR of *AnFaeA* |
| SWC4_RT_F | AGCTTGCACCAGGTGTGTTCCTCA | Used to perform qRT-PCR of *SWC4* |
| SWC4_RT_R | AGGGAGAGAAGGCATGGCTGGC | Used to perform qRT-PCR of *SWC4* |

<1> lenN, where N refers to a number between 0 and 14, indicates that the longest poly(A) tract in the 5' UTR has been replaced by a continuous stretch of N adenine nucleotides.

<2> uN, where N refers to a number between 0 and 200, indicates that the longest and most downstream poly(A) tract in the 5’ UTR has been shifted upstream to a position that is N nucleotides away from the start codon.

<3> dN, where N refers to a number between 0 and 200, indicates that the longest and most downstream poly(A) tract in the 5’ UTR has been shifted downstream to a position that is N nucleotides away from the start codon.

# Table S3 Sequences of LHZ1138 and LHZ1441

| Name | Sequence (Genbank format) |
| --- | --- |
| LHZ1138 | LOCUS LHZ1138 8962 bp DNA circular 13-OCT-2023  SOURCE  ORGANISM  COMMENT This file is created by Vector NTI  http://www.invitrogen.com/  COMMENT ORIGDB\|GenBank  COMMENT VNTDATE\|-15136799\|  COMMENT VNTDBDATE\|-15136799\|  COMMENT LSOWNER\|  COMMENT VNTNAME\|LHZ1138\|  COMMENT VNTAUTHORNAME\|Yao Yu\|  FEATURES Location/Qualifiers  CDS complement(673..1383)  /vntifkey="4"  /label=mCherry  terminator complement(432..666)  /vntifkey="43"  /label=TEF\Terminator  promoter complement(1384..1762)  /vntifkey="29"  /label=TEF\Promoter  promoter 1771..2356  /vntifkey="29"  /label=KmURA3\promoter  CDS 2357..3160  /vntifkey="4"  /label=KmURA3\ORF  CDS 3193..4438  /vntifkey="4"  /label=ARS1  terminator 6526..6715  /vntifkey="43"  /label=ADH1\Terminator  CDS 5801..6517  /vntifkey="4"  /label=GFP  promoter 4444..5794  /vntifkey="29"  /label=PHXT4  BASE COUNT 2555 a 1843 c 2082 g 2482 t  ORIGIN  1 tcgcgcgttt cggtgatgac ggtgaaaacc tctgacacat gcagctcccg gagacggtca  61 cagcttgtct gtaagcggat gccgggagca gacaagcccg tcagggcgcg tcagcgggtg  121 ttggcgggtg tcggggctgg cttaactatg cggcatcaga gcagattgta ctgagagtgc  181 accatatgcg gtgtgaaata ccgcacagat gcgtaaggag aaaataccgc atcaggcgcc  241 attcgccatt caggctgcgc aactgttggg aagggcgatc ggtgcgggcc tcttcgctat  301 tacgccagct ggcgaaaggg ggatgtgctg caaggcgatt aagttgggta acgccagggt  361 tttcccagtc acgacgttgt aaaacgacgg ccagtgccaa gcttgcatgc ctgcaggtcg  421 acgatccgcg gactggatgg cggcgttagt atcgaatcga cagcagtata gcgaccagca  481 ttcacatacg attgacgcat gatattactt tctgcgcact taacttcgca tctgggcaga  541 tgatgtcgag gcgaaaaaaa atataaatca cgctaacatt tgattaaaat agaacaacta  601 caatataaaa aaactataca aatgacaagt tcttgaaaac aagaatcttt ttattgtcag  661 tactgaacta gttcacttgt acagctcgtc catgccgccg gtggagtggc ggccctcggc  721 gcgttcgtac tgttccacga tggtgtagtc ctcgttgtgg gaggtgatgt ccaacttgat  781 gttgacgttg taggcgccgg gcagctgcac gggcttcttg gccttgtagg tggtcttgac  841 ctcagcgtcg tagtggccgc cgtccttcag cttcagcctc tgcttgatct cgcccttcag  901 ggcgccgtcc tcggggtaca tccgctcgga ggaggcctcc cagcccatgg tcttcttctg  961 cattacgggg ccgtcggagg ggaagttggt gccgcgcagc ttcaccttgt agatgaactc  1021 gccgtcctgc agggaggagt cctgggtcac ggtcaccacg ccgccgtcct cgaagttcat  1081 cacgcgctcc cacttgaagc cctcggggaa ggacagcttc aagtagtcgg ggatgtcggc  1141 ggggtgcttc acgtaggcct tggagccgta catgaactga ggggacagga tgtcccaggc  1201 gaagggcagg gggccaccct tggtcacctt cagcttggcg gtctgggtgc cctcgtaggg  1261 gcggccctcg ccctcgccct cgatctcgaa ctcgtggccg ttcacggagc cctccatgtg  1321 caccttgaag cgcatgaact ccttgatgat ggccatgtta tcctcctcgc ccttgctcac  1381 catggttgtt tatgttcgga tgtgatgtga gaactgtatc ctagcaagat tttaaaagga  1441 agtatatgaa agaagaacct cagtggcaaa tcctaacctt ttatatttct ctacaggggc  1501 gcggcgtggg gacaattcaa cgcgtctgtg aggggagcgt ttccctgctc gcaggtctgc  1561 agcgaggagc cgtaattttt gcttcgcgcc gtgcggccat caaaatgtat ggatgcaaat  1621 gattatacat ggggatgtat gggctaaatg tacgggcgac agtcacatca tgcccctgag  1681 ctgcgcacgt caagactgtc aaggagggta ttctgggcct ccatgtcgct ggccgggtga  1741 cccggcgggg acaaggcaag ctttaattaa cgaattctga ttggaaagac cattctgctt  1801 tacttttaga gcatcttggt cttctgagct cattatacct caatcaaaac tgaaattagg  1861 tgcctgtcac ggctcttttt ttactgtacc tttgacttcc tttcttattt ccaaggatgc  1921 tcatcacaat acgcttctag atctattatg cattataatt aatagttgta gctacaaaag  1981 gtaaaagaaa gtccggggca ggcaacaata gaaatcggca aaaaaaacta cagaaatact  2041 aagagcttct tccccattca gtcatcgcat ttcgaaacaa gaggggaatg gctctggcta  2101 gggaactaac caccatcgac tgactctatg cactaaccac gtgactacat atatgtgatc  2161 gtttttaaca tttttcaaag gctgtgtgtc tggctgtttc cattaatttt cactgattaa  2221 gcagtcatat tgaatctgag ctcatcacca acaagaaatt ctaccgtaaa agtgtaaaag  2281 ttcgtttaaa tcatttgtaa actggaacag caagaggaag tatcatcagc tagccccata  2341 aactaatcaa aggaggatgt cgactaagag ttactcggaa agagcagctg ctcatagaag  2401 tccagttgct gccaagcttt taaacttgat ggaagagaag aagtcaaact tatgtgcttc  2461 tcttgatgtt cgtaaaacag cagagttgtt aagattagtt gaggttttgg gtccatatat  2521 ctgtctattg aagacacatg tagatatctt ggaggatttc agctttgaga ataccattgt  2581 gccgttgaag caattagcag agaaacacaa gtttttgata tttgaagaca ggaagtttgc  2641 cgacattggg aacactgtta aattacaata cacgtctggt gtataccgta tcgccgaatg  2701 gtctgatatc accaatgcac acggtgtgac tggtgcgggc attgttgctg gtttgaagca  2761 aggtgccgag gaagttacaa aagaacctag agggttgtta atgcttgccg agttatcgtc  2821 caaggggtct ctagcgcacg gtgaatacac tcgtgggacc gtggaaattg ccaagagtga  2881 taaggacttt gttattggat ttattgctca aaacgatatg ggtggaagag aagagggcta  2941 cgattggttg atcatgacgc caggtgttgg tcttgatgac aaaggtgatg ctttgggaca  3001 acaatacaga actgtggatg aagttgttgc cggtggatca gacatcatta ttgttggtag  3061 aggtcttttc gcaaagggaa gagatcctgt agtggaaggt gagagataca gaaaggcggg  3121 atgggacgct tacttgaaga gagtaggcag atccgcttaa gaggggccga gctcggggat  3181 cctctagaga ttatcgattg aagttttgtc caactatcca ctatggatat gcgttttgtt  3241 gattaacctt aaataacacg tatttcgcat tttccaaaag ccttttttca taactacaaa  3301 ctaactattt ttgtttattt tacatcagta aattatgcgc agaaaatatg taaggctata  3361 tactcaatat agtggaagag cctctggcta cttaatctgg gttcatatat tctgtcagtg  3421 gtatagtaaa tttaaatagt gaatttgggc gcatgtagag agatcttttg attaataacc  3481 cagtatttga ttcttgaatg ttatttgtct cttattaagt atttcagttt gatttatata  3541 ttttgaaagt aattgttgct ttaccttcca aacaataaaa aaatataaaa aaatgtaaaa  3601 aatataataa atattaaata aaatactact tgtttgaaaa tcagagaaaa tccaccaaaa  3661 tatcaatcat ttcaaggatt tccgaaccaa gttcgagata gtcctttaag gtcagtaaaa  3721 ttcagttgca cgtataacag atatttttca ttttgttcca atttaaaagt cccccatttt  3781 taaaattatt gaaaataaaa attaaaaaat taaaaggaat ctctctatgt cactttaaaa  3841 taaataaatt gaaaatgata tatcgttaaa agtcaaccgc aacaccacct atttctaaga  3901 ggagagttct aaaaaaatca tagtaccaca caggtaaact aaaacagcta aattcaacat  3961 aatacctgtg gatataatta catacaaaaa tataattaaa aaaatacatt aaaaataatt  4021 tatttttgta aaacccataa aatatatttt actttcggaa caacttttta acttataatt  4081 ttgttttaaa taaaaacgtt gtatttaaaa ataataaaat attaagtaaa aatttaaagc  4141 atatttattt aaaatataaa atactactaa aattactcta aacttcaaaa taaaaaaata  4201 ataaattata tagttttaaa attaataatt tgtatacacg tgaccagacc ataatagttt  4261 tcttttcttg aaactgccat gaatttaata gatttttttt acacatattt acagtaagtt  4321 ttgtttttac gttgaattaa tattgtatac gtacttagaa ggataacttc caactatgaa  4381 tatgtaggtt aaaaggtaaa tagagaagcc ttacttttat cagaaatgaa aggagctcgg  4441 atcctgtatt ctacctgtaa aaaaaaaccg tcaaaaaaaa caaacaacaa caacaaaaac  4501 caaaacccaa tctacgattg tttttccgtg gggttccttc tgacctcttc caggcccttt  4561 ctacggccct ttattccggt ggtgtgaagc gcttctccat agatcccgac ataccgcttc  4621 ttcggatttt tgacatggct aaaaaaatac caaaaaaaaa aaaacaggct gtaataggaa  4681 agggggagaa caattgtttt tccggggtgg gggggctctt tactgcgtgt tttctgggga  4741 aaatatgcgg tggggtgatg ggtttctcca gaatatttta ggcggcaggt cttatctcct  4801 catttttttt ttcacccacg cgcgcgtatg tgtggttgtg tgtggttgtg tttgtttgtg  4861 cttgtttgtt tgtttgtgtg tgccacgggt taaaaaggaa aaaaaaattg aaaattgagg  4921 acaaactttt tccgagtccc tactattttt ctggggaaaa ttcggatggg gtgatgaatt  4981 cgaggtttct ccacattgtg ttgttgtttt ggtgccgaag atggtaatta cagcaaaatt  5041 aaaaaaaaaa aatgcattag ataaaattta ttatttttgt ttgagctttt ttaaggcaca  5101 gattctgccg cacagggcgc acacagggcg cgcccgcgct tacggtaata gccctggaaa  5161 agtccaggaa tagccacgga tggagaaaaa gaaacaacgg aaaatctttg ggggtggttt  5221 tagtaggcgg tgcacgggtg ggatcatgcg tttttgtcga gcgatgccgg aagaatctgg  5281 ggaaagcaga gttcccggcg tgagatatgg agtggaagcg cgcgtagacg cgggcgcagt  5341 gaagaaagag ggagagcaag tgctgcagta aaataggaag tagcaggtaa caagatctga  5401 gctggattgg ttagggtagt tgttccgata cgggggtgtc tgcgggcgaa aaaaaaaact  5461 gggaccatta cattgtccaa caatagtcac atcacactat gaaatttttt taactaatta  5521 gtgtattgtt ccggaaatag tgtgtatgtg tgagagtatt ttcgcttggc ttacttggct  5581 acttggcacc ggctgctaga ttctggtgcc aaggtggtgc caaggtgtcc aagagaagaa  5641 gcgttagcag acgctaaagg tgaacgggtt ttcgccatga attttctgat ataaaaggaa  5701 gtggtttgga actgaaattt gaaattggaa tcgtatctca atctatctct ctctgttata  5761 tttattatta ttatcttttg aatgctcaga ccccctcgag atgagtaaag gagaagaact  5821 tttcactgga gttgtcccaa ttcttgttga attagatggt gatgttaatg ggcacaaatt  5881 ttctgtcagt ggagagggtg aaggtgatgc aacatacgga aaacttaccc ttaaatttat  5941 ttgcactact ggaaaactac ctgttccatg gccaacactt gtcactactc tcacttatgg  6001 tgttcaatgc ttttcaagat acccagatca catgaaacag catgactttt tcaagagtgc  6061 catgcccgaa ggttatgtac aggaaagaac tatatttttc aaagatgacg ggaactacaa  6121 gacacgtgct gaagtcaagt ttgaaggtga tacccttgtt aatagaatcg agttaaaagg  6181 tattgatttt aaagaagatg gaaacattct tggacacaaa ttggaataca actataactc  6241 acacaatgta tacatcatgg cagacaaaca aaagaatgga atcaaagtta acttcaaaat  6301 tagacacaac attgaagatg gaagcgttca actagcagac cattatcaac aaaatactcc  6361 aattggcgat ggccctgtcc ttttaccaga caaccattac ctgtccacac aatctgccct  6421 ttcgaaagat cccaacgaaa agagagacca catggtcctt cttgagtttg taacagctgc  6481 tgggattaca catggcatgg atgaactata caaataggcg gccgcgcgaa tttcttatga  6541 tttatgattt ttattattaa ataagttata aaaaaaataa gtgtatacaa attttaaagt  6601 gactcttagg ttttaaaacg aaaattctta ttcttgagta actctttcct gtaggtcagg  6661 ttgctttctc aggtatagta tgaggtcgct cttattgacc acacctctac cggcataccg  6721 agctcgaatt cgtaatcatg gtcatagctg tttcctgtgt gaaattgtta tccgctcaca  6781 attccacaca acatacgagc cggaagcata aagtgtaaag cctggggtgc ctaatgagtg  6841 agctaactca cattaattgc gttgcgctca ctgcccgctt tccagtcggg aaacctgtcg  6901 tgccagctgc attaatgaat cggccaacgc gcggggagag gcggtttgcg tattgggcgc  6961 tcttccgctt cctcgctcac tgactcgctg cgctcggtcg ttcggctgcg gcgagcggta  7021 tcagctcact caaaggcggt aatacggtta tccacagaat caggggataa cgcaggaaag  7081 aacatgtgag caaaaggcca gcaaaaggcc aggaaccgta aaaaggccgc gttgctggcg  7141 tttttccata ggctccgccc ccctgacgag catcacaaaa atcgacgctc aagtcagagg  7201 tggcgaaacc cgacaggact ataaagatac caggcgtttc cccctggaag ctccctcgtg  7261 cgctctcctg ttccgaccct gccgcttacc ggatacctgt ccgcctttct cccttcggga  7321 agcgtggcgc tttctcatag ctcacgctgt aggtatctca gttcggtgta ggtcgttcgc  7381 tccaagctgg gctgtgtgca cgaacccccc gttcagcccg accgctgcgc cttatccggt  7441 aactatcgtc ttgagtccaa cccggtaaga cacgacttat cgccactggc agcagccact  7501 ggtaacagga ttagcagagc gaggtatgta ggcggtgcta cagagttctt gaagtggtgg  7561 cctaactacg gctacactag aagaacagta tttggtatct gcgctctgct gaagccagtt  7621 accttcggaa aaagagttgg tagctcttga tccggcaaac aaaccaccgc tggtagcggt  7681 ggtttttttg tttgcaagca gcagattacg cgcagaaaaa aaggatctca agaagatcct  7741 ttgatctttt ctacggggtc tgacgctcag tggaacgaaa actcacgtta agggattttg  7801 gtcatgagat tatcaaaaag gatcttcacc tagatccttt taaattaaaa atgaagtttt  7861 aaatcaatct aaagtatata tgagtaaact tggtctgaca gttaccaatg cttaatcagt  7921 gaggcaccta tctcagcgat ctgtctattt cgttcatcca tagttgcctg actccccgtc  7981 gtgtagataa ctacgatacg ggagggctta ccatctggcc ccagtgctgc aatgataccg  8041 cgagacccac gctcaccggc tccagattta tcagcaataa accagccagc cggaagggcc  8101 gagcgcagaa gtggtcctgc aactttatcc gcctccatcc agtctattaa ttgttgccgg  8161 gaagctagag taagtagttc gccagttaat agtttgcgca acgttgttgc cattgctaca  8221 ggcatcgtgg tgtcacgctc gtcgtttggt atggcttcat tcagctccgg ttcccaacga  8281 tcaaggcgag ttacatgatc ccccatgttg tgcaaaaaag cggttagctc cttcggtcct  8341 ccgatcgttg tcagaagtaa gttggccgca gtgttatcac tcatggttat ggcagcactg  8401 cataattctc ttactgtcat gccatccgta agatgctttt ctgtgactgg tgagtactca  8461 accaagtcat tctgagaata gtgtatgcgg cgaccgagtt gctcttgccc ggcgtcaata  8521 cgggataata ccgcgccaca tagcagaact ttaaaagtgc tcatcattgg aaaacgttct  8581 tcggggcgaa aactctcaag gatcttaccg ctgttgagat ccagttcgat gtaacccact  8641 cgtgcaccca actgatcttc agcatctttt actttcacca gcgtttctgg gtgagcaaaa  8701 acaggaaggc aaaatgccgc aaaaaaggga ataagggcga cacggaaatg ttgaatactc  8761 atactcttcc tttttcaata ttattgaagc atttatcagg gttattgtct catgagcgga  8821 tacatatttg aatgtattta gaaaaataaa caaatagggg ttccgcgcac atttccccga  8881 aaagtgccac ctgacgtcta agaaaccatt attatcatga cattaaccta taaaaatagg  8941 cgtatcacga ggccctttcg tc  // |
|  |  |
| LHZ1441 | LOCUS LHZ1441 12454 bp DNA circular 13-OCT-2023  SOURCE  ORGANISM  COMMENT This file is created by Vector NTI  http://www.invitrogen.com/  COMMENT ORIGDB\|GenBank  COMMENT VNTDATE\|-15136799\|  COMMENT VNTDBDATE\|-15136799\|  COMMENT LSOWNER\|  COMMENT VNTNAME\|LHZ1441\|  COMMENT VNTAUTHORNAME\|Yao Yu\|  FEATURES Location/Qualifiers  terminator 10186..11027  /vntifkey="43"  /label=Inulinase\Terminator  promoter 11044..11629  /vntifkey="29"  /label=KcURA3\Promoter  CDS 11630..12433  /vntifkey="4"  /label=KcURA3\ORF  misc_feature 3112..7868  /vntifkey="21"  /label=KD  misc_feature 1..3103  /vntifkey="21"  /label=pKS  CDS 9316..10158  /vntifkey="4"  /label=AnFaeA\optimized  sig_peptide 9232..9300  /vntifkey="94"  /label=Inulinase\Signal\Peptide  promoter 7875..9225  /vntifkey="29"  /label=KmHXT4\Promoter  BASE COUNT 3458 a 2738 c 2798 g 3460 t  ORIGIN  1 cgtaatcatg tcatagctgt ttcctgtgtg aaattgttat ccgctcacaa ttccacacaa  61 catacgagcc ggaagcataa agtgtaaagc ctggggtgcc taatgagtga gctaactcac  121 attaattgcg ttgcgctcac tgcccgcttt ccagtcggga aacctgtcgt gccagctgca  181 ttaatgaatc ggccaacgcg cggggagagg cggtttgcgt attgggcgct cttccgcttc  241 ctcgctcact gactcgctgc gctcggtcgt tcggctgcgg cgagcggtat cagctcactc  301 aaaggcggta atacggttat ccacagaatc aggggataac gcaggaaaga acatgtgagc  361 aaaaggccag caaaaggcca ggaaccgtaa aaaggccgcg ttgctggcgt ttttccatag  421 gctccgcccc cctgacgagc atcacaaaaa tcgacgctca agtcagaggt ggcgaaaccc  481 gacaggacta taaagatacc aggcgtttcc ccctggaagc tccctcgtgc gctctcctgt  541 tccgaccctg ccgcttaccg gatacctgtc cgcctttctc ccttcgggaa gcgtggcgct  601 ttctcatagc tcacgctgta ggtatctcag ttcggtgtag gtcgttcgct ccaagctggg  661 ctgtgtgcac gaaccccccg ttcagcccga ccgctgcgcc ttatccggta actatcgtct  721 tgagtccaac ccggtaagac acgacttatc gccactggca gcagccactg gtaacaggat  781 tagcagagcg aggtatgtag gcggtgctac agagttcttg aagtggtggc ctaactacgg  841 ctacactaga agaacagtat ttggtatctg cgctctgctg aagccagtta ccttcggaaa  901 aagagttggt agctcttgat ccggcaaaca aaccaccgct ggtagcggtg gtttttttgt  961 ttgcaagcag cagattacgc gcagaaaaaa aggatctcaa gaagatcctt tgatcttttc  1021 tacggggtct gacgctcagt ggaacgaaaa ctcacgttaa gggattttgg tcatgagatt  1081 atcaaaaagg atcttcacct agatcctttt aaattaaaaa tgaagtttta aatcaatcta  1141 aagtatatat gagtaaactt ggtctgacag ttaccaatgc ttaatcagtg aggcacctat  1201 ctcagcgatc tgtctatttc gttcatccat agttgcctga ctccccgtcg tgtagataac  1261 tacgatacgg gagggcttac catctggccc cagtgctgca atgataccgc gagacccacg  1321 ctcaccggct ccagatttat cagcaataaa ccagccagcc ggaagggccg agcgcagaag  1381 tggtcctgca actttatccg cctccatcca gtctattaat tgttgccggg aagctagagt  1441 aagtagttcg ccagttaata gtttgcgcaa cgttgttgcc attgctacag gcatcgtggt  1501 gtcacgctcg tcgtttggta tggcttcatt cagctccggt tcccaacgat caaggcgagt  1561 tacatgatcc cccatgttgt gcaaaaaagc ggttagctcc ttcggtcctc cgatcgttgt  1621 cagaagtaag ttggccgcag tgttatcact catggttatg gcagcactgc ataattctct  1681 tactgtcatg ccatccgtaa gatgcttttc tgtgactggt gagtactcaa ccaagtcatt  1741 ctgagaatag tgtatgcggc gaccgagttg ctcttgcccg gcgtcaatac gggataatac  1801 cgcgccacat agcagaactt taaaagtgct catcattgga aaacgttctt cggggcgaaa  1861 actctcaagg atcttaccgc tgttgagatc cagttcgatg taacccactc gtgcacccaa  1921 ctgatcttca gcatctttta ctttcaccag cgtttctggg tgagcaaaaa caggaaggca  1981 aaatgccgca aaaaagggaa taagggcgac acggaaatgt tgaatactca tactcttcct  2041 ttttcaatat tattgaagca tttatcaggg ttattgtctc atgagcggat acatatttga  2101 atgtatttag aaaaataaac aaataggggt tccgcgcaca tttccccgaa aagtgccacc  2161 tgacgtctaa gaaaccatta ttatcatgac attaacctat aaaaataggc gtatcacgag  2221 gccctttcgt ctcgcgcgtt tcggtgatga cggtgaaaac ctctgacaca tgcagctccc  2281 ggagacggtc acagcttgtc tgtaagcgga tgccgggagc agacaagccc gtcagggcgc  2341 gtcagcgggt gttggcgggt gtcggggctg gcttaactat gcggcatcag agcagattgt  2401 actgagagtg caccataaaa ttgtaaacgt taatattttg ttaaaattcg cgttaaattt  2461 ttgttaaatc agctcatttt ttaaccaata ggccgaaatc ggcaaaatcc cttataaatc  2521 aaaagaatag cccgagatag ggttgagtgt tgttccagtt tggaacaaga gtccactatt  2581 aaagaacgtg gactccaacg tcaaagggcg aaaaaccgtc tatcagggcg atggcccact  2641 acgtgaacca tcacccaaat caagtttttt ggggtcgagg tgccgtaaag cactaaatcg  2701 gaaccctaaa gggagccccc gatttagagc ttgacgggga aagccggcga acgtggcgag  2761 aaaggaaggg aagaaagcga aaggagcggg cgctagggcg ctggcaagtg tagcggtcac  2821 gctgcgcgta accaccacac ccgccgcgct taatgcgccg ctacagggcg cgtactatgg  2881 ttgctttgac gtatgcggtg tgaaataccg cacagatgcg taaggagaaa ataccgcatc  2941 aggcgccatt cgccattcag gctgcgcaac tgttgggaag ggcgatcggt gcgggcctct  3001 tcgctattac gccagctggc gaaaggggga tgtgctgcaa ggcgattaag ttgggtaacg  3061 ccagggtttt cccagtcacg acgttgtaaa acgacggcca gtgccaagct tgcatgcatc  3121 actaatgaaa agcatacgac gcctgcgtct gacatgcact cattctgaag aagattctgg  3181 gcgcgtttcg ttctcgtttt cctctgtata ttgtactctg gtggacaatt tgaacataac  3241 gtctttcacc tcgccattct caataatggg ttccaattct atccaggtag cggttaattg  3301 acggtgctta agccgtatgc tcactctaac gctaccgttg tccaaacaac ggaccccttt  3361 gtgacgggtg taagacccat catgaagtaa aacatctcta acggtatgga aaagagtggt  3421 acggtcaagt ttcctggcac gagtcaattt tccctcttcg tgtagatcag aggctatata  3481 catgccgagg tattcgatca ctctacgatg acggtctgtt agctcaacaa cttcttctaa  3541 atgctccata accgtaacgt aagaagcata actgtcaata ctgaagtcat cccagtttat  3601 tggtgctcct gttgaacagt catccactat atgttcgaat agcccaggat cacgaggagg  3661 tcctacaaac ggatacggta cagtcttctt tttatagtct gcaaattcta gaatagcatt  3721 ttttatccaa tagtgtcgaa tcgtcctggc cgttctaccg ataaaggatc caatgtgatt  3781 attagctcca ctacacgata tgttaagttt gatcgatgtc ttgttaacaa acgctaaact  3841 caagttcggc atttccaaca gcgagaagaa atcatcaatt ccatcggcta tctcttgata  3901 agtcattaga tcatatacct tctcgggatg tcgttgagtt actttatgac tagaaatctt  3961 caggttatca tcaacgtaat tgttctccaa tagctctgga gagggacata acaatacttt  4021 gattttttcc atggcctgga cttgtttccg taggaaatac ttgttctttt gtagacgttc  4081 catgatgagt ttgtatacct ctgctggaga tatccattct agatctttga tataagtttg  4141 gtatggtaaa gagttgattt tgtaggacac gtaaatctgc gctagataag tacattgtgc  4201 aaatgcctct ggtacttcgt aagacccatg ctgcgtaatt atagtattat tgagtggatc  4261 ataagcgttg tactcgtttt tgaatttaaa actgtctaat aaggccctgt aaatctctct  4321 gacttgttgt acacctttct gctcttcggg actgagatcg gatagcaatg gagcagcagt  4381 ttctgagctt tctgatgggg ctgacatggc agatgcctat tcaatgctgc cttttgtttg  4441 ggaggttatg aaatgcatct gtttacattg tatgtaatac ccttactagg caatgttata  4501 agcaaaaatc ctttgatcac atggaatatc actttatacg tgttgaaata tgcaaaaaaa  4561 cagtccccct gagctcaggg ggtggtttac gcttttgagg ctcagcagcg cgaattctct  4621 cttggggctg aagtgaaatt taaaaaagtc gcttgaggct cagccggaat tataaaacat  4681 cacctgagtc ttgagagcgc tttcactcac ctgaggctca gctgaaattt caaaaagtca  4741 cttgagccca gaaggagtgt ttcaccccct gaggctataa cgttcgttat tttaatacct  4801 aaataaacaa aaatatatgg tacaggaacg cgaggcaacg cgccgataca gggtcaatgg  4861 gtacacgaga gggtgacact aggcgtagaa agtcattagt ataaaataca gtggtatata  4921 gtagatattt agtttgtttt ccttttcttt ttctccaaaa cgatatcaga catttgtctg  4981 ataatgaagc attatcagac aaatgtctga tatcgttttt caataataat atacatcatc  5041 acaaaacaaa caaacatagc atcgcaagcc ccatcatgcc accaccgtcc gctgtgatcg  5101 caactcatgt ttccggcggt attctgcaat gaattggaga acctcgtctg agataattcc  5161 atgccattgt tcgaacaact ggaggctagg atgagctgag aaggattgag cgaccaagcg  5221 cggacttgac ggtgggctga gtggtgggct accagggctg ttaccctcct cttcaagtag  5281 ctcctcgcga gataaaggtt tattagaagg atccttcaaa acatatattt cactgcccaa  5341 tggggcttcc ttgtaaaaac ctgatataaa ggcaaataca cggtcatcta cagtcacact  5401 accatgactg tagtgtgatc tagccactgc actttgaatt tcacggtccc cagcccaatt  5461 gcccagtgag gagacatatt ttcccttctc agatctcgat aaaaaggtcg ccattaaatg  5521 tcttccaaaa tgagacttcg ggccgttcca gatcttgaag actggttcat cgacatgctg  5581 ggtaagaaac ctagagaacg ttctggccaa tgactctggt aaaaactgat gagtttgatt  5641 agttggtcta ttactggata ctgttttttc aataggcgag caaacacgca aatagtcgta  5701 taatgatatc agaagatcgc aatcaccatt cacaggatag aagttaacgt accgttcagt  5761 tctgcttttc gtttctgtca cagtagcacg cacaattggg cccagaaatg aattgttgta  5821 gatctcaaaa gtccttggat ctagattctt cagatcgctg tatctgcagc aatttccaac  5881 agctcccaga agtagcaatc ggtattccgc tcgttttgta gtggttacgc aggactgatc  5941 gaagaagcag gcaatcctgg agacaatctt ccaaatatct ttttcttttg acagaatatt  6001 agtgaattgt aatccaacca tagaagcatc gtatttatgt gtttcctcgt agcgatcaaa  6061 caaggaaact tcttgatttt taaatgggct aacaacaacc ttgtaagaag gcaatgctga  6121 ttcgatatcc ttttgcagag actctgtctt tcttagtcta acagtgaatt tgataatttt  6181 gtcatcctta tcaaaagaca gagatttgcc aattgcgctc ttgtaagagc ggtaggtatt  6241 gattttcatc tcgcgtcgga tagatagcga ctgcattgtc aagatagaga atagggacgc  6301 cagcttattt ctaatttctt tcgcatttat attaaaggtg tcagattcca gaatttcatt  6361 aatttcatta gcacactgat gaggtgtgag gtgagcagcc tccgcaaagg tagacatagg  6421 ggcattggtt ggaggccttt gaggtaccac tagagtgctg caaacatagc accgttcgag  6481 actttaaaat cttcagtttt aaaattatga aaaaaaacat cgtcctgagt tgaaacggtc  6541 gtttcaacct ccgtgtacag aaagatacat agcatatggc aagctgcacg cagcgtaaac  6601 atgccggaca actgtcattt cgtcagatca gttgatctac tctctgtgat actgcttcgt  6661 ttgtccacgg aggtcggact aactctcacc acgcttccac ggcattcgaa agaactaata  6721 ttgtatcatt gtacatatga ggaacacgca gttgaactga gcaaaccagg actcaggaaa  6781 gcaggaggta agtgctcgct tttcgtggat ccagaggaac gtgaaaattc gccttctcct  6841 cctataccgc cgtatcagat atcagagatg ccccttcatg aacttctcga gtcaggcaat  6901 gctaaattgg ttccaaatcc cgagtttgat ctaactgatc cagacgactt tcataagtgt  6961 ttctcggtca cctattcagc attatcttta atggtaccat atctgcccag agctgctcta  7021 aaggctgctc gagtgttttg taaagatcat tcaatattaa caacggatat gcttgatttg  7081 aattatcttg aagagctaat tgagttctca aaggaaactg tgaacaaaat cccagctaga  7141 atccctatag aggacatgct tctcgagcgg ggatatgtgc taccatgggt tcatggtggt  7201 acagtgaagg gaggaaagct actgaccccc aacgattgat tctttaccga atcattgcat  7261 aattcattgc ataattcatt gcagaatacc gccggaaaca tgagttgcga tcacagcgga  7321 cggtggtggc atgatggggc ttgcgatgct atgtttgttt gttttgtgat gatgtatatt  7381 attattgaaa aacgatatca gacatttgtc tgataatgct tcattatcag acaaatgtct  7441 gatatcgttt tggagaaaaa gaaaaggaaa acaaactaaa tatctactat ataccactgt  7501 attttatact aatgactttc tacgcctagt gtcaccctct cgtgtaccca ttgaccctgt  7561 atcggcgcgt tgcctcgcgt tcctgtacca tatatttttg tttatttagg tattaaaatt  7621 tactttcctc atacaaatat taaattcacc aaacttctca aaaactaatt attcgtagtt  7681 acaaactcta ttttacaatc acgtttattc aaccattcta catccaataa ccaaaatgcc  7741 catgtacctc tcagcgaagt ccaacggtac tgtccaatat tctcattaaa tagtctttca  7801 tctatatatc agaaggtaat tataattaga gatttcgaat cattaccgtg ccgattcgca  7861 cgctgcaacc gcggctgtat tctacctgta aaaaaaaacc gtcaaaaaaa acaaacaaca  7921 acaacaaaaa ccaaaaccca atctacgatt gtttttccgt ggggttcctt ctgacctctt  7981 ccaggccctt tctacggccc tttattccgg tggtgtgaag cgcttctcca tagatcccga  8041 cataccgctt cttcggattt ttgacatggc taaaaaaata ccaaaaaaaa aaaaacaggc  8101 tgtaatagga aagggggaga acaattgttt ttccggggtg ggggggctct ttactgcgtg  8161 ttttctgggg aaaatatgcg gtggggtgat gggtttctcc agaatatttt aggcggcagg  8221 tcttatctcc tcattttttt tttcacccac gcgcgcgtat gtgtggttgt gtgtggttgt  8281 gtttgtttgt gcttgtttgt ttgtttgtgt gtgccacggg ttaaaaagga aaaaaaaatt  8341 gaaaattgag gacaaacttt ttccgagtcc ctactatttt tctggggaaa attcggatgg  8401 ggtgatgaat tcgaggtttc tccacattgt gttgttgttt tggtgccgaa gatggtaatt  8461 acagcaaaat taaaaaaaaa aaatgcatta gataaaattt attatttttg tttgagcttt  8521 tttaaggcac agattctgcc gcacagggcg cacacagggc gcgcccgcgc ttacggtaat  8581 agccctggaa aagtccagga atagccacgg atggagaaaa agaaacaacg gaaaatcttt  8641 gggggtggtt ttagtaggcg gtgcacgggt gggatcatgc gtttttgtcg agcgatgccg  8701 gaagaatctg gggaaagcag agttcccggc gtgagatatg gagtggaagc gcgcgtagac  8761 gcgggcgcag tgaagaaaga gggagagcaa gtgctgcagt aaaataggaa gtagcaggta  8821 acaagatctg agctggattg gttagggtag ttgttccgat acgggggtgt ctgcgggcga  8881 aaaaaaaaac tgggaccatt acattgtcca acaatagtca catcacacta tgaaattttt  8941 ttaactaatt agtgtattgt tccggaaata gtgtgtatgt gtgagagtat tttcgcttgg  9001 cttacttggc tacttggcac cggctgctag attctggtgc caaggtggtg ccaaggtgtc  9061 caagagaaga agcgttagca gacgctaaag gtgaacgggt tttcgccatg aattttctga  9121 tataaaagga agtggtttgg aactgaaatt tgaaattgga atcgtatctc aatctatctc  9181 tctctgttat atttattatt attatctttt gaatgctcag acccccccgg gatgaagtta  9241 gcatactccc tcttgcttct attggcagga gtcagtgctt cagtgatcaa ttacaagaga  9301 gacggtgaca ctagtaagca attctccgct aagtacgctt tgatcttgtt ggccactgct  9361 ggtcaagctt tggctgcctc tacccaaggt atctccgaag acttgtacaa cagattggtc  9421 gagatggcca ccatctctca agccgcctac gctgacttat gcaacatccc atccaccatc  9481 atcaagggtg aaaagattta caacgcccag accgatatca acggttggat cttgagagac  9541 gacacctcca aggagatcat caccgtcttc agaggtaccg gttccgacac caacttgcag  9601 ttggacacca actacacctt gaccccattc gacaccttgc cacaatgcaa cgactgtgaa  9661 gtccacggtg gttactatat cggctggatc tccgtccaag accaagtcga gtccttggtc  9721 aagcagcaag cttcccaata ccccgattac gctttgaccg tcaccggtca ttctttgggt  9781 gcctctatgg ctgctttgac cgctgctcaa ttgtccgcca cctacgacaa cgtcagattg  9841 tacaccttcg gcgagccaag atctggtaac caagctttcg cctcctacat gaacgacgcc  9901 ttccaagttt cctctccaga gaccacccag tacttcagag ttacccactc caacgacggc  9961 atcccaaact tgccaccagc tgacgaaggt tacgctcatg gtggcgttga atactggtcc  10021 gtcgatccat actctgccca aaacaccttc gtctgtaccg gtgatgaggt ccaatgctgt  10081 gaggctcaag gtggtcaagg tgttaacgac gcccacacca cctacttcgg tatgacctcc  10141 ggtgcttgta cttggtaagc ggccgcttaa ggccgcaagc tttgatctga tctgcttact  10201 ttactaacga caaaaaaaaa tcaaaaaaaa aaaaacaatc agtccttctc ttcttacgat  10261 atgatatgat taaatgatgc tatgaaatca tcttcttctt aactttctta aatcttacgc  10321 gtcacttact ctatataccc gtttagcttt gcctggtcac agcgacattt tatataagtg  10381 tacgtatttt cttttttttt ttaaaaattt ctattctaac cttagaaaag tgccctttaa  10441 accagctgtc ctggcactat atctttatca tgtgccggtc gctttccctt tccgtttccc  10501 ttttcctttc aattggtggc ctggaattcc gaactcattt tcgcatctga aactaattct  10561 cgaaaccttt aacatcaaac aattgaaaag atcatcatca ccagaaataa gaaaaagatc  10621 aacacaacag ctaataacag tacgaaagaa agatcgctcg agtgaaaagg cagccaagaa  10681 aggtcattcg atttgggtct agactgatta tagacatacc aattgcactc agtaagaaaa  10741 tgagtttcaa atttgacgat gacggtgtgg taaaagaatt tcacggcaac accatcatat  10801 gccatattcc tcaacaaacc gaattcttca acaaattgtt ggacttctac cgttttgcga  10861 aacgactttc cttctacgac aagatcaccc tacttcctcc ttcaagctac cacgttacga  10921 tcatgaattg ctgccacgaa cacgatcgtt ctgagggcca ctggcccaaa ggaatcgatc  10981 cggacacaag catgctgcgg tgtacatcac atctgaccaa cattctatta attaaggatc  11041 ggtcgaattc tgattggaaa gaccattctg ctttactttt agagcatctt ggtcttctga  11101 gctcattata cctcaatcaa aactgaaatt aggtgcctgt cacggctctt tttttactgt  11161 acctttgact tcctttctta tttccaagga tgctcatcac aatacgcttc tagatctatt  11221 atgcattata attaatagtt gtagctacaa aaggtaaaag aaagtccggg gcaggcaaca  11281 atagaaatcg gcaaaaaaaa ctacagaaat actaagagct tcttccccat tcagtcatcg  11341 catttcgaaa caagagggga atggctctgg ctagggaact aaccaccatc gactgactct  11401 atgcactaac cacgtgacta catatatgtg atcgttttta acatttttca aaggctgtgt  11461 gtctggctgt ttccattaat tttcactgat taagcagtca tattgaatct gagctcatca  11521 ccaacaagaa attctaccgt aaaagtgtaa aagttcgttt aaatcatttg taaactggaa  11581 cagcaagagg aagtatcatc agctagcccc ataaactaat caaaggagga tgtcgactaa  11641 gagttactcg gaaagagcag ctgctcatag aagtccagtt gctgccaagc ttttaaactt  11701 gatggaagag aagaagtcaa acttatgtgc ttctcttgat gttcgtaaaa cagcagagtt  11761 gttaagatta gttgaggttt tgggtccata tatctgtcta ttgaagacac atgtagatat  11821 cttggaggat ttcagctttg agaataccat tgtgccgttg aagcaattag cagagaaaca  11881 caagtttttg atatttgaag acaggaagtt tgccgacatt gggaacactg ttaaattaca  11941 atacacgtct ggtgtatacc gtatcgccga atggtctgat atcaccaatg cacacggtgt  12001 gactggtgcg ggcattgttg ctggtttgaa gcaaggtgcc gaggaagtta caaaagaacc  12061 tagagggttg ttaatgcttg ccgagttatc gtccaagggg tctctagcgc acggtgaata  12121 cactcgtggg accgtggaaa ttgccaagag tgataaggac tttgttattg gatttattgc  12181 tcaaaacgat atgggtggaa gagaagaggg ctacgattgg ttgatcatga cgccaggtgt  12241 tggtcttgat gacaaaggtg atgctttggg acaacaatac agaactgtgg atgaagttgt  12301 tgccggtgga tcagacatca ttattgttgg tagaggtctt ttcgcaaagg gaagagatcc  12361 tgtagtggaa ggtgagagat acagaaaggc gggatgggac gcttacttga agagagtagg  12421 cagatccgct taagaggggt accgagctcg aatt  // |
| LHZ1448 | LOCUS LHZ1448 12232 bp DNA circular SYN 29-NOV-2023  SOURCE  ORGANISM  COMMENT This file is created by Vector NTI  http://www.invitrogen.com/  COMMENT ORIGDB\|GenBank  COMMENT VNTDATE\|-15136799\|  COMMENT VNTDBDATE\|-15136799\|  COMMENT LSOWNER\|  COMMENT VNTNAME\|LHZ1448\|  COMMENT VNTAUTHORNAME\|Junyuan Zeng\|  FEATURES Location/Qualifiers  misc_feature 1..3103  /label=pKS  /note="/vntifkey=21"  misc_feature 3112..7868  /label=KD  /note="/vntifkey=21"  promoter 7875..8842  /label=Inulinase\Promoter  /label=Inulinase Promoter  /note="/vntifkey=29"  5'UTR 8843..9009  /label=INU1 5' UTR  sig_peptide 9010..9078  /label=Inulinase\Signal\Peptide  /label=Inulinase Signal Peptide  /note="/vntifkey=94"  CDS 9094..9936  /codon_start=1  /label=AnFaeA\optimized  /label=AnFaeA optimized  /note="/vntifkey=4"  terminator 9964..10805  /label=Inulinase\Terminator  /label=Inulinase Terminator  /note="/vntifkey=43"  promoter 10822..11407  /label=KcURA3\Promoter  /label=KcURA3 Promoter  /note="/vntifkey=29"  CDS 11408..12211  /codon_start=1  /label=KcURA3\ORF  /label=KcURA3 ORF  /note="/vntifkey=4"  ORIGIN  1 cgtaatcatg tcatagctgt ttcctgtgtg aaattgttat ccgctcacaa ttccacacaa  61 catacgagcc ggaagcataa agtgtaaagc ctggggtgcc taatgagtga gctaactcac  121 attaattgcg ttgcgctcac tgcccgcttt ccagtcggga aacctgtcgt gccagctgca  181 ttaatgaatc ggccaacgcg cggggagagg cggtttgcgt attgggcgct cttccgcttc  241 ctcgctcact gactcgctgc gctcggtcgt tcggctgcgg cgagcggtat cagctcactc  301 aaaggcggta atacggttat ccacagaatc aggggataac gcaggaaaga acatgtgagc  361 aaaaggccag caaaaggcca ggaaccgtaa aaaggccgcg ttgctggcgt ttttccatag  421 gctccgcccc cctgacgagc atcacaaaaa tcgacgctca agtcagaggt ggcgaaaccc  481 gacaggacta taaagatacc aggcgtttcc ccctggaagc tccctcgtgc gctctcctgt  541 tccgaccctg ccgcttaccg gatacctgtc cgcctttctc ccttcgggaa gcgtggcgct  601 ttctcatagc tcacgctgta ggtatctcag ttcggtgtag gtcgttcgct ccaagctggg  661 ctgtgtgcac gaaccccccg ttcagcccga ccgctgcgcc ttatccggta actatcgtct  721 tgagtccaac ccggtaagac acgacttatc gccactggca gcagccactg gtaacaggat  781 tagcagagcg aggtatgtag gcggtgctac agagttcttg aagtggtggc ctaactacgg  841 ctacactaga agaacagtat ttggtatctg cgctctgctg aagccagtta ccttcggaaa  901 aagagttggt agctcttgat ccggcaaaca aaccaccgct ggtagcggtg gtttttttgt  961 ttgcaagcag cagattacgc gcagaaaaaa aggatctcaa gaagatcctt tgatcttttc  1021 tacggggtct gacgctcagt ggaacgaaaa ctcacgttaa gggattttgg tcatgagatt  1081 atcaaaaagg atcttcacct agatcctttt aaattaaaaa tgaagtttta aatcaatcta  1141 aagtatatat gagtaaactt ggtctgacag ttaccaatgc ttaatcagtg aggcacctat  1201 ctcagcgatc tgtctatttc gttcatccat agttgcctga ctccccgtcg tgtagataac  1261 tacgatacgg gagggcttac catctggccc cagtgctgca atgataccgc gagacccacg  1321 ctcaccggct ccagatttat cagcaataaa ccagccagcc ggaagggccg agcgcagaag  1381 tggtcctgca actttatccg cctccatcca gtctattaat tgttgccggg aagctagagt  1441 aagtagttcg ccagttaata gtttgcgcaa cgttgttgcc attgctacag gcatcgtggt  1501 gtcacgctcg tcgtttggta tggcttcatt cagctccggt tcccaacgat caaggcgagt  1561 tacatgatcc cccatgttgt gcaaaaaagc ggttagctcc ttcggtcctc cgatcgttgt  1621 cagaagtaag ttggccgcag tgttatcact catggttatg gcagcactgc ataattctct  1681 tactgtcatg ccatccgtaa gatgcttttc tgtgactggt gagtactcaa ccaagtcatt  1741 ctgagaatag tgtatgcggc gaccgagttg ctcttgcccg gcgtcaatac gggataatac  1801 cgcgccacat agcagaactt taaaagtgct catcattgga aaacgttctt cggggcgaaa  1861 actctcaagg atcttaccgc tgttgagatc cagttcgatg taacccactc gtgcacccaa  1921 ctgatcttca gcatctttta ctttcaccag cgtttctggg tgagcaaaaa caggaaggca  1981 aaatgccgca aaaaagggaa taagggcgac acggaaatgt tgaatactca tactcttcct  2041 ttttcaatat tattgaagca tttatcaggg ttattgtctc atgagcggat acatatttga  2101 atgtatttag aaaaataaac aaataggggt tccgcgcaca tttccccgaa aagtgccacc  2161 tgacgtctaa gaaaccatta ttatcatgac attaacctat aaaaataggc gtatcacgag  2221 gccctttcgt ctcgcgcgtt tcggtgatga cggtgaaaac ctctgacaca tgcagctccc  2281 ggagacggtc acagcttgtc tgtaagcgga tgccgggagc agacaagccc gtcagggcgc  2341 gtcagcgggt gttggcgggt gtcggggctg gcttaactat gcggcatcag agcagattgt  2401 actgagagtg caccataaaa ttgtaaacgt taatattttg ttaaaattcg cgttaaattt  2461 ttgttaaatc agctcatttt ttaaccaata ggccgaaatc ggcaaaatcc cttataaatc  2521 aaaagaatag cccgagatag ggttgagtgt tgttccagtt tggaacaaga gtccactatt  2581 aaagaacgtg gactccaacg tcaaagggcg aaaaaccgtc tatcagggcg atggcccact  2641 acgtgaacca tcacccaaat caagtttttt ggggtcgagg tgccgtaaag cactaaatcg  2701 gaaccctaaa gggagccccc gatttagagc ttgacgggga aagccggcga acgtggcgag  2761 aaaggaaggg aagaaagcga aaggagcggg cgctagggcg ctggcaagtg tagcggtcac  2821 gctgcgcgta accaccacac ccgccgcgct taatgcgccg ctacagggcg cgtactatgg  2881 ttgctttgac gtatgcggtg tgaaataccg cacagatgcg taaggagaaa ataccgcatc  2941 aggcgccatt cgccattcag gctgcgcaac tgttgggaag ggcgatcggt gcgggcctct  3001 tcgctattac gccagctggc gaaaggggga tgtgctgcaa ggcgattaag ttgggtaacg  3061 ccagggtttt cccagtcacg acgttgtaaa acgacggcca gtgccaagct tgcatgcatc  3121 actaatgaaa agcatacgac gcctgcgtct gacatgcact cattctgaag aagattctgg  3181 gcgcgtttcg ttctcgtttt cctctgtata ttgtactctg gtggacaatt tgaacataac  3241 gtctttcacc tcgccattct caataatggg ttccaattct atccaggtag cggttaattg  3301 acggtgctta agccgtatgc tcactctaac gctaccgttg tccaaacaac ggaccccttt  3361 gtgacgggtg taagacccat catgaagtaa aacatctcta acggtatgga aaagagtggt  3421 acggtcaagt ttcctggcac gagtcaattt tccctcttcg tgtagatcag aggctatata  3481 catgccgagg tattcgatca ctctacgatg acggtctgtt agctcaacaa cttcttctaa  3541 atgctccata accgtaacgt aagaagcata actgtcaata ctgaagtcat cccagtttat  3601 tggtgctcct gttgaacagt catccactat atgttcgaat agcccaggat cacgaggagg  3661 tcctacaaac ggatacggta cagtcttctt tttatagtct gcaaattcta gaatagcatt  3721 ttttatccaa tagtgtcgaa tcgtcctggc cgttctaccg ataaaggatc caatgtgatt  3781 attagctcca ctacacgata tgttaagttt gatcgatgtc ttgttaacaa acgctaaact  3841 caagttcggc atttccaaca gcgagaagaa atcatcaatt ccatcggcta tctcttgata  3901 agtcattaga tcatatacct tctcgggatg tcgttgagtt actttatgac tagaaatctt  3961 caggttatca tcaacgtaat tgttctccaa tagctctgga gagggacata acaatacttt  4021 gattttttcc atggcctgga cttgtttccg taggaaatac ttgttctttt gtagacgttc  4081 catgatgagt ttgtatacct ctgctggaga tatccattct agatctttga tataagtttg  4141 gtatggtaaa gagttgattt tgtaggacac gtaaatctgc gctagataag tacattgtgc  4201 aaatgcctct ggtacttcgt aagacccatg ctgcgtaatt atagtattat tgagtggatc  4261 ataagcgttg tactcgtttt tgaatttaaa actgtctaat aaggccctgt aaatctctct  4321 gacttgttgt acacctttct gctcttcggg actgagatcg gatagcaatg gagcagcagt  4381 ttctgagctt tctgatgggg ctgacatggc agatgcctat tcaatgctgc cttttgtttg  4441 ggaggttatg aaatgcatct gtttacattg tatgtaatac ccttactagg caatgttata  4501 agcaaaaatc ctttgatcac atggaatatc actttatacg tgttgaaata tgcaaaaaaa  4561 cagtccccct gagctcaggg ggtggtttac gcttttgagg ctcagcagcg cgaattctct  4621 cttggggctg aagtgaaatt taaaaaagtc gcttgaggct cagccggaat tataaaacat  4681 cacctgagtc ttgagagcgc tttcactcac ctgaggctca gctgaaattt caaaaagtca  4741 cttgagccca gaaggagtgt ttcaccccct gaggctataa cgttcgttat tttaatacct  4801 aaataaacaa aaatatatgg tacaggaacg cgaggcaacg cgccgataca gggtcaatgg  4861 gtacacgaga gggtgacact aggcgtagaa agtcattagt ataaaataca gtggtatata  4921 gtagatattt agtttgtttt ccttttcttt ttctccaaaa cgatatcaga catttgtctg  4981 ataatgaagc attatcagac aaatgtctga tatcgttttt caataataat atacatcatc  5041 acaaaacaaa caaacatagc atcgcaagcc ccatcatgcc accaccgtcc gctgtgatcg  5101 caactcatgt ttccggcggt attctgcaat gaattggaga acctcgtctg agataattcc  5161 atgccattgt tcgaacaact ggaggctagg atgagctgag aaggattgag cgaccaagcg  5221 cggacttgac ggtgggctga gtggtgggct accagggctg ttaccctcct cttcaagtag  5281 ctcctcgcga gataaaggtt tattagaagg atccttcaaa acatatattt cactgcccaa  5341 tggggcttcc ttgtaaaaac ctgatataaa ggcaaataca cggtcatcta cagtcacact  5401 accatgactg tagtgtgatc tagccactgc actttgaatt tcacggtccc cagcccaatt  5461 gcccagtgag gagacatatt ttcccttctc agatctcgat aaaaaggtcg ccattaaatg  5521 tcttccaaaa tgagacttcg ggccgttcca gatcttgaag actggttcat cgacatgctg  5581 ggtaagaaac ctagagaacg ttctggccaa tgactctggt aaaaactgat gagtttgatt  5641 agttggtcta ttactggata ctgttttttc aataggcgag caaacacgca aatagtcgta  5701 taatgatatc agaagatcgc aatcaccatt cacaggatag aagttaacgt accgttcagt  5761 tctgcttttc gtttctgtca cagtagcacg cacaattggg cccagaaatg aattgttgta  5821 gatctcaaaa gtccttggat ctagattctt cagatcgctg tatctgcagc aatttccaac  5881 agctcccaga agtagcaatc ggtattccgc tcgttttgta gtggttacgc aggactgatc  5941 gaagaagcag gcaatcctgg agacaatctt ccaaatatct ttttcttttg acagaatatt  6001 agtgaattgt aatccaacca tagaagcatc gtatttatgt gtttcctcgt agcgatcaaa  6061 caaggaaact tcttgatttt taaatgggct aacaacaacc ttgtaagaag gcaatgctga  6121 ttcgatatcc ttttgcagag actctgtctt tcttagtcta acagtgaatt tgataatttt  6181 gtcatcctta tcaaaagaca gagatttgcc aattgcgctc ttgtaagagc ggtaggtatt  6241 gattttcatc tcgcgtcgga tagatagcga ctgcattgtc aagatagaga atagggacgc  6301 cagcttattt ctaatttctt tcgcatttat attaaaggtg tcagattcca gaatttcatt  6361 aatttcatta gcacactgat gaggtgtgag gtgagcagcc tccgcaaagg tagacatagg  6421 ggcattggtt ggaggccttt gaggtaccac tagagtgctg caaacatagc accgttcgag  6481 actttaaaat cttcagtttt aaaattatga aaaaaaacat cgtcctgagt tgaaacggtc  6541 gtttcaacct ccgtgtacag aaagatacat agcatatggc aagctgcacg cagcgtaaac  6601 atgccggaca actgtcattt cgtcagatca gttgatctac tctctgtgat actgcttcgt  6661 ttgtccacgg aggtcggact aactctcacc acgcttccac ggcattcgaa agaactaata  6721 ttgtatcatt gtacatatga ggaacacgca gttgaactga gcaaaccagg actcaggaaa  6781 gcaggaggta agtgctcgct tttcgtggat ccagaggaac gtgaaaattc gccttctcct  6841 cctataccgc cgtatcagat atcagagatg ccccttcatg aacttctcga gtcaggcaat  6901 gctaaattgg ttccaaatcc cgagtttgat ctaactgatc cagacgactt tcataagtgt  6961 ttctcggtca cctattcagc attatcttta atggtaccat atctgcccag agctgctcta  7021 aaggctgctc gagtgttttg taaagatcat tcaatattaa caacggatat gcttgatttg  7081 aattatcttg aagagctaat tgagttctca aaggaaactg tgaacaaaat cccagctaga  7141 atccctatag aggacatgct tctcgagcgg ggatatgtgc taccatgggt tcatggtggt  7201 acagtgaagg gaggaaagct actgaccccc aacgattgat tctttaccga atcattgcat  7261 aattcattgc ataattcatt gcagaatacc gccggaaaca tgagttgcga tcacagcgga  7321 cggtggtggc atgatggggc ttgcgatgct atgtttgttt gttttgtgat gatgtatatt  7381 attattgaaa aacgatatca gacatttgtc tgataatgct tcattatcag acaaatgtct  7441 gatatcgttt tggagaaaaa gaaaaggaaa acaaactaaa tatctactat ataccactgt  7501 attttatact aatgactttc tacgcctagt gtcaccctct cgtgtaccca ttgaccctgt  7561 atcggcgcgt tgcctcgcgt tcctgtacca tatatttttg tttatttagg tattaaaatt  7621 tactttcctc atacaaatat taaattcacc aaacttctca aaaactaatt attcgtagtt  7681 acaaactcta ttttacaatc acgtttattc aaccattcta catccaataa ccaaaatgcc  7741 catgtacctc tcagcgaagt ccaacggtac tgtccaatat tctcattaaa tagtctttca  7801 tctatatatc agaaggtaat tataattaga gatttcgaat cattaccgtg ccgattcgca  7861 cgctgcaacc gcggcacaaa cacaaacaca aacacaaaaa cgctaaatta tgcacacaag  7921 ggccggcggg gctgccggaa aaaaaaaggg aaaaatacac agacgagcgc gcacagatgg  7981 ggttaccact gcaagttaca agttgcaagt tgcacgctgg aatcagaatt ggaatcagaa  8041 ttggaattgg aattagaatt agaattaaac ttggggtagc cacgggaacg ggataactca  8101 ggaatcgctc gcaggcgtct ccgtctaggc aatcccaagg taagcctagg cactcccaca  8161 ggggaaagaa cggttgaagg caaagtagtg ctaacaattg gtaacgaatg gtaacaagtg  8221 tgtccgtctc cacctgacat ttgctagagc tggggattcc acattcttgt gctctgaatt  8281 ctcaaaccga aatggggcgt tgttacccca ggtatccggt tgtagttggc actggggatg  8341 gaaaaaaatg atgttgatgt tgagttagtt gggttgagtc aattagtgcg tgaaagtatc  8401 accacttttg tcatccggcg tttctgtgcg aatcacacac acacacacag tttattggag  8461 cacttgtttc tggcgtattc gtaattgttc tgcggtgcgg ttctgtgtgc atttttcctg  8521 gggtgtctgc cgcacctact catcacccac gccgtgggtt tgagccatgg cggaggtacg  8581 actgactggc tgcctgcctg cctgactgac tgcctgactg caggaaaaga gggtttcgaa  8641 ggaaaaactt ttcctgtgtt aatccggccg tgcgccgctg ctccaaaatc caccttcatg  8701 agaaggagtt tgaaaaaaca aaaaaattca catataaaaa gcgtatctcg agatctcaaa  8761 gtctcccttg aatcgtgttt gccagttgta actcatcctt tattcttcta ttctatctct  8821 ctctttcctt cccctaatca gcaattaaat ccggggtaag gaagaattac tactgtgtgt  8881 aacggttata tttcgttttt tatttttttt ttccattgcc atagagaaag aaaaaaaaaa  8941 aaagagagtt tgtgaagatc ttccattcga atcccataag tgacacattt aatttttttt  9001 ttgttagata tgaagttagc atactccctc ttgcttctat tggcaggagt cagtgcttca  9061 gtgatcaatt acaagagaga cggtgacact agtaagcaat tctccgctaa gtacgctttg  9121 atcttgttgg ccactgctgg tcaagctttg gctgcctcta cccaaggtat ctccgaagac  9181 ttgtacaaca gattggtcga gatggccacc atctctcaag ccgcctacgc tgacttatgc  9241 aacatcccat ccaccatcat caagggtgaa aagatttaca acgcccagac cgatatcaac  9301 ggttggatct tgagagacga cacctccaag gagatcatca ccgtcttcag aggtaccggt  9361 tccgacacca acttgcagtt ggacaccaac tacaccttga ccccattcga caccttgcca  9421 caatgcaacg actgtgaagt ccacggtggt tactatatcg gctggatctc cgtccaagac  9481 caagtcgagt ccttggtcaa gcagcaagct tcccaatacc ccgattacgc tttgaccgtc  9541 accggtcatt ctttgggtgc ctctatggct gctttgaccg ctgctcaatt gtccgccacc  9601 tacgacaacg tcagattgta caccttcggc gagccaagat ctggtaacca agctttcgcc  9661 tcctacatga acgacgcctt ccaagtttcc tctccagaga ccacccagta cttcagagtt  9721 acccactcca acgacggcat cccaaacttg ccaccagctg acgaaggtta cgctcatggt  9781 ggcgttgaat actggtccgt cgatccatac tctgcccaaa acaccttcgt ctgtaccggt  9841 gatgaggtcc aatgctgtga ggctcaaggt ggtcaaggtg ttaacgacgc ccacaccacc  9901 tacttcggta tgacctccgg tgcttgtact tggtaagcgg ccgcttaagg ccgcaagctt  9961 tgatctgatc tgcttacttt actaacgaca aaaaaaaatc aaaaaaaaaa aaacaatcag  10021 tccttctctt cttacgatat gatatgatta aatgatgcta tgaaatcatc ttcttcttaa  10081 ctttcttaaa tcttacgcgt cacttactct atatacccgt ttagctttgc ctggtcacag  10141 cgacatttta tataagtgta cgtattttct tttttttttt aaaaatttct attctaacct  10201 tagaaaagtg ccctttaaac cagctgtcct ggcactatat ctttatcatg tgccggtcgc  10261 tttccctttc cgtttccctt ttcctttcaa ttggtggcct ggaattccga actcattttc  10321 gcatctgaaa ctaattctcg aaacctttaa catcaaacaa ttgaaaagat catcatcacc  10381 agaaataaga aaaagatcaa cacaacagct aataacagta cgaaagaaag atcgctcgag  10441 tgaaaaggca gccaagaaag gtcattcgat ttgggtctag actgattata gacataccaa  10501 ttgcactcag taagaaaatg agtttcaaat ttgacgatga cggtgtggta aaagaatttc  10561 acggcaacac catcatatgc catattcctc aacaaaccga attcttcaac aaattgttgg  10621 acttctaccg ttttgcgaaa cgactttcct tctacgacaa gatcacccta cttcctcctt  10681 caagctacca cgttacgatc atgaattgct gccacgaaca cgatcgttct gagggccact  10741 ggcccaaagg aatcgatccg gacacaagca tgctgcggtg tacatcacat ctgaccaaca  10801 ttctattaat taaggatcgg tcgaattctg attggaaaga ccattctgct ttacttttag  10861 agcatcttgg tcttctgagc tcattatacc tcaatcaaaa ctgaaattag gtgcctgtca  10921 cggctctttt tttactgtac ctttgacttc ctttcttatt tccaaggatg ctcatcacaa  10981 tacgcttcta gatctattat gcattataat taatagttgt agctacaaaa ggtaaaagaa  11041 agtccggggc aggcaacaat agaaatcggc aaaaaaaact acagaaatac taagagcttc  11101 ttccccattc agtcatcgca tttcgaaaca agaggggaat ggctctggct agggaactaa  11161 ccaccatcga ctgactctat gcactaacca cgtgactaca tatatgtgat cgtttttaac  11221 atttttcaaa ggctgtgtgt ctggctgttt ccattaattt tcactgatta agcagtcata  11281 ttgaatctga gctcatcacc aacaagaaat tctaccgtaa aagtgtaaaa gttcgtttaa  11341 atcatttgta aactggaaca gcaagaggaa gtatcatcag ctagccccat aaactaatca  11401 aaggaggatg tcgactaaga gttactcgga aagagcagct gctcatagaa gtccagttgc  11461 tgccaagctt ttaaacttga tggaagagaa gaagtcaaac ttatgtgctt ctcttgatgt  11521 tcgtaaaaca gcagagttgt taagattagt tgaggttttg ggtccatata tctgtctatt  11581 gaagacacat gtagatatct tggaggattt cagctttgag aataccattg tgccgttgaa  11641 gcaattagca gagaaacaca agtttttgat atttgaagac aggaagtttg ccgacattgg  11701 gaacactgtt aaattacaat acacgtctgg tgtataccgt atcgccgaat ggtctgatat  11761 caccaatgca cacggtgtga ctggtgcggg cattgttgct ggtttgaagc aaggtgccga  11821 ggaagttaca aaagaaccta gagggttgtt aatgcttgcc gagttatcgt ccaaggggtc  11881 tctagcgcac ggtgaataca ctcgtgggac cgtggaaatt gccaagagtg ataaggactt  11941 tgttattgga tttattgctc aaaacgatat gggtggaaga gaagagggct acgattggtt  12001 gatcatgacg ccaggtgttg gtcttgatga caaaggtgat gctttgggac aacaatacag  12061 aactgtggat gaagttgttg ccggtggatc agacatcatt attgttggta gaggtctttt  12121 cgcaaaggga agagatcctg tagtggaagg tgagagatac agaaaggcgg gatgggacgc  12181 ttacttgaag agagtaggca gatccgctta agaggggtac cgagctcgaa tt  // |
